# Global return-on-investment of COVID-19 vaccines in the first year of the vaccination programme: A model-based economic evaluation

**DOI:** 10.1371/journal.pmed.1004772

**Published:** 2026-11-05

**Authors:** Hallie Benjamin, Gregory Barnsley, Oliver J. Watson, Mark Jit

**Affiliations:** 1 Faculty of Public Health and Policy, London School of Hygiene and Tropical Medicine, London, United Kingdom; 2 Department of Infectious Disease Epidemiology, Faculty of Epidemiology and Population Health, London School of Hygiene and Tropical Medicine, London, United Kingdom; 3 MRC Centre for Global Infectious Disease Analysis, Imperial College London, London, United Kingdom; 4 Department of Global and Environmental Health, School of Global Public Health, New York University, New York, United States of America; University of Bern Faculty of Natural Sciences: Universitat Bern Philosophisch-naturwissenschaftliche Fakultat, SWITZERLAND

## Abstract

**Background:**

COVID-19 vaccines played a critical role in reducing global health burden during the COVID-19 pandemic, but their rapid development required extraordinary effort. This study aims to evaluate their global return-on-investment (ROI) during the first year of the vaccination programme (8 December 2020–8 December 2021) to provide insights into the economic benefits relative to the costs of vaccine development, manufacturing, and delivery.

**Methods and findings:**

Public health outcomes of COVID-19 vaccination, including life years and quality-adjusted life years (QALYs) gained from averted deaths, infections, and hospitalizations, were estimated. Health benefits were valued using two approaches: i) cost-benefit analysis and ii) cost-effectiveness analysis. These benefits were compared to the costs of vaccine development, manufacturing, and delivery to estimate the ROI under both approaches. Probabilistic sensitivity analyses were conducted to assess the impact of parameter uncertainty on ROI estimates. Vaccine development and delivery cost $79.4 billion, but the health and economic benefits of vaccination were valued between $6.43 and $64.3 trillion. High-income countries saw the greatest health benefits per-person vaccinated, and low-income countries gained the greatest nonhealth benefits per-person vaccinated, when expressed as a proportion of gross domestic product per capita. This estimated ROIs of $59.7–$809 per dollar invested in the base case analysis. ROIs ranged between $60.3–$753 across probabilistic sensitivity analyses for both approaches. Limitations include reliance on previous analyses of COVID-19 vaccination impact and a time horizon limited to the first year of COVID-19 vaccination.

**Conclusions:**

Rapid COVID-19 vaccine development and delivery were an excellent investment. Vaccine development and equitable vaccine distribution globally should be prioritized before and during global health crises.

## Author summary

**Why was this study done?:**

- Extraordinarily rapid and large investments were made globally into developing COVID-19 vaccines during the pandemic in 2020.
- COVID-19 vaccination significantly reduced morbidity and mortality worldwide in its first year (December 2020–December 2021), but it remains unclear whether these benefits were worth the investments.

**What did the researchers do and find?:**

- Using previously published data from an epidemiological transmission model, we quantified the global health, productivity, and healthcare benefits of COVID-19 vaccination in monetary terms.
- We compared these benefits with the global costs of vaccine research, development, manufacturing, and delivery, and assessed differences across World Bank income groups.
- The benefits of global COVID-19 vaccination in its first year were at least 70 times greater than the costs, although substantial disparities existed across income groups.

**What do these findings mean?:**

- Our findings mean that the investments made into COVID-19 vaccines largely paid off, promoting the need for future investments into vaccines for both health and economic purposes.
- The disparities observed between income groups reflect the need for equitable vaccine distribution and the sharing of vaccines and vaccine-related information across borders.
- Despite study limitations, including reliance on previous analyses of COVID-19 vaccine impact and a year-long time horizon, the large net positive return suggests that investments in COVID-19 vaccines and vaccination programs were highly worthwhile.

## Abstract 3

In this model-based economic evaluation, Hallie Benjamin and colleagues compare the costs of vaccine development, manufacturing, and delivery with estimated health and economic benefits during the first year of the COVID-19 vaccination programme. The study suggests it was associated with substantial value relative to investment across countries, with estimated benefits far exceeding costs and highlighting the potential importance of vaccine development and equitable distribution during global health emergencies.

## Background

After SARS-CoV-2 was identified as the novel coronavirus responsible for COVID-19 in 2020, an extraordinary global effort was launched by governments, pharmaceutical companies and nonprofit organizations to rapidly develop vaccines. Remarkably, the first COVID-19 vaccines received emergency use authorization within 12 months of viral identification, a process that typically takes a decade or more. These vaccines proved to be highly effective in preventing severe illness and death, with a modeling study finding that they averted between 14–20 million COVID-19-related deaths in the first year of use [1]. However, despite their clear health impact, the economic value of these vaccines—particularly in relation to the massive investments in their rapid development—remains unexamined on a global level.

Although numerous economic evaluations of COVID-19 vaccines have informed vaccine procurement and deployment at the country-level, none have assessed the value for money of vaccine development itself, rather than just vaccine procurement. A systematic review of economic evaluations for COVID-19 vaccines conducted in 2023 [2] identified 25 full economic evaluation studies, including cost-minimization, cost-benefit, cost-effectiveness, and cost-utility analyses. However, they all compared country-level benefits to the cost of the national programme rather than the cost of vaccine development [2]. While a recent study looked at COVID-19 vaccination’s impact on global gross domestic product (GDP) based on regression analysis, it did not look at the return-on-investment (ROI) of vaccine development itself [3]. Additionally, the growing literature of economic evaluations on COVID-19 vaccines has primarily assessed the value of vaccines on the country level, limiting their analyses to national procurement and delivery costs. These analyses do not capture the full scale of investments that enabled vaccine availability in the first place. COVID-19 vaccine development was unique because the vaccines’ rapid development required substantial, coordinated public investment across countries, including direct funding of research and development and Advanced Purchase Agreements (APAs) that de-risked private-sector production. These investments were made upstream of national vaccination programmes and generated global health and economic benefits that extend beyond any single country’s health system [4]. As governments and global institutions consider how best to protect global populations in future pandemics, understanding the global economic returns to these upstream investments is critical.

ROI analyses evaluate the economic benefits of an intervention relative to its costs, often using a cost-benefit approach that monetizes health and nonhealth impacts [5]. Despite the complexity of conducting ROI analyses for vaccine impact, including selecting the appropriate methodology, ROI analyses are vital for demonstrating the health and economic value of vaccines and building strong investment arguments [6]. Estimating the global ROI of COVID-19 vaccine development and delivery is therefore relevant and necessary to pandemic preparedness planning, including decisions about whether similar public investments should be completed for future global health emergencies.

Quantifying the ROI of COVID-19 vaccine development may also inform ongoing debates regarding vaccine pricing. During the pandemic, concerns were raised that vaccines developed with public funding were subsequently sold back to governments at higher prices and at variable prices between governments, raising questions about equity, affordability, and corporate profits [7,8]. By estimating the total economic and health surplus generated by COVID-19 vaccines in monetary value relative to their development and distribution costs, conducting a ROI analysis may provide evidence that the health and economic benefits provided by the vaccines were worth their cost.

Recently, there have been promising steps toward greater equity in pandemic vaccine distribution, such as the signing of the World Health Organization’s Pandemic Treaty [9]. However, some funding streams for pandemic vaccine research have also been cut [10]. In the event of future pandemics, a clearer understanding of the ROI from COVID-19 vaccines could play a critical role in guiding decisions around comparable investments in rapid vaccine development. Furthermore, COVID-19 vaccine benefits are unequally distributed across countries due to access inequalities, and the economic implications of this inequity have not been fully enumerated.

To address these evidence gaps, we aimed to evaluate the global ROI for COVID-19 vaccines over the first year of their use (8 December 2020–8 December 2021), when the vaccines had the greatest impact due to limited pre-existing immunity, by comparing the vaccines’ benefits to their development and distribution costs. We also aimed to estimate the wider societal benefits of COVID-19 vaccination, including reduced mortality and morbidity, as well as improved productivity, and to compare these benefits generated across World Bank income groups.

## Methods

### Overview

The ROI of COVID-19 vaccination over the first year of the vaccination programme globally was determined by estimating the value of its public health outcomes and then comparing them to the investments made into COVID-19 vaccine development and delivery using two standard approaches: a cost-benefit analysis (CBA) and a cost-effectiveness analysis (CEA).

In the CBA, health benefits are expressed in monetary terms based on individuals’ willingness-to-pay (WTP) for them. This reflects the maximum amount of consumption that an individual is willing to forgo for the benefit. In the CBA, vaccine-attributed reductions in mortality are valued using estimates of individuals’ WTP for small reductions in mortality risk, aggregated to derive the value of a statistical life (VSL). VSL estimates are typically derived from stated (i.e., surveys) and revealed (i.e., data about real-world risk-trade-off situations) preferences [11]. Our CBA framework also incorporates the monetary value of other benefits such as morbidity reductions and productivity gains.

CBA is commonly associated with a welfarist framework, which evaluates the desirability of an option according to its effect on individual utility [12]. However, Hammitt [13] notes that CBAs are not always strictly welfarist, since they involve analytical choices that correct individual preferences for various perceived biases. Depending on their justification, these corrections reflect either welfarist or extra-welfarist principles. Moreover, alternative formulations of welfarism from the CBA approach we used aggregate individual preferences differently; for example, some incorporate distributive concerns [14].

The CEA typically evaluates interventions by comparing their incremental costs and health gains separately. The ratio of the two (known as the incremental cost-effectiveness ratio or ICER) is then compared with a cost-effectiveness threshold representing the opportunity cost of additional health gains. These opportunity costs are the value of health foregone elsewhere in the health system when resources are allocated to the next best alternative intervention or health programme [15]. Rather than monetizing health gains directly, CEAs use nonmonetary health measures such as quality-adjusted life years (QALYs), which combine both the quality and quantity of life into a single metric [16]. It is therefore considered an extra-welfarist approach, since it differs from welfarism in that it does not assign value solely based on individual preferences but assigns an intrinsic value to health [12]. In our analysis, we adapt this approach to produce an ROI by estimating QALYs gained in monetary terms using estimates of the health opportunity costs of healthcare spending by country [17].

To accurately assess the impact of COVID-19 vaccination on mortality and morbidity, we used the results of a previously published global transmission model-based analysis of COVID-19 vaccination impact by Watson and colleagues [1]. This study used an age-structured, country-specific transmission model of SARS-CoV-2 to estimate the number of infections, hospitalizations, and deaths averted by COVID-19 vaccination during the first year of global rollout (8 December 2020–8 December 2021) for 17 discrete age groups (0−5, 5−10,..., 75−80, 80+) (Table 1). The model is a deterministic compartmental transmission model stratified by age and vaccination status, which models SARS-CoV-2 infection, progression to severe disease, hospitalization, and death. The model accounts for age-specific susceptibility, severity, and infection fatality risk and incorporates country-specific demographic structures, contact patterns, and vaccination roll-out. The model was fit to excess all-cause mortality [41] to mitigate biases arising from under-ascertainment and differences in COVID-19 death reporting systems globally. Model parameters were inferred separately for each country. The fitting framework explicitly incorporated uncertainty in key epidemiological parameters, including transmission intensity, infection fatality ratios, and vaccine effectiveness against severe outcomes.

**Table 1:** Input parameters used in the economic analysis.

Parameter	Value	Range used for sensitivity analysis	Sources	Notes and assumptions
**Health benefits**				
COVID-19 vaccine impact on public health outcomes	–	–	Watson and colleagues [1]	Available for discrete age groups (0–5, 5–10,..., 75–80, 80+) for 173 countries.
Cohort life expectancy	–	–	WHO Global Health Observatory indicator views [18]	Dependent on country and age group.
Discount rate	3% and 0%	–	WHO-CHOICE [19]	A discount rate is applied to determine the current value of costs and benefits that occur in the future, which enables the comparison of costs and benefits that occur at different times.
Probability of asymptomatic infection				
Individuals aged 0–20 years	0.467	0.320–0.620	Sah and colleagues [20]	
Individuals aged 20–60 years	0.321	0.222–0.439	Sah and colleagues [20]	
Individuals aged 60 + years	0.197	0.127–0.294	Sah and colleagues [20]	
Quality-adjusted life years (QALYs) gained				
Symptomatic COVID-19 infection	0.008	± 25% of mean	Sandmann and colleagues [21] Van Hoek and colleagues [22]	
COVID-19 non-ICU hospitalization	0.0201	± 25% of mean	Sandmann and colleagues [21] Halpin and colleagues [23]	
COVID-19 ICU hospitalization	0.15	± 25% of mean	Sandmann and colleagues [21] Cuthbertson and colleagues [24]	
COVID-19 infection duration	5 days	–	Watson and colleagues [1]	Assumed the same values used in original modeling study for consistency.
COVID-19 hospitalization duration	12 days	–	Watson and colleagues [1]	Assumed the same values used in original modeling study for consistency.
**Value of health benefits**				
Mean reference USA value of statistical life (VSL), USD 2021	10,519,303.52	$1,912,600.64–$19,126,006.40	United States Department of Agriculture Economic Research Service [25]	Estimate of VSL for 2020 used for analysis
Gross national income per capita (GNIpc), USD 2021	Country-specific	–	World Bank [26]	Atlas method
Income elasticity	1	1.5	Masterman and colleagues [27]	
Gross domestic product (GDP), USD 2021	Country-specific	–	World Bank [28]	Estimate of GDP for 2020 used for analysis
Percentages of GDP for opportunity cost thresholds				
High-income countries	0.68	0.50–0.88	Pinchon-Riviere and colleagues [17]	Represents health opportunity costs of healthcare spending in each country, i.e., how much health spending results in gaining a year of life in perfect health
Upper-middle income countries	0.58	0.44–0.76	Pinchon-Riviere and colleagues [17]	Represents health opportunity costs of healthcare spending in each country, i.e., how much health spending results in gaining a year of life in perfect health
Lower-middle income countries	0.35	0.23–0.48	Pinchon-Riviere and colleagues [17]	Represents health opportunity costs of healthcare spending in each country, i.e., how much health spending results in gaining a year of life in perfect health
Lower income countries	0.24	0.18–0.32	Pinchon-Riviere and colleagues [17]	Represents health opportunity costs of healthcare spending in each country, i.e., how much health spending results in gaining a year of life in perfect health
**Productivity costs**				
Gross domestic product per capita (GDPpc), USD 2021	Country-specific	–	World Bank Data [29]	GDPpc divided by 365.25 days was used as a proxy for average daily income. The estimate of GDPpc for 2020 was used for analysis.
**Friction periods**				
Upper-middle, lower-middle-, and low-income countries	91.251 days	–	Krol and Brouwer [30]	A friction period of 3 months was used for LMICs as recommended by Krol and Brouwer (2014), due to a lack of reliable friction period estimates.
High-income countries	60.6 days	0–365 days	Hanly and colleagues [31]	Given that estimated friction periods were provided for only Organisation for Economic Co-operation and Development countries, to improve consistency with LMIC data, the European average friction period value of 60.6 days was used.
**Healthcare costs per day**				
Upper-middle, lower-middle-, and low-income countries, USD 2021	76.6	–	Torres-Rueda and colleagues [32]	Used the “Hospital-based (severe cases) per day of hospitalization (severe case)” values.
High-income countries, USD 2021	647.09	–	WHO-CHOICE [33]	Used the “Cost per inpatient bed day by hospital level (without drugs)” values for primary hospitals.
**GDP deflator indices**				
2010	92.1	–	World Bank [34]	
2019	107.3	–	World Bank [34]	
2020	108.7	–	World Bank [34]	
2021	113.6	–	World Bank [34]	
2022	121.6	–	World Bank [34]	
**Vaccine development and delivery costs**				
Vaccine development costs (including Advanced Purchasing Agreements, corporate investments, and manufacturing costs)	–	–	Wouters and colleagues [35] Florio and colleagues [36] Publicly available data [37]	Development costs include investments made by public and private sectors, philanthropic and other funders, either directly to organizations and companies conducting research, development and manufacturing, to intermediaries or through Advanced Purchasing Agreements.
EUR to USD exchange rate	0.845	–	World Bank [38]	Used for 2020.
Vaccine delivery cost per person vaccinated, USD 2021	3.70	–	Griffiths and colleagues [39]	Due to the lack of consistent data and proxy cost data for COVID-19 vaccine delivery costs, a single value for all vaccines delivered was used. This value was applied to the vaccine coverage rates of each country, which were the vaccine coverage rates used in the modeling of COVID-19 vaccine impact, to obtain the total delivery costs [1].
**Other**				
World Bank income group classifications	–	–	World Bank [40]	Used 2022 classifications, which are based on 2,021 data.
Vaccine coverage	–	–	Published literature [1]	Proportion of the population with a full dose in the modeled countries by December 8, 2021.

To estimate vaccine impact, the fitted model was simulated under two scenarios: (i) the observed vaccination rollout and (ii) a counterfactual scenario in which no COVID-19 vaccines were administered, while all other factors, including nonpharmaceutical interventions, behavioral changes, and variant emergence, were assumed to remain as observed. The difference between these scenarios yielded estimates of infections, hospitalizations, and deaths averted by vaccination. Uncertainty in epidemiological outcomes was propagated by drawing 100 posterior samples per country from the fitted model. These samples were used directly as inputs to the economic evaluation, ensuring that uncertainty from the transmission model was carried through to all health and ROI estimates. Further methodological details are provided in S1 Appendix and in Watson and colleagues [1]. Given that nonpharmaceutical interventions, behavioral changes, and variant emergence were assumed to remain as historically observed, the benefits of COVID-19 vaccination considered in this study are limited to those related to the direct reduction in COVID-19 morbidity and mortality, and do not capture costs and benefits arising from associated policy or behavioral changes enabled by vaccination (such as relaxation of lockdowns).

Other data required for our analysis were collected from online secondary sources, including the World Bank, World Health Organization (WHO), and published papers (Table 1). Our analysis includes 173 countries (i.e., the 180 countries analyzed by Watson and colleagues, except for countries lacking World Bank economic indicators (Cuba, Eritrea, North Korea, South Sudan, Venezuela, and Yemen) or WHO life expectancy data (Aruba)).

### Study perspective and horizon

The study follows a societal perspective at a global level. Only true economic costs and benefits were considered, which excludes fiscal transfers between entities such as government vaccine purchases from manufacturers or intermediaries, including COVID-19 Vaccines Global Access (COVAX) [42]. Under this perspective, all costs and benefits associated with the vaccines are included, regardless of who incurred them [43]. The time horizon includes COVID-19 vaccine development and delivery costs between 2020 and 2021, and the health and economic benefits generated by the vaccines delivered between 8 December 2020–8 December 2021 (including future life years lost from deaths in 2020–2021). All costs are reported in US$2021.

### Benefits

The health benefits generated from COVID-19 vaccines were evaluated in terms of deaths averted and life years (LY) and QALYs gained. LYs gained were determined by multiplying age group-specific average life expectancies for each country by the number of deaths averted, with a 3% discount rate applied to reflect time preferences for current health benefits. Undiscounted and discounted QALYs gained were estimated by combining averted QALY losses from infections, hospitalizations, and deaths. QALY loss estimated ranged between 0.15 and 0.008 per symptomatic case (including hospitalizations and intensive care unit admissions) and perfect health (QALY of 1.0) was assumed for averted deaths. Further details are in S1 Appendix.

For the CBA, deaths averted were valued using country-specific VSLs, which represent the marginal rate of substitution between income and mortality risk, derived from individuals’ WTP for small reductions in mortality risk. Following Robinson and colleagues [44], VSLs were derived by adjusting a US reference value using country-specific gross national income (GNI) per capita and an income elasticity of 1. For the CEA, undiscounted and discounted QALYs gained were valued by applying opportunity cost thresholds, which are based on income-group dependent percentages of gross domestic product (GDP) per capita and represent the marginal productivity of health spending. Further details are in S1 Appendix.

Economic benefits generated during the first year of the COVID-19 vaccination programme were assessed from a societal perspective in both the CBA and CEA, by estimating both averted productivity losses and healthcare costs. Productivity costs averted due to reduced COVID-19 related absenteeism and deaths were calculated using the friction cost (FC) approach. This approach was chosen over the human capital approach to be conservative in estimating vaccine benefits; the FC approach assumes that sick or deceased workers are replaceable and only considers productivity costs incurred during the period when the position is unfilled (friction period). These costs represent the output losses borne by employers during the friction period, presumably falling outside an individual’s marginal rate of substitution between wealth and mortality risk (since they do not alter the individual’s health or consumption) and are therefore not captured by VSL estimates. Healthcare costs averted were estimated by multiplying the number of hospitalizations averted by costs per hospitalization. These healthcare costs are assumed to be borne by health systems or insurers rather than incurred out-of-pocket by individuals and thus do not overlap with VSL-based valuations. We acknowledge that individuals sick with COVID-19 likely incurred some out-of-pocket costs, but for the purposes of this analysis we assume these are captured within VSL estimates. GDP deflator indices were used to control for inflation for costs that were reported in years other than 2021. Further details are in S1 Appendix. Since costs and benefits were only considered for one year, discounting was not applied to the costs. However, since life years gained extend beyond this first year, discounting was applied to QALY estimates to adjust for preference for immediate benefits.

Overall, the health and economic benefits considered in the CBA included averted mortality valued by VSLs and reduced third-party healthcare and productivity costs due to averted mortality and morbidity; while the CEA’s health and economic benefits included averted mortality and morbidity measured using QALYs and reduced third-party healthcare and productivity costs due to averted mortality and morbidity.

### Vaccine development and delivery costs

Development costs included costs from the pandemic’s start (2020), but excluded any costs related to the basic research that contributed to the vaccine development prior to the pandemic, as these were regarded as sunk costs. Vaccine costs considered in this study include both public and corporate investments into the basic research, development, and manufacturing of COVID-19 vaccines, as well as APAs that were used to accelerate the development of the vaccines. Vaccine research includes funding for development and testing of vaccine candidates and clinical trials [35–37]. Development cost breakdowns are in Table A in S1 Appendix. Costs incurred in 2020 were converted to 2021 USD using GDP deflator indices.

Vaccine delivery costs reflect the costs incurred to deliver vaccines to individuals, including human resources and vaccine transport. A single vaccine delivery cost reflecting the average delivery cost for a fully vaccinated individual was used and was multiplied by the number of fully vaccinated individuals to obtain the overall cost of delivering vaccines in each country [39]. This delivery cost estimate includes human resources for vaccine administration, training and supervision, cold-chain infrastructure and maintenance, transportation and logistics, social mobilization and communication activities, data systems and monitoring, waste management, and other service delivery-related inputs. The estimate was obtained from a study modeling COVID-19 vaccine delivery costs across lower-middle income countries (LMICs) using a standardized, aggregate delivery costing framework informed by global data and country-level parameters and was applied to all countries included in the current study. Immunization-related costs including side effects and productivity losses from attending vaccination, were not included in this study.

### ROI estimate

To determine the ROI, the value of the benefits was compared to the costs using the following general equation: ROI = (benefits–costs)/costs [45].

Two ROI estimates were obtained: i) for the CBA, ROI where the benefits include VSLs gained and productivity and healthcare costs averted and ii) for the CEA, ROI where the benefits include the value of the QALYs gained and productivity and healthcare costs averted.

### Sensitivity analyses

A probabilistic sensitivity analysis (PSA) was used to propagate parameter uncertainty to the ROI estimates. The PSA used Latin Hypercube Sampling (LHS) to generate 100 sets of values by sampling from the uncertainty distributions of the following parameters: (a) USA VSL value, (b) percentages of GDP used to calculate opportunity cost thresholds for each World Bank income group, (c) QALY losses per infection, hospitalization, and death, and (d) friction periods in high-income countries (HIC). We then used the 100 epidemic model fits previously generated in Watson and colleagues. Each model fit represented a distinct set of epidemic parameters drawn from their respective uncertainty distributions. These outputs were randomly paired with the 100 sets of health-economic parameters sampled using LHS to capture uncertainty in both epidemiological and economic parameters. Median and 95% credible intervals for the combined 100 output sets were then calculated to determine the PSA results. Further details can be found in S1 Appendix. Partial rank correlation coefficients (PRCC) were calculated to assess the relationship between parameters and epidemiological model samples with reported uncertainties and their outcomes, and consequently the effects of parameter uncertainty on ROI estimates. Outcomes assessed in the PSA were QALYs, friction costs, and VSLs.

A few additional sensitivity analyses were conducted. The first was a sensitivity analysis on the CBA, which assessed how the exclusion of productivity and healthcare costs averted in the valuation of the vaccines under this perspective shifted the ROI estimate. This additional analysis is aimed to explore how valuing the vaccines based on their ability to reduce mortality risk alone impacts the ROI estimate. Another CBA-related sensitivity analysis was conducted to vary the monetary value of lives saved by applying the value of statistical life years (VSLY), as per Robinson and colleagues (2019) [44]. Country-specific VSLY values were obtained by dividing the VSL by the undiscounted future life expectancy of each country and then multiplying this value by the number of undiscounted life years gained from COVID-19 vaccination. In addition to the country-specific VSLYs, we also estimated an ROI using a uniform global VSLY. This VSLY was derived from a population-weighted mean VSLY from the country-specific VSLYs included in the analysis. This additional analysis was conducted as a contrasting scenario to the base case with country-specific VSLYs, acknowledging that differences in the marginal value of money across country income groups could affect the ROI estimates and the distribution of benefits across income settings, since an equivalent increase in income may gain more utility in a lower-income country. Lastly, we also explored how increasing income elasticity to 1.5 for LMICs and LICs affects the CBA ROI estimates, as per Robinson and colleagues (2019)’s recommendations [44]. All additional sensitivity analyses, excluding the first, included healthcare and productivity costs in the vaccination benefits.

An overview of all costs and benefits included under each approach, as well as sensitivity analyses, can be found in Fig 1.

**Fig 1:**
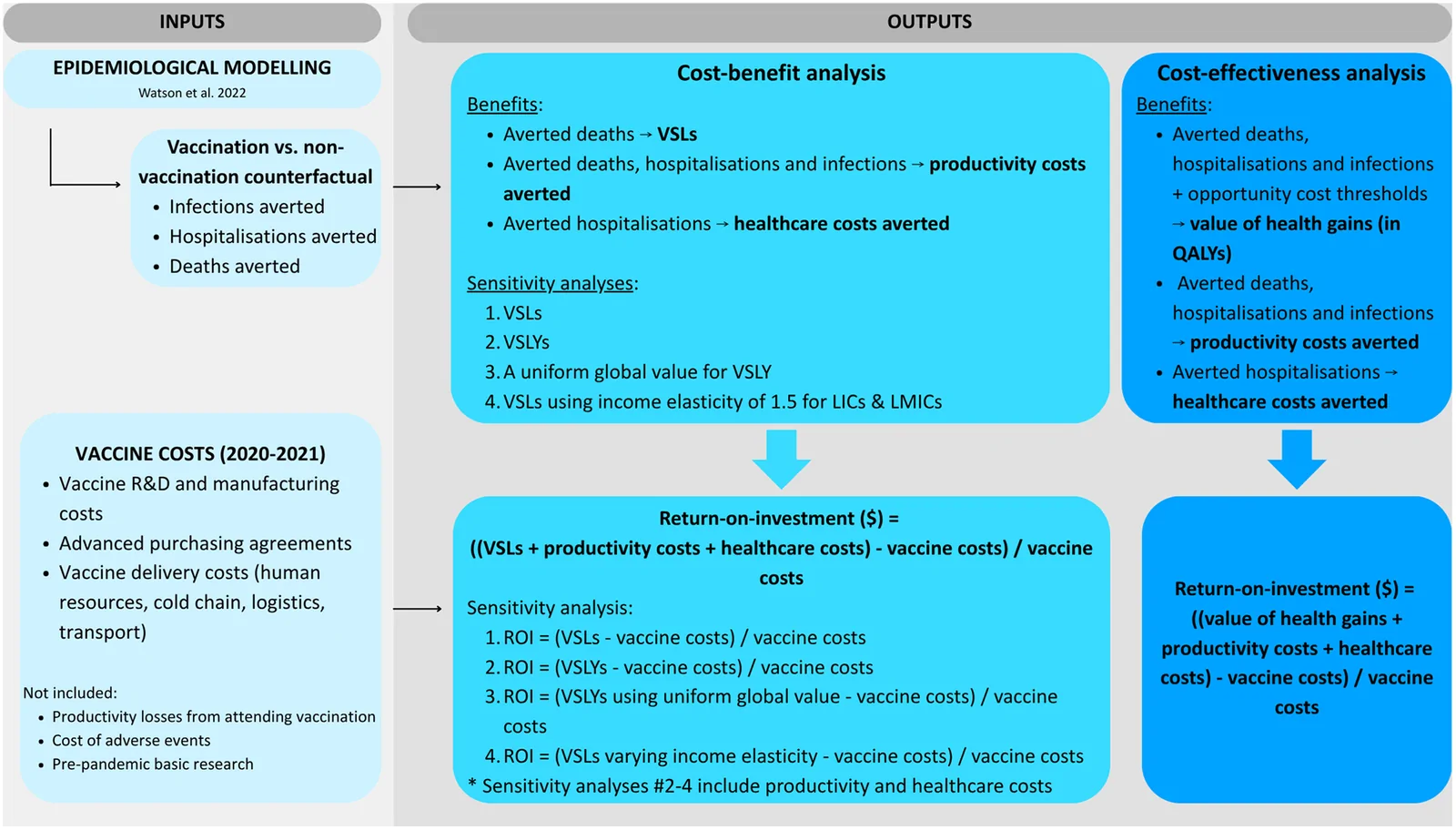
An overview of all study inputs, outputs, costs, and benefits. Abbreviations: VSL, value of statistical life; QALY, quality-adjusted life year; R&D, research and development; VSLY, value of statistical life year.

### Statistical analysis

Analyses were conducted using R (version 2024.04.2+764). This paper was developed in alignment with the Consolidated Health Economic Evaluation Reporting Standards (CHEERS) 2022 guidelines [46].

No generative artificial intelligence tools were used in the creation of this manuscript.

## Results

The total cost of COVID-19 vaccine development and delivery is estimated at $79.4 billion. Publicly announced public and nonprofit investments into COVID-19 vaccine development between 2020 and 2021 are estimated at $13.2 billion. APAs for COVID-19 vaccines totaled an estimated $42.7 billion. Corporate and private sector investments in development and manufacturing efforts are estimated at $14.1 billion. Additional manufacturing costs, excluding those included in public and corporate investments and APAs, totaled an estimated $389 million. With an estimated delivery cost of $3.70 per-person vaccinated, total delivery costs for all doses delivered in the first year of vaccination are estimated to be $8.94 billion.

From 8 December 2020 to 8 December 2021, COVID-19 vaccines are estimated to have generated 353 million (95% credible interval [CrI] [341,362]) undiscounted LYs or 252 million (95% CrI [243,258]) discounted LYs globally. LYs gained per-person vaccinated were greatest in HICs and lowest in low-income countries (LICs) (Table B in S1 Appendix). Vaccination was also estimated to have averted 1.50 billion (95% CrI [1.45,1.54]) COVID-19 infections and 51.5 million (95% CrI [50.0,52.7]) hospitalizations globally (Table C in S1 Appendix). We estimated that HICs and LICs experienced the greatest number of infections and hospitalizations averted per-person vaccinated, with relatively more infections averted per-person vaccinated in LICs.

Deaths, infections, and hospitalizations averted by COVID-19 vaccination generated an estimated 364 million (95% CrI [352,373]) undiscounted QALYs globally. Applying a discount rate of 3% reduced this value to 262 million (95% CrI [253,269]) QALYs gained. QALYs gained per-person vaccinated decreased with income group level (Table B in S1 Appendix).

### Value of health benefits

In the CBA, it was estimated that COVID-19 vaccination generated economic value of $63.5 trillion (95% CrI [61.4,66.6]) in VSLs from averting deaths. HICs experienced the highest gains in VSLs, while LICs experienced the lowest gains. However, when expressed as per-person vaccinated as a percentage of GDP per capita (GDPpc), HICs and LICs incurred higher gains than upper-middle income countries (UMICs) and LMICs. Table 2 reports VSLs globally and across the World Bank income groups.

**Table 2:** Estimates of values of a statistical life (VSLs) gained by averting deaths through COVID-19 vaccination expressed in three ways. Abbreviations: CrI, credible interval; GDP, gross domestic product; GDPpc, gross domestic product per capita.

	GDP (Trillion ($))	VSLs gained		
		**Total** **(% of GDP) (95% CrI)**	**Per-person vaccinated** **(Thousands ($)) (95% CrI)**	**Per-person vaccinated (% of GDPpc)** **(95% CrI)**
**Worldwide**	77.8	81.7 (79.0,85.7)	26.3 (25.4,27.6)	214 (207,224)
**World Bank income group**				
**High**	58.9	93.7 (90.3,98.8)	66.4 (63.9,69.9)	136 (131,143)
**Upper-middle**	9.70	56.5 (54.3,57.9)	9.59 (9.24,9.85)	112 (108,116)
**Lower-middle**	8.74	32.8 (30.8,34.0)	2.89 (2.72,3.00)	110 (104,114)
**Low**	0.436	4.24 (3.99,4.43)	0.808 (0.760,0.844)	119 (112,124)

Total value of statistical lives (VSLs) gained from averted COVID-19 deaths is presented as percentages of gross domestic product (GDP), per-person vaccinated in absolute terms, and per-person vaccinated as a percentage of gross domestic product per capita (GDPpc) as medians with 95% credible intervals. GDP reported is the reported total GDP worldwide and per income group for the 187 countries included in the analysis with available economic indicator data. Global and income-group estimates are reported as medians from simulated posterior draws. Income-group medians do not necessarily sum to the global median due to the nonadditivity of medians under uncertainty.

In the CEA, the estimated global value of the morbidity and mortality that COVID-19 vaccination averted was $5.65 trillion (95% CrI [5.46,5.92]). After applying a discount rate of 3%, the value of these health gains decreased to $4.03 trillion (95% CrI [3.90,4.23]). HICs received the greatest health benefits expressed in terms of dollars saved per-person vaccinated as a percentage of GDP, with LICs experiencing the lowest returns. Table 3 reports the discounted and undiscounted value of health gains globally and across the World Bank income groups.

**Table 3:** Estimates of value of quality adjusted life years (QALY) gained by averting deaths and morbidity through COVID-19 vaccination expressed in three ways. Abbreviations: CrI, credible interval; GDP, gross domestic product; GDPpc, gross domestic product per capita.

	GDP (Trillion ($))	Undiscounted value of QALYs gained			Discounted value of QALYs gained		
		Total (% of GDP) (95% CrI)	Per-person vaccinated ($) (95% CrI)	Per-person vaccinated (% of GDPpc) (95% CrI)	Total (% of GDP) (95% CrI)	Per-person Vaccinated ($) (95% CrI)	Per-person vaccinated (% of GDPpc) (95% CrI)
**Worldwide**	77.8	7.27 (7.02,7.61)	2,338 (2,261,2,451)	19.0 (18.4,19.9)	5.19 (5.02,5.44)	1,669 (1,616,1,751)	13.6 (13.1,14.2)
**World Bank income group**							
**High**	58.9	8.70 (8.38,9.16)	6,163 (5,935,6,486)	12.6 (12.2,13.3)	6.20 (5.98,6.53)	4,393 (4,236,4,624)	9.01 (8.69,9.49)
**Upper-middle**	9.70	4.06 (3.90,4.18)	692 (664,712)	8.12 (7.79,8.35)	2.97 (2.85,3.05)	505 (485,519)	5.92 (5.69,6.09)
**Lower-middle**	8.74	1.45 (1.37,1.51)	128 (121,133)	4.87 (4.59,5.07)	1.05 (0.994,1.10)	92.9 (87.6,96.6)	3.54 (3.34,3.68)
**Low**	0.436	0.115 (0.109,0.120)	21.9 (20.8,22.9)	3.22 (3.06,3.35)	0.0853 (0.0811,0.0889)	16.3 (15.5,17.0)	2.39 (2.27,2.49)

Total undiscounted and discounted QALYs gained from averted COVID-19 deaths, infections and hospitalizations, and total productivity and healthcare costs averted from COVID-19 vaccines are presented as percentages of actual GDP, per-person vaccinated in absolute terms, and per-person vaccinated as a percentage of GDPpc as medians with 95% credible intervals. GDP reported is the total GDP worldwide and per income group for the 187 countries included in the analysis with available economic indicator data. Global and income-group estimates are reported as medians from simulated posterior draws. Income-group medians do not necessarily sum to the global median due to the nonadditivity of medians under uncertainty.

### Productivity and healthcare costs

Productivity costs saved from averted COVID-19 morbidity and mortality are estimated to be $564 billion (95% CrI [541,586]) and 0.73% of global GDP (95% CrI [0.70,0.75]), with LICs experiencing the greatest returns in dollars expressed as a percentage of GDPpc (Table 2). COVID-19 vaccines were estimated to yield additional savings of $222 billion (95% CrI [213,229]) in healthcare costs, which is an estimated 0.29% of global GDP (95% CrI [0.27,0.30]). These savings in terms of a percentage of GDPpc were greatest in LICs and lowest in LMICs (Table 4).

**Table 4:** Estimates of the value of benefits of COVID-19 vaccines, including averted healthcare and productivity cost, expressed in three ways. Abbreviations: CrI, credible interval; GDP, gross domestic product; GDPpc, gross domestic product per capita.

	GDP (Trillion ($))	Productivity costs averted		
		Total (% of GDP) (95% CrI)	Per-person vaccinated ($) (95% CrI)	Per-person vaccinated (% of GDPpc) (95% CrI)
**Worldwide**	77.8	0.726 (0.695,0.754)	234 (224,243)	1.90 (1.82,1.97)
**World Bank income group**				
**High**	58.9	0.826 (0.788,0.865)	585 (558,612)	1.20 (1.14,1.26)
**Upper-middle**	9.70	0.490 (0.476,0.503)	83.5 (81.1,85.7)	0.980 (0.952,1.01)
**Lower-middle**	8.74	0.340 (0.326,0.353)	29.9 (28.8,31.3)	1.14 (1.09,1.18)
**Low**	0.436	0.0562 (0.0541,0.0587)	10.7 (10.3,11.2)	1.57 (1.51,1.64)
		**Healthcare costs averted**		
**Worldwide**	77.8	0.285 (0.273,0.295)	91.7 (88.0,94.8)	0.745 (0.715,0.770)
**World Bank income group**				
**High**	58.9	0.326 (0.310,0.338)	231 (220,240)	0.474 (0.451,0.491)
**Upper-middle**	9.70	0.192 (0.184,0.198)	32.7 (31.3,33.7)	0.384 (0.367,0.395)
**Lower-middle**	8.74	0.124 (0.115,0.127)	10.9 (10.2,11.2)	0.415 (0.386,0.428)
**Low**	0.436	0.0504 (0.0482,0.0525)	9.61 (9.18,10.0)	1.41 (1.35,1.47)

Total productivity and healthcare costs averted from COVID-19 vaccines are presented as percentages of actual gross domestic product (GDP), per-person vaccinated in absolute terms, and per-person vaccinated as a percentage of gross domestic product per capita (GDPpc) as medians with 95% credible intervals. GDP reported is the total GDP worldwide and per income group for the 187 countries included in the analysis with available economic indicator data. Global and income-group estimates are reported as medians from simulated posterior draws. Income-group medians do not necessarily sum to the global median due to the nonadditivity of medians under uncertainty.

### Total value of benefits

In the CBA, the benefits, including VSLs and productivity and healthcare costs averted, summed to an estimated total of $64.3 trillion (95% CrI [62.2,67.4]), which is 82.7% (95% confidence interval [CI] [80.0,86.7]) of the global GDP. The CEA estimated a total of $6.43 trillion (95% CrI [6.21,6.73]) gained in value from COVID-19 vaccination. After discounting, these overall benefits are estimated at $4.82 trillion (95% CrI [4.66,5.04]). These CEA approach values correspond to 8.28% (95% CrI [7.99,8.66]) and 6.20% (95% CrI [5.99,6.49]), respectively. Global distributions of health and economic benefits can be seen in Fig 2 (undiscounted CEA approach in Fig A in S1 Appendix), and the relative proportion of each economic benefit generated in each analysis can be found in Fig 3.

**Fig 2:**
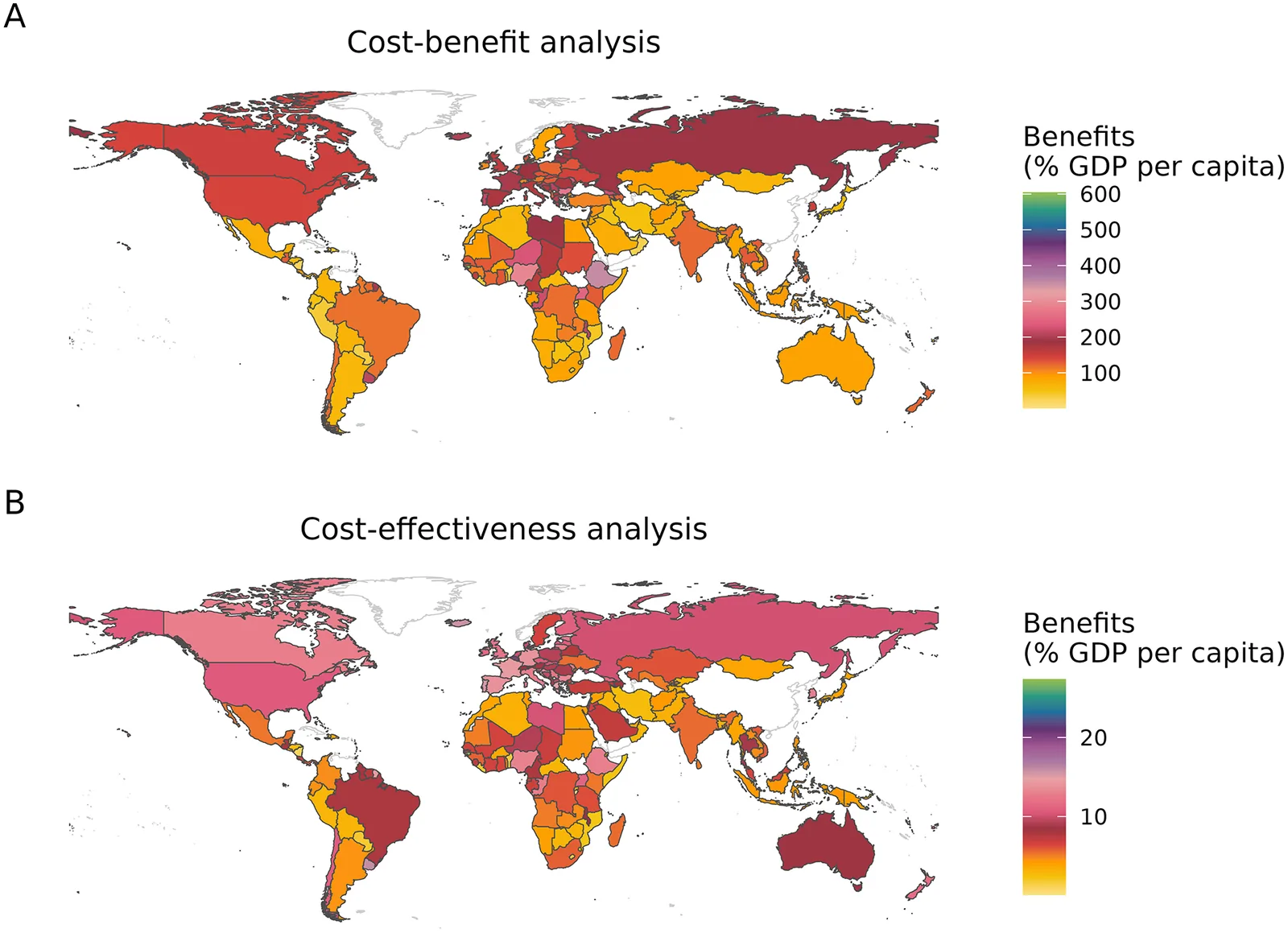
Global distribution of value of benefits from COVID-19 vaccination following cost-benefit analysis (CBA) and cost-effectiveness analysis (CEA) approaches. **(A)** The CBA includes value of statistical lives (VSLs) gained from averted mortality by COVID-19 vaccines, as well as productivity and healthcare costs averted. **(B)** The CEA includes the value of health gains (in discounted quality adjusted life years [QALYs]) from averted morbidity and mortality by COVID-19 vaccines, as well as productivity and healthcare costs averted. Results are expressed as a percentage of gross domestic product (GDP) per capita for each country. Made with Natural Earth. Free vector and raster map data @ naturalearthdata.com.

**Fig 3:**
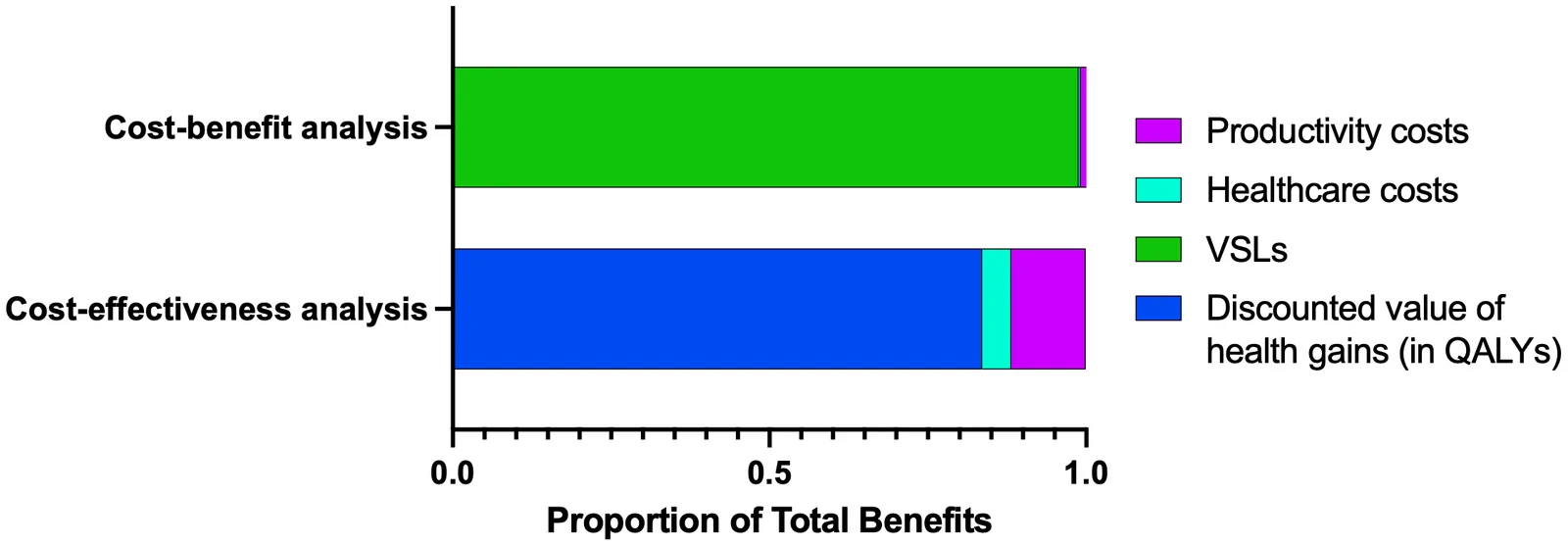
Relative contribution of benefit components to total economic benefits under the cost-effectiveness analysis and cost-benefit analysis. All contributions are expressed as proportions of the total estimated monetary value of benefits within each approach. Abbreviations: VSL, value of statistical life; QALY, quality-adjusted life year.

### ROI estimates and parameter uncertainty

Table 5 shows that all ROI estimates substantially exceed 1 (i.e., net positive returns) for COVID-19 vaccines globally for the first year of the vaccination programme, under both approaches. Based on the PSA, the CBA produced markedly higher ROI estimates (753 [95% uncertainty interval 309,1,559]) compared to the CEA (discounted: 60.3 [95% uncertainty interval 23.8,81.7]; undiscounted: 80.9 [95% uncertainty interval 29.8,111]). Across all approaches, the uncertainty intervals of the ROI far exceeded an ROI of 1 for the first year of the COVID-19 vaccination programme globally. Effects of parameter uncertainty on outcomes are found in Figs B–E in S1 Appendix. For VSLs, we found a positive correlation between VSL in the USA and averted deaths with increased VSLs (*p* < 0.001). We also found that cost-effectiveness thresholds were the most influential parameter driving QALYs (*p* < 0.001), according to the PRCC, showing a strong positive correlation with the value of health outcomes. Lastly, there was a strong correlation between the duration of friction periods (in HICs) and friction costs (*p* < 0.001), as well as between averted infections and friction costs (*p* < 0.001).

**Table 5:** Return-on-investment estimates for the first year of the COVID-19 vaccination programme globally following the base case and sensitivity analyses. Abbreviations: ROI, return-on-investment; VSL, value of statistical life; QALY, quality-adjusted life year; LMIC, lower-middle-income country; LIC, low-income country.

Base case analysis: Median (95% uncertainty interval)	ROI, $ (Median (95% uncertainty interval))
Cost-benefit analysis: VSLs, productivity and healthcare costs	809 (782,848)
Cost-effectiveness analysis: QALYs, productivity and healthcare costs (Discounted)	59.7 (57.7,62.5)
Cost-effectiveness analysis: QALYs, productivity and healthcare costs (Undiscounted)	80.0 (77.3,83.8)
**Probabilistic sensitivity analysis: Median (95% uncertainty interval)**	
Cost-benefit analysis: VSLs only	743 (299,1,549)
Cost-benefit analysis: VSLs, productivity and healthcare costs	753 (309,1,559)
Cost-benefit analysis: VSLYs, productivity and healthcare costs	327 (137,677)
Cost-effectiveness analysis: QALYs, productivity and healthcare costs (Discounted)	60.3 (23.8,81.7)
Cost-effectiveness analysis: QALYs, productivity and healthcare costs (Undiscounted)	80.9 (29.8,111)
**Additional sensitivity analyses: Median (95% uncertainty interval)**	
Cost-benefit analysis: VSLs only	799 (773,838)
Cost-benefit analysis: VSLYs, productivity and healthcare costs	352 (341,370)
Cost-benefit analysis: VSLYs, productivity and healthcare costs using uniform VSLY value globally	191 (184,196)
Cost-benefit analysis: VSLs, productivity costs and healthcare costs using income elasticity of 1.5 for LMICs and LICs	780 (753,818)

### Additional analyses

We also conducted a series of additional analyses whose findings did not reveal any changes in the interpretation of our ROI estimates. When limiting the CBA to only include VSLs (i.e., excluding healthcare and productivity costs), there is a slight decrease in the estimated returns per dollar invested. When using VSLY estimates in the CBA, a ROI well more than 1 was still observed. VSLY estimates globally and per income group can be found in Table D in S1 Appendix. Using a uniform VSLY value of $40,946.05 across countries, the ROI estimate from the CBA was still well in excess of 1. VSLYs gained per income group using a uniform VSLY value can be found in Table E in S1 Appendix and associated ROI estimates in Table 5. Additionally, when the income elasticity of LMICs and LICs was increased to 1.5, there was a slight decrease in the CBA ROI estimate.

## Discussion

This study represents the first global ROI analysis for COVID-19 vaccines. In the first year of the vaccination programme, these vaccines were found to have a ROI far greater than 1 in both the CBA and CEA. Globally, for every dollar invested in vaccine development and delivery, the estimated returns ranged from $59.7 to $809, depending on the valuation approach (i.e., CBA or CEA). Despite this net positive return globally, there were disparities in the health and economic benefits generated from COVID-19 vaccination across World Bank income groups. To our knowledge, this study provides the first economic evidence that the unprecedented, rapid investments made into COVID-19 vaccines paid off both in lives saved and long-term economic benefits. The overall findings were robust to changes in the approaches used (CBA or CEA, discounted or undiscounted) and in various sensitivity analyses.

In the CBA, averted mortality accounts for >99% of the high ROI estimate, reflecting the high value that individuals in society place on reductions in mortality risk. In the CEA, averted mortality and morbidity, quantified by QALYs gained, contribute most strongly to the estimated returns, highlighting the considerable intrinsic economic value of health gains. However, even the tangible economic benefits such as improved productivity exceeded the total global cost of vaccine development and delivery, indicating that investments into COVID-19 vaccines would have yielded net positive economic returns from a purely monetary perspective. This finding highlights the importance of vaccination for economic planning and development, in addition to public health and health systems. The two approaches value the benefits of vaccines differently, yet they both yield a net positive ROI, demonstrating that regardless of how the vaccines are valued (i.e., through individuals’ WTP for small reductions in mortality risk, as in the CBA, or through societal health opportunity cost thresholds, as in the CEA), the investment yielded substantial net returns. The benefits valued using VSLs and QALYs gained represent the economic valuation of health gains rather than financial returns or cash flows. While the CEA aimed to capture both quality and quantity of health gained, it yielded lower estimated benefits than the CBA because the estimates of health opportunity costs of healthcare spending at the margin used to value QALYs gained in monetary terms (Pichon-Riviere and colleagues 2023) [17], are lower than individuals’ WTP for marginal reductions in their mortality risk, as reflected by VSL-based valuations.

Our additional analyses demonstrate that varying key assumptions within the CBA do not meaningfully change the overall conclusions of the ROI estimate. First, when the CBA was restricted to VSLs (excluding productivity and healthcare costs), there were no marked differences in the ROI estimate. This reflects the dominance of VSL-based averted mortality valuations within the CBA, such that the inclusion or exclusion of productivity and healthcare costs has minimal effect on the ROI estimate. Second, when averted mortality benefits were valued using country-specific values of VSLYs, a reduction in the estimated ROI was observed, yet did not change the interpretation of our results. This finding was expected, as VSLY-based calculations capture the value of life years gained rather than deaths averted. Thus, an averted death in an older individual would yield a lower value as less life years were gained, in comparison to a younger individual. Given that COVID-19 disproportionately affected older populations, valuing the vaccines based on the number of life years gained estimated lower benefits in comparison to valuing them based on deaths averted, as is the case with VSL-based approaches. The value of VSLYs gained increased across income groups, reflecting both higher willingness to pay for mortality risk reductions and longer life expectancy in HICs, which together yield a greater monetary value of life years gained. To account for differences in the marginal value of money across income groups, we also applied a uniform global VSLY. While this approach produced similar trends in the absolute value of benefits, expressing the results on a per-person vaccinated basis as a percentage of GDPpc revealed that low-income countries experienced the largest relative gains. This finding highlights the comparatively greater economic benefits gained by vaccination in lower-income settings when assessed relative to available economic resources. Lastly, increasing the income elasticity to 1.5 for LMICs and LICs resulted in a modest reduction in ROI estimates. This decrease reflects that the value of VSLs in LMICs and LICs is lower than in HICs, given that those in lower-income settings must devote larger shares of their incomes to necessary and urgent expenses [44].

Our findings are consistent with other, more limited economic evaluations of COVID-19 vaccination, which also found that COVID-19 vaccines had high socioeconomic value on global, country, and regional levels [3,5,47–51]. The only other global analysis of COVID-19 vaccines’ value was conducted by Sevilla and colleagues [3]. This analysis had a much lower estimate of the number of lives saved by vaccination (4.1 million versus 14–20 million from the model we used) [1]. The difference between the two studies largely results from their reliance on reported COVID-19 case data (which is known to underestimate true burden in countries with weaker vital registration systems) [52]. Differences are also due to the regression approach used in Sevilla and colleagues, compared to the compartmental transmission model used by Watson and colleagues [1]. Despite the health impact difference, Sevilla and colleagues estimated that COVID-19 vaccines generated $5.2 trillion in value in health gains and GDP impact, similar to our equivalent CEA value [3]. The similarity between the two value figures is driven by the inclusion of vaccine impact on GDP in Sevilla and colleagues, which was estimated through regression independent of the health impact estimates.

Our findings are also consistent with other ROI analyses of adult respiratory vaccination. Recent studies have reported ROIs of ~19:1 for adult respiratory vaccination, including COVID-19, when broader societal benefits are monetized [53]. These studies adopted longer time horizons ranging from 5 years to a cohort’s lifetime, reflecting the sustained benefits accrued during endemic periods under routine immunization strategies. In contrast, our analysis focused on the first year of the COVID-19 vaccination programme during the period of peak pandemic transmission, morbidity, and mortality. Differences in ROI estimates across studies are likely due to the differences in time horizon and consequently epidemiological context, rather than inconsistencies in the underlying economic value of vaccination. Our study focused on the short-term peak pandemic effects to examine whether the extraordinary investment in vaccine development and delivery in 2020−2021 was justified, whereas these other studies evaluate the longer-term endemic vaccination strategies. Consistent with this interpretation, Chowdhury and colleagues (2025) report that COVID-19 continues to impose a major economic burden several years after the onset of the pandemic, and that COVID-19 vaccination continues to reduce morbidity and healthcare utilization, especially during the flu season [54]. Taken together, these findings indicate that COVID-19 vaccination delivers high economic benefits both during peak transmission and acute pandemic phases and under longer-term endemic vaccination strategies.

Our ROI of COVID-19 vaccination exceeds the already high ROIs of routine vaccination programmes in LMICs in Ozawa and colleagues [55]. That study reported an ROI of 22.2 per dollar invested in a range of vaccines (measles, rubella, rotavirus, pneumococcal, and HPV vaccines) under a cost-of-illness approach (similar to our CEA, but without monetizing QALYs), and 51.8 per dollar invested under a VSL approach (similar to our CBA approach). Differences in estimates can partly be attributable to methodological differences (lack of QALY monetization in the cost-of-illness approach used by Ozawa) and setting (Ozawa restricted to LMICs, where productivity costs are lower). However, our higher ROI values may also reflect the uniquely valuable contribution that COVID-19 vaccines made in the first year of a severe pandemic.

Despite our ROI estimates being greater than 1, the study reveals significant disparities in vaccine benefits across income groups. HICs gained the greatest economic benefits due to early access, higher coverage, and faster rollouts, especially before the Delta variant’s emergence in mid-2021 [1]. The older populations of HICs also contributed to greater vaccine benefit, as the per-person risk of severe outcomes was higher. Additionally, HICs gained higher economic value from averted COVID-19 mortality due to their high VSL estimates, which are based on gross national income per capita (GNIpc) values. In contrast, many LMICs had not yet begun vaccinating most of their populations during the Delta wave (mid-2021), and some LMICs (i.e., many countries in sub-Saharan Africa) had not even been able to achieve high vaccine coverage during the Omicron wave (late 2021). Hence, the value of vaccination was diminished as their populations had already received immunity through natural infection instead [1]. Earlier global access to COVID-19 vaccines would have led to substantial benefits, especially in LMICs, as reported by Barnsley and colleagues [51]. Additionally, HICs and UMICs could afford more efficacious vaccines (e.g., mRNA vaccines) which conferred greater health and economic benefits [1]. In contrast, LICs saw smaller total economic gains; however, their health benefits per-person vaccinated were relatively high as the low vaccine coverage targeted the most vulnerable. LICs also experienced the highest averted healthcare and productivity costs per-person vaccinated relative to GDPpc, emphasizing the profound economic benefits vaccines had on these countries despite their late introduction, and the importance of investments into vaccination programs in LICs to enhance health equity and global economic stability. In terms of uncertainty in public health estimates at the country-specific level, the intervals vary widely. However, they are proportionally narrow (95% intervals typically span ±10%–25% of the central estimate) for countries with large epidemics and high vaccine coverage that contribute most to global mortality, and wider primarily in settings with small epidemics or limited mortality data, without materially affecting regional or global conclusions.

Disparities between countries highlight the need for more equitable vaccine distribution strategies in global health crises, particularly in the early stages. While the COVAX facility aimed to create a fair allocation mechanism for COVID-19 vaccines, several challenges, including constrained and delayed vaccine supply to COVAX, prevented COVAX from reaching its target of 20% coverage in each country [1]. Had COVAX reached these goals, the benefits of vaccination would have been more equitably distributed as LMICs would have had access to vaccines sooner. The recently signed Pandemic Treaty promises LMICs access to vital technologies, including vaccines, in subsequent pandemics, although it lacks legally binding enforcement mechanisms and excludes participation by key countries such as the United States [9]. The most secure route may be to make vaccines a global public good during pandemics by removing restrictions on vaccine intellectual property and spreading vaccine production capacity to more countries [56,57]. Further actions to improve vaccine equity could include increased investment from HICs in vaccine development partnerships involving governments, universities, and companies and their counterparts in LMICs. These partnerships will increase capacity in LMICs to help ensure equity and maintenance of a worldwide collaboration network [58], with potential long-term economic benefits across all partners. Additionally, investments into increasing vaccine capacity and supply globally in advance of pandemics may enable a more equitable distribution of vaccines by limiting the incentive for vaccine nationalism [4]. By enhancing the global supply of vaccines and capacity to produce vaccines quickly in combination with limits on export bans and supply chain, this will enable countries to vaccinate their populations sooner and share their vaccine supplies with our countries, especially LMICs [4].

While this study provides important insights, it has limitations. Our results rely on previous analyses of COVID-19 vaccination impact, which is estimated based on a counterfactual modeling approach [1]. This approach is common practice in vaccine impact studies. However, it relies on simplifying assumptions around the counterfactual, such as assuming the level of public health and social restrictions, emergence of variants of concern, and human behavior and mixing remain the same as observed during the COVID-19 pandemic regardless of vaccine coverage. Compared with other global studies [3,48,59,60], Watson and colleagues’s estimates are notably higher due to methodological differences. Watson and colleagues model both direct and indirect effects of vaccination and relies on excess mortality estimates, rather than COVID-19 deaths alone. In contrast, other analyses use different modeling approaches with most using static models, but one using a similar dynamic model to Watson and colleagues. Of those that use static models, these models are unable to capture transmission effects and therefore underestimate vaccine benefits. They also do not consider excess mortality in their analyses. On the other hand, Moore and colleagues [59] used a dynamic model to evaluate how greater sharing of vaccines would have averted more deaths. However, Watson and colleagues report a higher number of deaths averted, likely due to the incorporation of vaccine prioritization amongst high-risk individuals and country-specific estimates of vaccine effectiveness. Watson and colleagues also include a greater number of countries.

Furthermore, this study only evaluates the ROI for the first year of COVID-19 vaccines. Although this was the most important year for vaccine impact as well as the year with the greatest vaccine development costs, incorporating additional benefits and costs that have occurred since would likely make the ROI estimates even more favorable. This includes booster doses (and continued rollout of primary courses) in response to the Omicron variant and its sub-lineages, which are likely to have also averted a substantial number of hospitalizations and deaths, as well as the health and economic impacts of long COVID-19. Other limitations include the lack of precise data and uncertainty estimates around health utility values and vaccine manufacturing and delivery costs. This study used QALY loss estimates per symptomatic infections, hospitalizations, and ICU admissions, which may not reflect the true QALY losses experienced by those who were infected or hospitalized with COVID-19, including the losses experienced by those affected by long COVID-19. Some infections may be more severe than others, and our use of one value for infection-related QALY losses may not capture the heterogeneity in the clinical outcomes of COVID-19 and the subsequent impacts on quality of life. Additionally, this study used one value for the cost of vaccine delivery for each country that was determined from a study in LMICs. Details on COVID-19 delivery costs, specifically in HICs, where the delivery costs are assumed to be higher than the value used, would enhance the ROI estimate’s precision and likely yield a lower ROI estimate for both approaches by increasing the costs of vaccine delivery. There has also been debate regarding the suitability of using VSL to evaluate COVID-19–related policies [61]. The certainty of our results is also limited by the lack of granular publicly available information on what investments made into vaccine research, development, and manufacturing were used for. Given this limitation, we took a maximal approach in estimating the costs to be cautious of this uncertainty yet still estimated a substantial net positive ROI.

One concern is that the VSL represents the marginal rate of substitution between wealth and mortality risk, but COVID-19 mortality risk in 2021 was so large that the rate is no longer marginal (meaning that people could not afford the wealth sacrifice needed to eliminate this risk, given commonly used VSL estimates e.g., approximately USD 10 million in the United States). In partial mitigation, it should be noted that vaccination coverage levels in 2021 fell far short of averting all mortality associated with COVID-19. Secondly, vaccination disproportionately averted deaths among older adults who might have a lower VSL due to their shorter remaining life expectancy. This would decrease the estimated ROI of vaccination, as demonstrated by our sensitivity analysis using VSLYs, although the ROI remains high even when using VSLYs. Thirdly, VSL reflects individuals’ preferences over risk, and risks that are perceived as more uncertain, salient, or dreadful, such as those associated with COVID-19, particularly early in the pandemic, may command higher willingness to pay for risk reductions. Under such circumstances, standard VSL values applied in our CBA may understate the benefits of vaccination. Taken together, these considerations highlight uncertainty around the appropriate valuation of COVID-19 mortality risk.

This study’s ROI estimates are likely conservative as health and economic benefits from the vaccines continued to accrue beyond the first year of this administration. We assumed that non-pharmaceutical interventions with adverse macroeconomic effects, such as lockdowns, remained unchanged after vaccination. In practice, however, most countries relaxed these restrictions as vaccination reduced infection risk. Such relaxations would have led to both benefits (reduced economic losses) and costs (increased infection risks). However, vaccination shifted the health-production possibilities frontier outward: for any given level of economic output, it reduced the associated health losses. By assuming that nonpharmaceutical interventions did not change, we effectively selected the point on this frontier that preserves the pre-vaccination level of economic output. This is unlikely to be optimal and therefore likely leads us to underestimate the benefits of vaccination. Despite these cautious assumptions, COVID-19 vaccines were and remain a sound global investment.

This study provides strong evidence to support investments made into COVID-19 vaccine development and delivery. The findings emphasize the critical economic value of vaccines in safeguarding public health and preserving global economic stability, which is important to establish at a time of reduced vaccine funding, particularly in LMICs, and heightened skepticism globally about vaccine value. Our findings highlight the value of investment in vaccine research and development before and during pandemics, as well as the importance of equitable vaccine access and distribution to reduce disparities across World Bank income groups. Ensuring vaccination program benefits are accessible to all populations, regardless of income level, is essential for enhancing global health equity and preparedness against future pandemics.

## Supporting information

S1 Appendix Methods. **Table A.** Vaccine research and development costs, including public funding, nonprofit funding, corporate investments, and manufacturing costs. **Table B.** Estimates of undiscounted and discounted life years and QALYs gained from averted COVID-19 morbidity and mortality by COVID-19 vaccination in the first year of the vaccination programme. **Table C.** Estimated infections and hospitalizations averted by COVID-19 vaccination in the first year of the vaccination programme. **Table D.** Estimated VSLYs gained by COVID-19 vaccination through averted mortality expressed in three ways using country-specific values. **Table E.** Estimated VSLYs gained by COVID-19 vaccination through averted mortality expressed in three ways using a global population-weighted mean value of a statistical life year. **Table F.** Estimated VSLs gained by COVID-19 vaccination through averted mortality expressed in three ways, varying income elasticity based on World Bank income groups. **Fig A.** Global distribution of undiscounted monetized benefits from COVID-19 vaccination in the cost-effectiveness analysis (CEA). **Fig B.** Tornado plots displaying partial rank correlation coefficients (PRCCs) of key parameters included in the probabilistic sensitivity analysis for VSLs. **Fig C.** Tornado plots displaying partial rank correlation coefficients (PRCCs) of key parameters included in the probabilistic sensitivity analysis for discounted value of health gains in QALYs. **Fig D.** Tornado plots displaying partial rank correlation coefficients (PRCCs) of key parameters included in the probabilistic sensitivity analysis for undiscounted value of health gains in QALYs. **Fig E.** Tornado plots displaying partial rank correlation coefficients (PRCCs) of key parameters included in the probabilistic sensitivity analysis for undiscounted monetized QALYs.
(DOCX)

S1 Checklist Consolidated Health Economic Evaluation Reporting Standards (CHEERS) checklist. (DOCX)

## Abbreviations

APAs: advanced purchase agreements
CBA: cost-benefit analysis
CEA: cost-effectiveness analysis
CHEERS: Consolidated Health Economic Evaluation Reporting Standards
CI: confidence interval
CrI: credible interval
COVAX: COVID-19 vaccines global access
FC: friction cost
GDP: gross domestic product
GDPpc: gross domestic product per capita
GNI: gross national income
GNIpc: gross national income per capita
HIC: high-income countries
LHS: Latin Hypercube Sampling
LICs: low-income countries
LMICs: lower-middle income countries
LY: life years
NIHR: National Institute for Health and Care Research
PRCCs: partial rank correlation coefficients
PSA: probabilistic sensitivity analysis
QALYs: quality-adjusted life years
ROI: return-on-investment
UMICs: upper-middle income countries
VSLs: values of a statistical life
VSLY: value of statistical life years
WHO: World Health Organization
WTP: willingness-to-pay.
