# Supplementary material for: Global return-on-investment of COVID-19 vaccines in the first year of the vaccination programme: A model-based economic evaluation
Source: PLoS Med. 2026 Nov 5;23(11):e1004772. doi: 10.1371/journal.pmed.1004772 (PMC13739145; doi:10.1371/journal.pmed.1004772)
Supplement: Supplementary file 1 [file pmed.1004772.s001.docx]

**S1 Appendix: Methods**

**Data collection**

To accurately assess the impact of COVID-19 vaccinations, our study relied on data sourced from a comprehensive global analysis of the impact of COVID-19 vaccination by Watson et al. This analysis provided estimates of the number of infections, hospitalizations, and deaths averted due to COVID-19 vaccination efforts worldwide for seventeen discrete age groups (0-5, 5-10, ..., 75-80, 80+) (Table 1). These public health outcomes (averted infections, hospitalizations and deaths) reported in Watson et al. were estimated by fitting a mechanistic transmission model of COVID-19 transmission and vaccination using novel fitting process-incorporating uncertainty in various epidemiological parameters. This model was fit to the time series of excess deaths recorded during the pandemic in each country. The authors estimated the public health outcomes that were averted due to vaccination by comparing the fitted model against a counterfactual scenario in which COVID-19 vaccinations were not administered, but all other public health interventions, such as NPIs, remained in place.

**Estimating health benefits**

For this health economic study, the public health outcomes from Watson et al. (2022) were used^1^. To capture the uncertainty in these outcomes, we drew 100 samples from the posterior distribution for each country analyzed. These public health outcomes include COVID-19 deaths, infections, and hospitalizations averted by the vaccines, aggregated by age group per country. The median and 95% credible intervals (CrI) for these outcomes within each country were estimated, with the 95% CrI calculated as the 95% central percentile from the 100 draws. Additionally, for aggregated estimates across different World Bank income groups, a sampling strategy where one draw was sampled from each country’s posterior distribution was employed. These individual draws from each country were subsequently summed to represent the total averted outcomes for that specific income group in a single simulation. This process was repeated 100 times to ensure that draws between countries were uncorrelated, with the resulting 100 draws used to calculate the median and 95% credible intervals for each health outcome.

When using the results from the Bayesian infectious disease model to calculate health economic outputs, the medians and 95% credible intervals were multiplied by fixed parameters. The fixed parameters include those in Table 1 of the main text.

**Averted infections, hospitalization and life years gained**

The public health outcomes from Watson et al.^1^ were used to report the infections and hospitalizations averted by COVID-19 vaccines.

Using the number of deaths averted by the vaccines and life expectancies, the number of LYs gained was estimated. The LYs gained were based on 5-year-band age-groups, beginning with 0-5 and ending with 80+. The undiscounted LYs gained for each age-group was calculated by multiplying each group’s average life expectancy by the number of deaths in that group. A discount rate of 3% was then applied to reflect the time preferences for health benefits to determine the discounted LYs gained.^2–4^ No female, male or health status differences were considered.

**Quality-adjusted life years gained**

A QALY is a health outcome measure that simultaneously captures changes in health-related quality of life, and changes in the duration of life due to premature deaths.^5^ This is calculated based on health state utilities on a scale in which a value of 1.0 is full health and a value of zero is death.^6^ The utilities assigned to health states are preference weights, based on time trade-offs where respondents compare between existing in a certain health state for a longer period of time before death, and a more preferred health state but for a shorter period of time.^7^ These health utilities are then multiplied by changes in life duration to determine the number of QALYs in a particular scenario and compared between scenarios to estimate the QALYs gained or lost by a particular intervention. The number of QALYs gained from COVID-19 vaccines was estimated from the averted QALY losses associated with COVID-19 infections, hospitalizations, and deaths (i.e. the difference in these outcomes between vaccination and non-vaccination scenarios). The number of QALYs gained from COVID-19 vaccination were based on the QALY losses associated with COVID-19 infections and hospitalizations and the number of cases averted. A single QALY losses estimate of 0.008 was applied to symptomatic COVID-19 infections. The number of symptomatic cases was found by multiplying the number of infections averted by one minus the proportion of asymptomatic cases for different age ranges. These proportions of asymptomatic cases were derived from Soh et al.^8^ Values of 0.0201 and 0.15 were applied to non-intensive care unit (ICU) and ICU hospitalizations. The number of hospitalizations that were admitted to ICU were derived from age-dependent probabilities of being admitted to the ICU from the transmission modelling by Watson et al. Equations 1 and 2 represent the calculations used to calculate the estimated QALYs gained.

**Equation 1: Estimating QALYs gained by COVID-19 vaccination from averted infections**

Infection QALYs gained = (Infections averted * (1 – probability of asymptomatic case)) * QALY loss for COVID-19 infections

**Equation 2: Estimating QALYs gained by COVID-19 vaccination from averted hospitalizations**

Hospitalization QALYs gained = (QALY loss for non-ICU hospitalizations * averted non-ICU hospitalizations) + (QALY loss for ICU hospitalizations * averted ICU hospitalizations)

The number of QALYs gained from averting a COVID-19 death was estimated as the number of LYs gained from averting the death, assuming perfect health in each year of life gained (QALY of 1.0). We estimated both undiscounted and discounted LYs. A discount rate of 3% was applied to the undiscounted LYs gained to estimate the undiscounted number of LYs gained. The calculation for undiscounted and discounted QALYs gained by COVID-19 vaccination is shown in Equation 3 below.

**Equation 3: Estimating QALYs gained by COVID-19 vaccination from averted deaths**

(Un)discounted QALYs gained from deaths averted = (Un)discounted life years lost

The total (un)discounted QALYs gained from vaccination was then estimated as the sum of the QALYs gained from averted hospitalizations, infections and deaths, with the latter quantity based on (un)discounted LYs.

**Valuing health benefits in monetary terms**

**Value of statistical life and value of statistical life years**

The ‘value of a statistical life’ (VSL) quantifies the willingness-to-pay (WTP) of individuals for small marginal changes in their mortality risk. VSLs are commonly derived from stated-preference and revealed preference studies of individuals’ willingness to pay (WTP) for marginal reduction in their current mortality risk and are frequently used to evaluate the benefits of interventions that reduce mortality risk^9^. Stated preference studies include surveys with carefully structured, hypothetical questions about an individual’s WTP for a lowered risk of death or disability while revealed preference studies include individuals’ observed behavior gathered from labor market data such as wage premiums for jobs at increased risk of morbidity or mortality for higher wages.^9^ To produce country-specific estimates of VSLs, we followed recommendations from Robinson et al. (2019).^10^ VSLs for each country were estimated using a USA reference value. The reference VSL was multiplied by the ratio of gross national income per capita (GNIPC) between the country of interest and the USA to the power of the income elasticity (Equation 5).^10^ GNIPC refers to the sum of the economic activities undertaken by citizens and firms of a country divided by the population.^2^ Income elasticity is the sensitivity of VSL changes in response to changes in income. An income elasticity of 1 was used, as recommended by Viscusi and Masterman (2018), given that it is a tractable value and their income elasticity analyses failed to reject the hypothesis that international income elasticity was equal to 1(i.e. a 10% increase in GNIPC results in a 10% increase in VSL).^11^ Additional sensitivity analyses were conducted to explore how an income elasticity of 1.5 for lower-income countries would affect the VSL estimates and overall ROI estimates.^10^

**Equation 4: Country-specific VSL estimates from USA reference value**

$${VSL}_{x}=\left( \frac{{VSL}_{USA}*{GNIPC}_{x}}{{GNIPC}_{USA}} \right)^{E}$$

The derived VSLs were used to calculate VSLYs, based on age group-dependent life expectancies and the distribution of the population amongst age groups. VSLYs represent an individual’s WTP for one-year gains in life expectancy.^12^ Following Robinson et al. (2019), the VSL estimate was divided by a population-weighted discounted life expectancy, where $l_{g}$ is the discounted LYs remaining per age group and $N_{g}$ is the number of people per age group, to get the value of a single year of statistical life (VSLY).^10^ To get the total VSLYs gained, the VSLY was multiplied by the number of undiscounted LYs gained (Equation 6). Using an undiscounted approach assumes that individuals do not discount future LYs in their states and revealed preferences when determining their WTP, and therefore value current and future LYs equally.

**Equation 5: VSLY estimates using population-weighted life expectancy, VSL estimates and deaths averted per age group**

$$VLY=\frac{VSL}{\left( \frac{\sum_{g} N_{g}l_{g}}{\sum_{g} Ng} \right)}$$

$$VSLY=VLY*l_{g}averted$$

**Value of health gains in QALYs**

To value the QALYs gained from COVID-19 vaccines, the QALYs gained were multiplied by cost-effectiveness thresholds. Most countries do not have locally-determined cost-effectiveness thresholds, so we used thresholds estimated from a study representing country-specific marginal productivity of health spending.^2^ These thresholds were obtained from Pichon-Riviere et al. (2023) for 174 countries, based on the economic capacities and opportunity costs of healthcare spending in each country income group. For example, Canada’s cost-effectiveness threshold in that study was 68% of its GDPpc while Uganda’s was 24%. The thresholds were then multiplied by the total QALYs gained from averted infections, hospitalizations, and deaths. This process was completed to obtain two estimates: undiscounted and discounted value of health gains in QALYs (Equation 6).

**Equation 6: Estimating total value of health gains in QALYs**

Total value of health gains in (un)discounted QALYs = (Equation 1 + Equation 2 + Equation 3) * % GDPpc

**Estimating economic benefits**

**Productivity costs**

COVID-19 morbidity and mortality caused significant productivity losses affecting the economies of countries globally. Productivity loss averted due to morbidity and mortality reductions is an important indirect benefit of COVID-19 vaccines.^14^ The costs of productivity losses averted by COVID-19 vaccines from COVID-19-related absenteeism for non-fatal cases and COVID-19 deaths were estimated using the friction cost (FC) approach. Through this approach, the indirect benefits of COVID-19 vaccines on productivity can be estimated. The FC method quantifies the costs incurred to the firm when workers are sick and cannot work or die and must be replaced. For unpaid workers, the opportunity cost approach was undertaken, in which the time spent on unpaid work is based on the value of spending this time in an alternative capacity (i.e. as a paid worker).^15^

**Non-fatal cases**

For non-fatal cases, the productivity costs incurred refer to the productivity loss during the time in which the worker was sick and unable to work. The productivity loss was valued by an individual’s average income per day multiplied by the duration of absenteeism. GDPpc was used as a proxy for average income and then divided by 365.25 to obtain the average income per day.^16^ This value was then multiped by the average absenteeism duration to determine COVID-19-related illness productivity losses. The absenteeism duration was based on the duration of COVID-19-related infections and hospitalizations. To obtain the productivity loss averted, the above value was multiplied by the number of cases averted (Equation 8). This process was completed for both hospitalizations and infections averted, using their respective durations, and then summed together to get the overall productivity costs averted for non-fatal cases.

**Equation 8: Productivity costs averted from averted COVID-19 morbidity by COVID-19 vaccines**

Productivity costs averted = (GDPpc / 365.25) * days absent * cases averted

**Fatal cases**

When someone dies from COVID-19, it can be assumed that there is a friction period where someone is not in that role, and therefore a cost is incurred to the firm.^14^ These productivity costs were determined by multiplying the friction period duration (in days) by the average daily income. GDPpc divided by 365.25 was used as a proxy for average daily income.^16^ To determine the costs averted for fatal cases, this above value was multiplied by the number of deaths averted (Equation 9).

**Equation 9: Productivity costs averted from averted COVID-19 mortality by COVID-19 vaccines**

Productivity costs averted = (GDPpc / 365.25) * friction period * deaths averted

The sum of the friction costs gained from non-fatal and fatal cases was calculated and used in the analysis.

**Healthcare costs**

Another economic benefit of COVID-19 vaccines was the attributable reductions in healthcare costs due to averted hospitalizations. For low- and middle-income countries (LMICs), healthcare costs per day for COVID-19-related hospitalizations were obtained from Torres-Rueda et al. (2021), which reported the data in US$2019. To inflate this to US$2021, the data was multiplied by a ratio of US$2021 GDP deflator index to the US$2019 GDP deflator index.^17^ To obtain the total healthcare cost per hospitalization, the cost per day was multiplied by the hospitalization duration. This cost was then multiplied by the number of hospitalizations averted to get the healthcare costs saved. For HICs, unit costs for hospitalization were obtained from WHO-CHOICE and reported in 2010 International Dollars (I$). The figures used were “Cost per inpatient bed day by hospital level (without drugs)” values for primary hospitals. To convert it to US$2021, given that I$1 reflects the comparable amount that US$1 could buy in the USA in the country and currency of interest, the data was multiplied by a ratio of 2021 to 2010 GDP deflator indexes to account for inflation^17^. Cost per hospitalization was then obtained by multiplying the cost by the hospitalization duration. To get the total costs averted, the cost per hospitalization was multiplied by the total number of hospitalizations averted (Equation 10).

**Equation 10: Estimating healthcare costs averted from averted COVID-19 hospitalizations by COVID-19 vaccines**

Healthcare costs = costs per hospitalization * duration * hospitalizations averted

**Probabilistic sensitivity analyses**

A probabilistic sensitivity analysis (PSA) was used to propagate parameter uncertainty to the ROI estimates. The PSA used Latin Hypercube Sampling (LHS) to generate 100 sets of values for parameters with reported uncertainties. The USA VSL value used in the VSL calculations was modeled using a gamma distribution, with shape and scale parameters derived from the reported mean and standard deviation. Percentages of GDP used to obtain opportunity cost thresholds were modeled using beta distributions, with shape parameters estimated by optimization between theoretical and empirical median and interquartile range values from the literature. QALY losses were also modeled using beta distributions, with shape parameters derived from reported mean values and 95% confidence intervals. Friction periods of HICs were modeled using a normal distribution with the reported mean and standard deviation from the literature. The PSA samples were randomly matched with the COVID-19 vaccine modelling samples and other economic outcomes, including healthcare costs, by calculating the median and 95% credible intervals of the draws to determine the 95% uncertainty intervals in the ROI estimates.

**Table A**. **Vaccine research and development costs, including public funding, non-profit funding, corporate investments and manufacturing costs.** Each row corresponds to one data point obtained; however, we had to group these data points into broader categories due to limited publicly available information on COVID-19 vaccine funding.

| **Cost category** | **Value**  **(millions)** | **Currency, year** | **Source** |
| --- | --- | --- | --- |
|  | | | |
| Public and non-profit funding for research, development, and production (includes some manufacturing costs) | 2100 | USD, 2020 | Wouters et al. (2021)^18^ |
|  | 2100 |  |  |
|  | 1700 |  |  |
|  | 1500 |  |  |
|  | 957 |  |  |
|  | 445 |  |  |
|  | 530 |  |  |
|  | 348 |  |  |
|  | 142 |  |  |
|  | 137 |  |  |
|  | 107 |  |  |
|  | 15 |  |  |
|  | 14 |  |  |
|  | 9 |  |  |
|  | 4 |  |  |
|  | 3 |  |  |
|  | 0.70 | USD, 2020 | COVID-19 Vaccine R&D Funding^19^ |
|  | 86.89 |  |  |
|  | 15.01 |  |  |
|  | 17.49 |  |  |
|  | 0.23 |  |  |
|  | 42.03 |  |  |
|  | 5.00 |  |  |
|  | 9.80 |  |  |
|  | 2.98 |  |  |
|  | 2.59 |  |  |
|  | 0.70 |  |  |
|  | 0.75 |  |  |
|  | 0.43 |  |  |
|  | 0.61 |  |  |
|  | 0.39 |  |  |
|  | 8.20 |  |  |
|  | 25.88 |  |  |
|  | 1.50 |  |  |
|  | 2.11 |  |  |
|  | 2.11 |  |  |
|  | 0.70 |  |  |
|  | 0.09 |  |  |
|  | 0.39 |  |  |
|  | 0.84 |  |  |
|  | 347.55 |  |  |
|  | 454.31 |  |  |
|  | 85.30 |  |  |
|  | 25.88 |  |  |
|  | 115.85 |  |  |
|  | 23.00 |  |  |
|  | 271.76 |  |  |
|  | 250.00 |  |  |
|  | 7.00 |  |  |
|  | 1.58 |  |  |
|  | 132.07 |  |  |
|  | 20.00 |  |  |
|  | 1.50 |  |  |
|  | 0.52 |  |  |
|  | 13.49 |  |  |
|  | 3.48 |  |  |
|  | 1.41 |  |  |
|  | 0.7 |  |  |
|  | 0.7 |  |  |
|  | 0.51 |  |  |
|  | 0.17620575 |  |  |
|  | 0.070483 |  |  |
|  | 1.41 |  |  |
|  | 237.00 |  |  |
|  | 162.19 |  |  |
|  | 3.88 |  |  |
|  | 3.02 |  |  |
|  | 27.00 |  |  |
|  | 4.90 |  |  |
|  | 8.30 |  |  |
|  | 5.70 |  |  |
|  | 2.36 |  |  |
|  | 4.00 |  |  |
|  | 5.79 |  |  |
|  | 0.30 |  |  |
|  | 0.41 |  |  |
|  | 10.00 |  |  |
|  | 10.81 |  |  |
|  | 7.05 |  |  |
|  | 57.93 |  |  |
|  | 2.20 |  |  |
|  | 0.94 |  |  |
|  | 1.41 |  |  |
|  | 0.75 |  |  |
|  | 0.75 |  |  |
|  | 0.54 |  |  |
|  | 0.56 |  |  |
|  | 0.45 |  |  |
|  | 0.75 |  |  |
|  | 2.47 |  |  |
|  | 150 |  |  |
|  | 6.90 |  |  |
|  | 29.12 |  |  |
|  | 17.26 |  |  |
|  | 11.48 |  |  |
|  | 8.25 |  |  |
|  | 9.01 |  |  |
|  | 11.26 |  |  |
|  | 4.63 |  |  |
|  | 1.16 |  |  |
|  | 2.15 |  |  |
|  | 8.00 |  |  |
|  | 1.30 |  |  |
|  | 115.85 |  |  |
|  | 7.05 |  |  |
|  | 5.29 |  |  |
|  | 86.89 |  |  |
|  | 0.49 |  |  |
|  | 0.28 |  |  |
|  | 1.06 |  |  |
|  | 0.14 |  |  |
|  | 0.34 |  |  |
|  | 2.32 |  |  |
|  | 1.00 |  |  |
|  | 11.90 |  |  |
|  | 23.94 |  |  |
|  | 3.16 |  |  |
|  | 38.03 |  |  |
|  | 9.91 |  |  |
|  | 1.41 |  |  |
|  | 1.74 |  |  |
|  | 144.96 |  |  |
|  | 21.95 |  |  |
|  | 2.90 |  |  |
|  | 2.85 |  |  |
|  | 1.95 |  |  |
|  | 0.02 |  |  |
|  | 0.10 |  |  |
|  | 0.12 |  |  |
|  | 21.55 | USD, 2021 | COVID-19 Vaccine R&D Funding^19^ |
|  | 139.02 |  |  |
|  | 4.8 |  |  |
|  | 14.2 |  |  |
|  | 33 |  |  |
|  | 32 |  |  |
|  | 132.4 |  |  |
|  | 1.06 |  |  |
|  | | | |
| Advanced purchasing agreements | 275 | USD, 2020 | COVID-19 Vaccine R&D Funding^19^ |
|  | 175 |  |  |
|  | 469.09 |  |  |
|  | 750 |  |  |
|  | 40 |  |  |
|  | 1060 |  |  |
|  | 250 |  |  |
|  | 120 |  |  |
|  | 4.3 |  |  |
|  | 12.47 |  |  |
|  | 942.65 |  |  |
|  | 1700 |  |  |
|  | 4500 |  |  |
|  | 2880 |  |  |
|  | 11340 |  |  |
|  | 352.5 |  |  |
|  | 84 |  |  |
|  | 5972.48 |  |  |
|  | 2790 |  |  |
|  | 858 |  |  |
|  | 2200 | USD, 2021 | COVID-19 Vaccine R&D Funding^19^ |
|  | | | |
| Manufacturing costs | 204 | USD, 2020 | COVID-19 Vaccine R&D Funding^19^ |
|  | 99 | USD, 2021 |  |
|  | 77 | USD, 2021 |  |
|  | | | |
| Corporate and private sector investments | 11000 | EUR, 2020 | European Parliament^20^ |
|  | 1.16 | USD, 2020 | COVID-19 Vaccine R&D Funding^19^ |
|  | 500 |  |  |
|  | 0.33 |  |  |
|  | 0.33 |  |  |
|  | 1.01 |  |  |
|  | 7.50 |  |  |
|  | 7.50 |  |  |

**Table B.** **Estimates of undiscounted and discounted life years and QALYs gained from averted COVID-19 morbidity and mortality by COVID-19 vaccination in the first year of the vaccination programme.** CrI, credible interval; LY, life year; QALY, quality-adjusted life year.

|  | **Vaccine coverage (%)** | **Total**  **(million)**  **(95% CrI)** | | **Per 1000 people vaccinated**  **(95% CrI)** | **Total**  **(million)**  **(95% CrI)** | | **Per 1000 people vaccinated**  **(95% CrI)** |
| --- | --- | --- | --- | --- | --- | --- | --- |
|  |  | **Undiscounted LYs gained** | | | **Discounted LYs gained** | | |
|  |  |  | | |  | | |
| **Worldwide** | 38.8 | 353  (341,362) | | 146  (141,150) | 252  (243,258) | | 104  (101,107) |
| **World Bank income group** | | | | | | | |
| **High** | 68.8 | 147  (142,155) | | 177  (171,187) | 104  (100,109) | | 125  (120,132) |
| **Upper-middle** | 50.1 | 75.1  (72.3,77.3) | | 132  (127,136) | 53.9  (51.9,55.4) | | 94.5  (91.0,97.2) |
| **Lower-middle** | 29.8 | 128  (120,133) | | 129  (121,134) | 91.7  (86.0,95.1) | | 92.6  (86.8,95.9) |
| **Low** | 3.57 | 2.70  (2.57,2.81) | | 118  (113,123) | 2.00  (1.89,2.06) | | 86.8  (82.6,90.2) |
|  | | **Undiscounted QALYs** | | | **Discounted QALYs** | | |
| **Worldwide** | 38.8 | 363 (351,373) | 150 (145,154) | | 262 (253,269) | 108 (105,111) | |
| **World Bank income group** | | | | | | | |
| **High** | 68.8 | 151 (146,159) | 182 (175,192) | | 108 (104,113) | 130 (125,136) | |
| **Upper-middle** | 50.1 | 77.2 (74.4,79.4) | 135 (131,139) | | 56.0 (53.9,57.5) | 98.2 (94.7,101) | |
| **Lower-middle** | 29.8 | 132 (124,137) | 133 (125,138) | | 95.9 (89.9,99.3) | 96.7 (90.7,100) | |
| **Low** | 3.57 | 2.82 (2.69,2.94) | 123 (118,129) | | 2.10 (20.0,2.19) | 92.0 (87.6,95.6) | |
| Total life years gained are presented as medians with 95% credible intervals. Values are also presented as the median total life years gained per 1000 people vaccinated (first or second dose) with 95% credible intervals. Total undiscounted and discounted QALYs gained from averted COVID-19 deaths, infections and hospitalizations from COVID-19 vaccines are presented as medians with 95% credible intervals, with values also presented as total QALYs gained per 1000 people vaccinated (first or second dose) as medians with 95% credible intervals. Discounted QALYs were calculated by applying a 3% discount rate to the life years gained from averted COVID-19 deaths. Vaccine coverage is the proportion of the population with a full dose in the modelled countries by December 8, 2021. | | | | | | | |

Table C. Estimated infections and hospitalizations averted by COVID-19 vaccination in the first year of the vaccination programme. CrI; credible interval.

|  | **Vaccine coverage (%)** | **Infections averted** | | **Hospitalizations averted** | |
| --- | --- | --- | --- | --- | --- |
|  |  | **Total (billion)**  **(95% CrI)** | **Per 1000 people vaccinated**  **(95% CrI)** | **Total (million)**  **(95% CrI)** | **Per 1000 people vaccinated**  **(95% CrI)** |
| **Worldwide** | 38.8 | 1.50  (1.45,1.54) | 622  (602,638) | 51.5  (50.0,52.7) | 21.3  (0.0207,0.0218) |
| **World Bank income group** | | | | | |
| **High** | 68.8 | 0.562  (0.539,0.582) | 676  (648,700) | 21.4  (20.5,22.1) | 25.8  (24.7,26.6) |
| **Upper-middle** | 50.1 | 0.303  (0.295,0.314) | 532  (518,551) | 11.4  (11.0,11.7) | 20.0  (19.2,20.6) |
| **Lower-middle** | 29.8 | 0.619  (0.584,0.643) | 624  (589,648) | 18.4  (17.0,19.0) | 18.5  (17.1,19.1) |
| **Low** | 3.57 | 0.0198  (0.0190,0.0208) | 867  (833,909) | 0.472  (0.450,0.493) | 20.7  (19.7,21.6) |
| Total infections and hospitalizations averted from COVID-19 vaccines are presented as medians with 95% credible intervals, with values also presented as total infections or hospitalizations averted per 1000 people vaccinated (first or second dose) as medians with 95% credible intervals. Hospitalizations are considered as ‘potential’ hospitalizations, representing the incidence of averted severe cases that would have required hospitalization; whether hospitalization occurred or not is dependent on healthcare capacity. | | | | | |

Table D. Estimated value of statistical life years (VSLYs) gained by COVID-19 vaccination through averted mortality expressed in three ways using country-specific values. CrI, credible interval; GDP, gross domestic product; GDPpc, gross domestic product per capita.

|  | **GDP**  **Trillion ($))** | **VSLYs gained** | | |
| --- | --- | --- | --- | --- |
|  |  | **Total**  **(% of GDP)** **(95% CrI)** | **Per-person vaccinated**  **(Thousands**  **($))** **(95% CrI)** | **Per-person vaccinated (% of GDPpc)** **(95% CrI)** |
| **Worldwide** | 77.8 | 35.0  (33.9,36.8) | 11.3  (10.9,11.8) | 91.6  (88.6,96.2) |
| **World Bank income group** | | | | |
| **High** | 58.9 | 40.9  (39.4,43.1) | 29.0  (27.9,30.5) | 59.4  (57.2,62.6) |
| **Upper-middle** | 9.70 | 21.8  (20.9,22.4) | 3.71  (3.56,3.81) | 43.5  (41.8,44.7) |
| **Lower-middle** | 8.74 | 12.2  (11.5,12.8) | 1.08  (1.02,1.13) | 41.1  (38.7,42.8) |
| **Low** | 0.436 | 1.28  (1.21,1.33) | 0.244  (0.231,0.254) | 35.7  (33.9,37.2) |
| Total VSLYs gained from averted COVID-19 deaths are presented as percentages of GDP, per-person vaccinated in absolute terms, and per-person vaccinated as a percentage of GDPpc as medians with 95% credible intervals. GDP reported is the reported total GDP worldwide and per income group for the 187 countries included in the analysis with available economic indicator data. Global and income-group estimates are reported as medians from simulated posterior draws. Income-group medians do not necessarily sum to the global median due to the non-additivity of medians under uncertainty. | | | | |

Table E. Estimated value of statistical life years (VSLYs) gained by COVID-19 vaccination through averted mortality expressed in three ways using a global population-weighted mean value of a statistical life year. CrI, credible interval; GDP, gross domestic product; GDPpc, gross domestic product per capita.

|  | **GDP**  **Trillion ($))** | **VSLYs gained** | | |
| --- | --- | --- | --- | --- |
|  |  | **Total**  **(% of GDP)** **(95% CrI)** | **Per-person vaccinated**  **(Thousands**  **($))** **(95% CrI)** | **Per-person vaccinated (% of GDPpc)** **(95% CrI)** |
| **Worldwide** | 77.8 | 18.6 (18.0,19.1) | 5.99 (5.78,6.14) | 48.6 (47.0,49.9) |
| **World Bank income group** | | | | |
| **High** | 58.9 | 10.3 (9.88,10.8) | 7.26 (7.00,7.66) | 14.9 (14.4,15.7) |
| **Upper-middle** | 9.70 | 31.7 (30.5,32.6) | 5.40 (5.20,5.56) | 63.3 (61.0,65.2) |
| **Lower-middle** | 8.74 | 59.9 (56.2,62.1) | 5.29 (4.96,5.47) | 201 (189,208) |
| **Low** | 0.436 | 25.4 (24.2,26.5) | 4.80 (4.61,5.04) | 710 (677,740) |
| Total VSLYs gained from averted COVID-19 deaths are presented as percentages of GDP, per-person vaccinated in absolute terms, and per-person vaccinated as a percentage of GDPpc as medians with 95% credible intervals. GDP reported is the reported total GDP worldwide and per income group for the 187 countries included in the analysis with available economic indicator data. Global and income-group estimates are reported as medians from simulated posterior draws. Income-group medians do not necessarily sum to the global median due to the non-additivity of medians under uncertainty. | | | | |

Table F. Estimated values of a statistical life (VSLs) gained by COVID-19 vaccination through averted mortality expressed in three ways varying income elasticity based on World Bank income groups. CrI, credible interval; GDP, gross domestic product; GDPpc, gross domestic product per capita.

|  | **GDP**  **Trillion ($))** | **VSLs gained** | | |
| --- | --- | --- | --- | --- |
|  |  | **Total**  **(% of GDP)** **(95% CrI)** | **Per-person vaccinated**  **(Thousands**  **($))** **(95% CrI)** | **Per-person vaccinated (% of GDPpc)** **(95% CrI)** |
| **Worldwide** | 77.8 | 78.7 (76.1,82.6) | 25.3 (24.5,26.6) | 206 (199,216) |
| **World Bank income group** | | | | |
| **High** | 58.9 | 93.7 (90.3,98.9) | 66.4 (63.9,69.9) | 136 (131,143) |
| **Upper-middle** | 9.70 | 56.3 (54.3,57.9) | 9.59 (9.24,9.85) | 112 (108,116) |
| **Lower-middle** | 8.74 | 6.53 (6.13,6.79) | 0.575 (0.540,0.599) | 21.9 (20.6,22.8) |
| **Low** | 0.436 | 0.440 (0.414,0.460) | 0.0839 (0.0789,0.0876) | 12.3 (11.6,12.9) |
| Total VSLs gained from averted COVID-19 deaths are presented as percentages of GDP, per-person vaccinated in absolute terms, and per-person vaccinated as a percentage of GDPpc as medians with 95% credible intervals. GDP reported is the reported total GDP worldwide and per income group for the 187 countries included in the analysis with available economic indicator data. Global and income-group estimates are reported as medians from simulated posterior draws. Income-group medians do not necessarily sum to the global median due to the non-additivity of medians under uncertainty. | | | | |

**Fig A**. **Global distribution of undiscounted monetized benefits from COVID-19 vaccination in the cost-effectiveness analysis (CEA).** The CEA includes the value of health gains (in discounted quality adjusted life years [QALYs]) from averted morbidity and mortality by COVID-19 vaccines, as well as productivity and healthcare costs averted. Results are expressed as a percentage of gross-domestic-product (GDP) per capita for each country. Made with Natural Earth. Free vector and raster map data @ naturalearthdata.com.

**Fig B.** **Tornado plots displaying partial rank correlation coefficients (PRCCs) of key parameters included in the probabilistic sensitivity analysis for VSLs.** Higher absolute PRCCs indicate greater sensitivity of the outcome to the uncertainty in the corresponding parameter. VSL; value of statistical life.

**Fig C.** **Tornado plots displaying partial rank correlation coefficients (PRCCs) of key parameters included in the probabilistic sensitivity analysis for discounted value of health gains in QALYs.** Higher absolute PRCCs indicate greater sensitivity of the outcome to the uncertainty in the corresponding parameter. QALY; quality-adjusted life year; GDP; gross-domestic product, HIC; high-income country; UMIC, upper-middle income country; LMIC; lower-middle income country, LIC; low-income country.

**Fig D.** **Tornado plots displaying partial rank correlation coefficients (PRCCs) of key parameters included in the probabilistic sensitivity analysis for undiscounted value of health gains in QALYs.** Higher absolute PRCCs indicate greater sensitivity of the outcome to the uncertainty in the corresponding parameter. QALY; quality-adjusted life year; GDP; gross-domestic product, HIC; high-income country; UMIC, upper-middle income country; LMIC; lower-middle income country, LIC; low-income country.

**Fig E.** **Tornado plots displaying partial rank correlation coefficients (PRCCs) of key parameters included in the probabilistic sensitivity analysis for undiscounted monetized QALYs.** Higher absolute PRCCs indicate greater sensitivity of the outcome to the uncertainty in the corresponding parameter. HIC; high-income country.

**References**

1.	Watson OJ, Barnsley G, Toor J, Hogan AB, Winskill P, Ghani AC. Global impact of the first year of COVID-19 vaccination: a mathematical modelling study. *Lancet Infect Dis*. 2022;22(9):1293-1302. doi:10.1016/S1473-3099(22)00320-6

2.	Guinness L, Wiseman V. *Introduction to Health Economics*. McGraw-Hill Education (UK); 2011.

3.	Haacker M, Hallett TB, Atun R. On discount rates for economic evaluations in global health. *Health Policy Plan*. 2020;35(1):107-114. doi:10.1093/heapol/czz127

4.	Sanders GD, Neumann PJ, Basu A, et al. Recommendations for Conduct, Methodological Practices, and Reporting of Cost-effectiveness Analyses: Second Panel on Cost-Effectiveness in Health and Medicine. *JAMA*. 2016;316(10):1093. doi:10.1001/jama.2016.12195

5.	Drummond M. *Methods for the Economic Evaluation of Health Care*. Oxford University Press; 2015.

6.	Prieto L, Sacristán JA. Problems and solutions in calculating quality-adjusted life years (QALYs). *Health Qual Life Outcomes*. 2003;1:80. doi:10.1186/1477-7525-1-80

7.	Whitehead SJ, Ali S. Health outcomes in economic evaluation: the QALY and utilities. *Br Med Bull*. 2010;96(1):5-21. doi:10.1093/bmb/ldq033

8.	Sah P, Fitzpatrick MC, Zimmer CF, et al. Asymptomatic SARS-CoV-2 infection: A systematic review and meta-analysis. *Proc Natl Acad Sci U S A*. 2021;118(34):e2109229118. doi:10.1073/pnas.2109229118

9.	Laxminarayan R, Jamison DT, Krupnick AJ, Norheim OF. Valuing vaccines using value of statistical life measures. *Vaccine*. 2014;32(39):5065-5070. doi:10.1016/j.vaccine.2014.07.003

10.	Robinson LA, Hammitt JK, Cecchini M, et al. Reference Case Guidelines for Benefit-Cost Analysis in Global Health and Development. Preprint posted online May 1, 2019:4015886. doi:10.2139/ssrn.4015886

11.	Masterman CJ, Viscusi WK. The Income Elasticity of Global Values of a Statistical Life: Stated Preference Evidence. *J Benefit-Cost Anal*. 2018;9(3):407-434. doi:10.1017/bca.2018.20

12.	Robinson LA, Sullivan R, Shogren JF. Do the Benefits of COVID‐19 Policies Exceed the Costs? Exploring Uncertainties in the Age–VSL Relationship. *Risk Anal*. 2021;41(5):761-770. doi:10.1111/risa.13561

13.	Colombo M. For a Few Neurons More: Tractability and Neurally Informed Economic Modelling. *Br J Philos Sci*. 2015;66(4):713-736. doi:10.1093/bjps/axu013

14.	Park M, Jit M, Wu JT. Cost-benefit analysis of vaccination: a comparative analysis of eight approaches for valuing changes to mortality and morbidity risks. *BMC Med*. 2018;16(1):139. doi:10.1186/s12916-018-1130-7

15.	Jiang S, Wang Y, Si L, et al. Incorporating productivity loss in health economic evaluations: a review of guidelines and practices worldwide for research agenda in China. *BMJ Glob Health*. 2022;7(8):e009777. doi:10.1136/bmjgh-2022-009777

16.	Krol M, Brouwer W. How to Estimate Productivity Costs in Economic Evaluations. *PharmacoEconomics*. 2014;32(4):335-344. doi:10.1007/s40273-014-0132-3

17.	Turner HC, Lauer JA, Tran BX, Teerawattananon Y, Jit M. Adjusting for Inflation and Currency Changes Within Health Economic Studies. *Value Health*. 2019;22(9):1026-1032. doi:10.1016/j.jval.2019.03.021

18.	Wouters OJ, Shadlen KC, Salcher-Konrad M, et al. Challenges in ensuring global access to COVID-19 vaccines: production, affordability, allocation, and deployment. *Lancet Lond Engl*. 2021;397(10278):1023-1034. doi:10.1016/S0140-6736(21)00306-8

19.	COVID-19 Vaccine R&D Funding. Knowledge Portal. Accessed March 5, 2026. https://www.knowledgeportalia.org/covid-19-vaccine-r-d-funding

20.	European Parliament, Directorate-General for Internal Policies of the Union, Florio M, Gamba S, Pancotti C. Mapping of long-term public and private investments in the development of COVID-19 vaccines. European Parliament, 2023. https://doi.org/10.2861/481388 (accessed March 5, 2026).

**LEGEND**

S1 Appendix: Methods

Table A. Vaccine research and development costs, including public funding, non-profit funding, corporate investments and manufacturing costs.

Table B. Estimates of undiscounted and discounted life years and QALYs gained from averted COVID-19 morbidity and mortality by COVID-19 vaccination in the first year of the vaccination programme.

Table C. Estimated infections and hospitalizations averted by COVID-19 vaccination in the first year of the vaccination programme.

Table D. Estimated VSLYs gained by COVID-19 vaccination through averted mortality expressed in three ways using country-specific values.

Table E. Estimated VSLYs gained by COVID-19 vaccination through averted mortality expressed in three ways using a global population-weighted mean value of a statistical life year.

Table F. Estimated VSLs gained by COVID-19 vaccination through averted mortality expressed in three ways varying income elasticity based on World Bank income groups.

Fig A. Global distribution of undiscounted monetized benefits from COVID-19 vaccination in the cost-effectiveness analysis (CEA).

Fig B. Tornado plots displaying partial rank correlation coefficients (PRCCs) of key parameters included in the probabilistic sensitivity analysis for VSLs.

Fig C. Tornado plots displaying partial rank correlation coefficients (PRCCs) of key parameters included in the probabilistic sensitivity analysis for discounted value of health gains in QALYs.

Fig D. Tornado plots displaying partial rank correlation coefficients (PRCCs) of key parameters included in the probabilistic sensitivity analysis for undiscounted value of health gains in QALYs.

Fig E. Tornado plots displaying partial rank correlation coefficients (PRCCs) of key parameters included in the probabilistic sensitivity analysis for undiscounted monetized QALYs.
