# Supplementary material for: Global return-on-investment of COVID-19 vaccines in the first year of the vaccination programme: A model-based economic evaluation
Source: PLoS Med. 2026 Nov 5;23(11):e1004772. doi: 10.1371/journal.pmed.1004772 (PMC13739145; doi:10.1371/journal.pmed.1004772)
Supplement: Supplementary file 2 [file pmed.1004772.s002.docx]

# S1 Checklist: CHEERS 2022 Checklist

| **Topic** | **No.** | **Item** | **Location where item is reported** |
| --- | --- | --- | --- |
| **Title** |  |  |  |
|  | 1 | Identify the study as an economic evaluation and specify the interventions being compared. | Title, Page 1 (The global return-on-investment of COVID-19 vaccines in the first year of the vaccination programme) |
| **Abstract** |  |  |  |
|  | 2 | Provide a structured summary that highlights context, key methods, results, and alternative analyses. | Abstract, Page 2, Background: COVID-19 vaccines played a critical role in reducing global health burden during the COVID-19 pandemic, but their rapid development required extraordinary effort. This study aims to evaluate their global return-on-investment (ROI) during the first year of the vaccination programme (8 December 2020-8 December 2021) to provide insights into the economic benefits relative to the costs of vaccine development, manufacturing, and delivery. Methods: Public health outcomes of COVID-19 vaccination, including life years and QALYs gained from averted death, infections, and hospitalizations, were estimated. Health benefits were valued using two approaches: i) cost-benefit analysis and ii) cost-effectiveness analysis. These benefits were compared to the costs of vaccine development, manufacturing, and delivery to estimate the ROI under both approaches. Probabilistic sensitivity analyses were conducted to assess the impact of parameter uncertainty on ROI estimates. Limitations include reliance on previous analyses of COVID-19 vaccination impact and a time horizon limited to the first year of COVID-19 vaccination. Findings: Vaccine development and delivery cost $79.4 billion, but the health and economic benefits of vaccination were valued between $6.43–$64.3 trillion. High-income countries saw the greatest health benefits per-person vaccinated, and low-income countries gained the greatest non-health benefits per-person vaccinated, when expressed as a proportion of GDP per capita. This estimated ROIs of $59.6 –$809 per dollar invested in the base case analysis. ROIs ranged between $60.2 – $753 across probabilistic sensitivity analyses for both approaches. Conclusions: Rapid COVID-19 vaccine development and delivery were an excellent investment. Vaccine development and equitable vaccine distribution globally should be prioritized before and during global health crises. |
| **Introduction** |  |  |  |
| **Background and objectives** | 3 | Give the context for the study, the study question, and its practical relevance for decision making in policy or practice. | Background, Page 5 (Paragraph 2) - Page 7 (Paragraph 1) Although numerous economic evaluations of COVID-19 vaccines have informed vaccine procurement and deployment at the country-level, none have assessed the value for money of vaccine development itself, rather than just vaccine procurement. A systematic review of economic evaluations for COVID-19 vaccines conducted in 20232 identified 25 full economic evaluation studies, including cost-minimization, cost-benefit, cost-effectiveness, and cost-utility analyses. However, they all compared country-level benefits to the cost of the national programme rather than the cost of vaccine development.2 While a recent study looked at COVID-19 vaccination’s impact on global gross domestic product based on regression analysis, it did not look at the return-on-investment (ROI) of vaccine development itself.3 Additionally, the growing literature of economic evaluations on COVID-19 vaccines has primarily assessed the value of vaccines on the country level, limiting their analyses to national procurement and delivery costs. These analyses do not capture the full scale of investments that enabled vaccine availability in the first place. COVID-19 vaccines development was unique, because the vaccines’ rapid development required substantial, coordinated public investment across countries, including direct funding of research and development and Advanced Purchase Agreements (APAs) that de-risked private-sector production. These investments were made upstream of national vaccination programmes and generated global health and economic benefits that extend beyond any single country’s health system.4 As governments and global institutions consider how best to protect global populations in future pandemics, understanding the global economic returns to these upstream investments is critical. ROI analyses evaluate the economic benefits of an intervention relative to its costs, often using a cost-benefit approach that monetizes health and non-health impacts.5 Despite the complexity of conducting ROI analyses for vaccine impact, including selecting the appropriate methodology, ROI analyses are vital for demonstrating the health and economic value of vaccines and building strong investment arguments.6 Estimating the global ROI of COVID-19 vaccine development and delivery is therefore relevant and necessary to pandemic preparedness planning, including decisions about whether similar public investments should be completed for future global health emergencies. Quantifying the ROI of COVID-19 vaccine development may also inform ongoing debates regarding vaccine pricing. During the pandemic, concerns were raised that vaccines developed with public funding were subsequently sold back to governments at higher prices and at variable prices between governments, raising questions about equity, affordability, and corporate profits.7,8 By estimating the total economic and health surplus generated by COVID-19 vaccines in monetary value relative to their development and distribution costs, conducting a ROI analysis may provide evidence that the health and economic benefits provided by the vaccines were worth their cost. Recently, there have been promising steps toward greater equity in pandemic vaccine distribution, such as the signing of the World Health Organization’s Pandemic Treaty.9 However, some funding streams for pandemic vaccine research have also been cut.10 In the event of future pandemics, a clearer understanding of the ROI from COVID-19 vaccines could play a critical role in guiding decisions around comparable investments in rapid vaccine development. Furthermore, COVID-19 vaccine benefits are unequally distributed across countries due to access inequalities, and the economic implications of this inequity have not been fully enumerated. To address these evidence gaps, we aimed to evaluate the global ROI for COVID-19 vaccines over the first year of their use (8 December 2020–8 December 2021), when the vaccines had the greatest impact due to limited pre-existing immunity, by comparing the vaccines’ benefits to their development and distribution costs. We also aimed to estimate the wider societal benefits of COVID-19 vaccination, including reduced mortality and morbidity, as well as improved productivity, and to compare these benefits generated across World Bank income groups. |
| **Methods** |  |  |  |
| **Health economic analysis plan** | 4 | Indicate whether a health economic analysis plan was developed and where available. | Method, Page 7 (Paragraph 1) The ROI of COVID-19 vaccination over the first year of the vaccination programme globally was determined by estimating the value of its public health outcomes and then comparing them to the investments made into COVID-19 vaccine development and delivery using two standard approaches: a cost-benefit analysis (CBA) and a cost-effectiveness analysis (CEA). |
| **Study population** | 5 | Describe characteristics of the study population (such as age range, demographics, socioeconomic, or clinical characteristics). | Methods, Page 8 (Paragraph 2) This study used an age-structured, country-specific transmission model of SARS-CoV-2 to estimate the number of infections, hospitalizations, and deaths averted by COVID-19 vaccination during the first year of global rollout (8 December 2020 to 8 December 2021) for seventeen discrete age groups (0-5, 5-10, ..., 75-80, 80+) (Table 1). |
| **Setting and location** | 6 | Provide relevant contextual information that may influence findings. | Methods, Page 8 to Page 9, The model is a deterministic compartmental transmission model stratified by age and vaccination status, which models SARS-CoV-2 infection, progression to severe disease, hospitalization, and death. The model accounts for age-specific susceptibility, severity, and infection fatality risk and incorporates country-specific demographic structures, contact patterns, and vaccination roll-out. The model was fit to excess all-cause mortality18 to mitigate biases arising from under-ascertainment and differences in COVID-19 death reporting systems globally. Model parameters were inferred separately for each country. The fitting framework explicitly incorporated uncertainty in key epidemiological parameters, including transmission intensity, infection fatality ratios, and vaccine effectiveness against severe outcomes. To estimate vaccine impact, the fitted model was simulated under two scenarios: (i) the observed vaccination rollout, and (ii) a counterfactual scenario in which no COVID-19 vaccines were administered, while all other factors, including non-pharmaceutical interventions, behavioral changes, and variant emergence, were assumed to remain as observed. The difference between these scenarios yielded estimates of infections, hospitalizations, and deaths averted by vaccination. Uncertainty in epidemiological outcomes was propagated by drawing 100 posterior samples per country from the fitted model. These samples were used directly as inputs to the economic evaluation, ensuring that uncertainty from the transmission model was carried through to all health and ROI estimates. Further methodological details are provided in Appendix B and in Watson et al.1 Given that non-pharmaceutical interventions, behavioral changes, and variant emergence were assumed to remain as historically observed, the benefits of COVID-19 vaccination considered in this study are limited to those related to the direct reduction in COVID-19 morbidity and mortality, and do not capture costs and benefits arising from associated policy or behavioral changes enabled by vaccination (such as relaxation of lockdowns). |
| **Comparators** | 7 | Describe the interventions or strategies being compared and why chosen. | Method, Page 12, Paragraphs 2 and 3, To determine the ROI, the value of the benefits were compared to the costs using the following general equation: ROI=(benefits–costs)/costs.26 Two ROI estimates were obtained: i) for the CBA, ROI where the benefits include VSLs gained and productivity and healthcare costs averted, and ii) for the CEA, ROI where the benefits include the value of the QALYs gained and productivity and healthcare costs averted. |
| **Perspective** | 8 | State the perspective(s) adopted by the study and why chosen. | Methods, Page 9 (Paragraph 3) The study follows a societal perspective at a global level. Only true economic costs and benefits were considered, which excludes fiscal transfers between entities such as government vaccine purchases from manufacturers or intermediaries, including COVID-19 Vaccines Global Access (COVAX).19 Under this perspective, all costs and benefits associated with the vaccines are included, regardless of who incurred them. |
| **Time horizon** | 9 | State the time horizon for the study and why appropriate. | Methods, Page 9 (Paragraph 1), The time horizon includes COVID-19 vaccine development and delivery costs between 2020-2021, and the health and economic benefits generated by the vaccines delivered between 8 December 2020 – 8 December 2021 (including future life years lost from deaths in 2020–2021). |
| **Discount rate** | 10 | Report the discount rate(s) and reason chosen. | Methods, Page 10 (Paragraph 2), LYs gained were determined by multiplying age group-specific average life expectancies for each country by the number of deaths averted, with a 3% discount rate applied to reflect time preferences for current health benefits. |
| **Selection of outcomes** | 11 | Describe what outcomes were used as the measure(s) of benefit(s) and harm(s). | Methods, Page 12 and Figure 1. Two ROI estimates were obtained: i) for the CBA, ROI where the benefits include VSLs gained and productivity and healthcare costs averted, and ii) for the CEA, ROI where the benefits include the value of the QALYs gained and productivity and healthcare costs averted. |
| **Measurement of outcomes** | 12 | Describe how outcomes used to capture benefit(s) and harm(s) were measured. | Methods, Pages 10-11. The health benefits generated from COVID-19 vaccines were evaluated in terms of deaths averted and life years (LY) and QALYs gained. LYs gained were determined by multiplying age group-specific average life expectancies for each country by the number of deaths averted, with a 3% discount rate applied to reflect time preferences for current health benefits. Undiscounted and discounted QALYs gained were estimated by combining averted QALY losses from infections, hospitalizations, and deaths. QALY loss estimated ranged between 0.15 and 0.008 per symptomatic case (including hospitalizations and intensive care unit admissions) and perfect health (QALY of 1.0) was assumed for averted deaths. Further details are in Appendix B. For the CBA, deaths averted were valued using country-specific VSLs, which represents the marginal rate of substitution between income and mortality risk, derived from individuals’ WTP for small reductions in mortality risk. Following Robinson et al.,21 VSLs were derived by adjusting a US reference value using country-specific gross national income (GNI) per capita and an income elasticity of 1. For the CEA, undiscounted and discounted QALYs gained were valued by applying opportunity cost thresholds, which are based on income-group dependent percentages of gross domestic product (GDP) per capita and represent the marginal productivity of health spending. Further details are in Appendix B. Economic benefits generated during the first year of the COVID-19 vaccination programme were assessed from a societal perspective in both the CBA and CEA, by estimating both averted productivity losses and healthcare costs. Productivity costs averted due to reduced COVID-19 related absenteeism and deaths were calculated using the friction cost (FC) approach. This approach was chosen over the human capital approach to be conservative in estimating vaccine benefits; the FC approach assumes that sick or deceased workers are replaceable and only considers productivity costs incurred during the period when the position is unfilled (friction period). These costs represent the output losses borne by employers during the friction period, presumably falling outside an individual’s marginal rate of substitution between wealth and mortality risk (since they do not alter the individual’s health or consumption) and are therefore not captured by VSL estimates. Healthcare costs averted were estimated by multiplying the number of hospitalizations averted by costs per hospitalization. These healthcare costs are assumed to be borne by health systems or insurers rather than incurred out-of-pocket by individuals and thus do not overlap with VSL-based valuations. We acknowledge that individuals sick with COVID-19 likely incurred some out-of-pocket costs, but for the purposes of this analysis we assume these are captured within VSL estimates. GDP deflator indices were used to control for inflation for costs that were reported in years other than 2021. Further details are in Appendix B. Since costs and benefits were only considered for one year, discounting was not applied to the costs. However, since life years gained extend beyond this first year, discounting was applied to QALY estimates adjust for preference for immediate benefits. Overall, the health and economic benefits considered in the CBA included averted mortality valued by VSLs and reduced third-party healthcare and productivity costs due to averted mortality and morbidity; while the CEA’s health and economic benefits included averted mortality and morbidity measured using QALYs and reduced third-party healthcare and productivity costs due to averted mortality and morbidity. |
| **Valuation of outcomes** | 13 | Describe the population and methods used to measure and value outcomes. | Methods, Page 9 (Paragraph 1), To estimate vaccine impact, the fitted model was simulated under two scenarios: (i) the observed vaccination rollout, and (ii) a counterfactual scenario in which no COVID-19 vaccines were administered, while all other factors, including non-pharmaceutical interventions, behavioral changes, and variant emergence, were assumed to remain as observed. The difference between these scenarios yielded estimates of infections, hospitalizations, and deaths averted by vaccination. Uncertainty in epidemiological outcomes was propagated by drawing 100 posterior samples per country from the fitted model. These samples were used directly as inputs to the economic evaluation, ensuring that uncertainty from the transmission model was carried through to all health and ROI estimates. Further methodological details are provided in Appendix B and in Watson et al.1 Given that non-pharmaceutical interventions, behavioral changes, and variant emergence were assumed to remain as historically observed, the benefits of COVID-19 vaccination considered in this study are limited to those related to the direct reduction in COVID-19 morbidity and mortality, and do not capture costs and benefits arising from associated policy or behavioral changes enabled by vaccination (such as relaxation of lockdowns). |
| **Measurement and valuation of resources and costs** | 14 | Describe how costs were valued. | Methods, Page 11 (Paragraph 3) to Page 12 (Paragraph 2), Development costs included costs from the pandemic’s start (2020), but excluded any costs related to the basic research that contributed to the vaccine development prior to the pandemic, as these were regarded as sunk costs. Vaccine costs considered in this study include both public and corporate investments into the basic research, development, and manufacturing of COVID-19 vaccines, as well as APAs that were used to accelerate the development of the vaccines. Vaccine research includes funding for development and testing of vaccine candidates and clinical trials.22–24 Development cost breakdowns are in Supplementary Table 1. Costs incurred in 2020 were converted to 2021 USD using GDP deflator indices. Vaccine delivery costs reflect the costs incurred to deliver vaccines to individuals, including human resources and vaccine transport. A single vaccine delivery cost reflecting the average delivery cost for a fully vaccinated individual was used and was multiplied by the number of fully vaccinated individuals to obtain the overall cost of delivering vaccines in each country.25 This delivery cost estimate includes human resources for vaccine administration, training and supervision, cold-chain infrastructure and maintenance, transportation and logistics, social mobilization and communication activities, data systems and monitoring, waste management, and other service delivery-related inputs. The estimate was obtained from a study modelling COVID-19 vaccine delivery costs across lower-middle income countries (LMICs) using a standardized, aggregate delivery costing framework informed by global data and country-level parameters and was applied to all countries included in the current study. Immunization related costs including side effects and productivity losses from attending vaccination, were not included in this study. |
| **Currency, price date, and conversion** | 15 | Report the dates of the estimated resource quantities and unit costs, plus the currency and year of conversion. | Methods, Page 10 (Paragraph 1), All costs are reported in US$2021. |
| **Rationale and description of model** | 16 | If modelling is used, describe in detail and why used. Report if the model is publicly available and where it can be accessed. | Methods, Page 8 (Paragraph 2), To accurately assess the impact of COVID-19 vaccination on mortality and morbidity, we used the results of a previously published global transmission model-based analysis of COVID-19 vaccination impact by Watson et al.1 |
| **Analytics and assumptions** | 17 | Describe any methods for analysing or statistically transforming data, any extrapolation methods, and approaches for validating any model used. | Methods and Appendix |
| **Characterising heterogeneity** | 18 | Describe any methods used for estimating how the results of the study vary for subgroups. | Not reported. |
| **Characterising distributional effects** | 19 | Describe how impacts are distributed across different individuals or adjustments made to reflect priority populations. | Not reported. |
| **Characterising uncertainty** | 20 | Describe methods to characterise any sources of uncertainty in the analysis. | Methods, end Page 12 to top of Page 14. A probabilistic sensitivity analysis (PSA) was used to propagate parameter uncertainty to the ROI estimates. The PSA used Latin Hypercube Sampling (LHS) to generate 100 sets of values by sampling from the uncertainty distributions of the following parameters: (a) USA VSL value, (b) percentages of GDP used to calculate opportunity cost thresholds for each World Bank income group, (c) QALY losses per infection, hospitalization, and death, and (d) friction periods in high-income countries (HIC). We then used the 100 epidemic model fits previously generated in Watson et al. Each model fit represented a distinct set of epidemic parameters drawn from their respective uncertainty distributions. These outputs were randomly paired with the 100 sets of health-economic parameters sampled using LHS to capture uncertainty in both epidemiological and economic parameters. Median and 95% credible intervals for the combined 100 output sets were then calculated to determine the PSA results. Further details can be found in Appendix B. Partial rank correlation coefficients (PRCC) were calculated to assess the relationship between parameters and epidemiological model samples with reported uncertainties and their outcomes, and consequently the effects of parameter uncertainty on ROI estimates. Outcomes assessed in the PSA were QALYs, friction costs, and VSLs. A few additional sensitivity analyses were conducted. The first was a sensitivity analysis on the CBA, which assessed how the exclusion of productivity and healthcare costs averted in the valuation of the vaccines under this perspective shifted the ROI estimate. This additional analysis is aimed to explore how valuing the vaccines based on their ability to reduce mortality risk alone impacts the ROI estimate. Another CBA-related sensitivity analysis was conducted to vary the monetary value of lives saved by applying the value of statistical life years (VSLY), as per Robinson et al. (2019).21 Country-specific VSLY values were obtained by dividing the VSL by the undiscounted future life expectancy of each country and then multiplying this value by the number of undiscounted life years gained from COVID-19 vaccination. In addition to the country-specific VSLYs, we also estimated an ROI using a uniform global VSLY. This VSLY was derived from a population-weighted mean VSLY from the country-specific VSLYs included in the analysis. This additional analysis was conducted as a contrasting scenario to the base case with country-specific VSLYs, acknowledging that differences in the marginal value of money across country income groups could affect the ROI estimates and the distribution of benefits across income settings, since an equivalent increase in income may gain more utility in a lower-income country. Lastly, we also explored how increasing income elasticity to 1.5 for LMICs and LICs affects the CBA ROI estimates, as per Robinson et al. (2019)’s recommendations.21 All additional sensitivity analyses, excluding the first, included healthcare and productivity costs in the vaccination benefits. |
| **Approach to engagement with patients and others affected by the study** | 21 | Describe any approaches to engage patients or service recipients, the general public, communities, or stakeholders (such as clinicians or payers) in the design of the study. | Appendix |
| **Results** |  |  |  |
| **Study parameters** | 22 | Report all analytic inputs (such as values, ranges, references) including uncertainty or distributional assumptions. | Table 1 |
| **Summary of main results** | 23 | Report the mean values for the main categories of costs and outcomes of interest and summarise them in the most appropriate overall measure. | Results, Page 14 to mid Page 15 and Page 16. |
| **Effect of uncertainty** | 24 | Describe how uncertainty about analytic judgments, inputs, or projections affect findings. Report the effect of choice of discount rate and time horizon, if applicable. | Results, Page 16 (Paragraph 3), Based on the PSA, the CBA produced markedly higher ROI estimates (754 [95% uncertainty interval 307,1564]) compared to the CEA (discounted: 60.2 [95% uncertainty interval 23.7,81.5]; undiscounted: 80.9 [95% uncertainty interval 29.8,111]). Across all approaches, the uncertainty intervals of the ROI far exceeded an ROI of 1 for the first year of the COVID-19 vaccination programme globally. Effects of parameter uncertainty on outcomes are found in Supplementary Fig 2-5. For VSLs, we found a positive correlation between VSL in the USA and averted deaths with increased VSLs (p < 0.001). We also found that cost-effectiveness thresholds were the most influential parameter driving QALYs (p < 0.001), according to the PRCC, showing a strong positive correlation with the value of health outcomes. Lastly, there was a strong correlation between the duration of friction periods (in HICs) and friction costs (p < 0.001), as well as between averted infections and friction costs (p < 0.001). |
| **Effect of engagement with patients and others affected by the study** | 25 | Report on any difference patient/service recipient, general public, community, or stakeholder involvement made to the approach or findings of the study | Not applicable. |
| **Discussion** |  |  |  |
| **Study findings, limitations, generalisability, and current knowledge** | 26 | Report key findings, limitations, ethical or equity considerations not captured, and how these could affect patients, policy, or practice. | Discussion, Page 17 to Page 25. |
| **Other relevant information** |  |  |  |
| **Source of funding** | 27 | Describe how the study was funded and any role of the funder in the identification, design, conduct, and reporting of the analysis | Publication portal |
| **Conflicts of interest** | 28 | Report authors conflicts of interest according to journal or International Committee of Medical Journal Editors requirements. | End of manuscript |

*From:* Husereau D, Drummond M, Augustovski F, et al. Consolidated Health Economic Evaluation Reporting Standards 2022 (CHEERS 2022) Explanation and Elaboration: A Report of the ISPOR CHEERS II Good Practices Task Force. Value Health 2022;25. <doi:10.1016/j.jval.2021.10.008>
