# Supplementary material for: Global return-on-investment of COVID-19 vaccines in the first year of the vaccination programme: A model-based economic evaluation
Source: PLoS Med. 2026 Nov 5;23(11):e1004772. doi: 10.1371/journal.pmed.1004772 (PMC13739145; doi:10.1371/journal.pmed.1004772)
Supplement: Supplementary file 3 [file pmed.1004772.s004.docx]

Review PMEDICINE-D-25-03197R1

The global return-on-investment of COVID-19 vaccines in the first year of the vaccination programme

The paper estimates the return-on-investment (ROI) of COVID-19 vaccines globally by comparing vaccines’ development and manufacturing costs to the (monetized) benefits in terms of reductions in mortality, morbidity, and associated economic costs. The authors find that COVID-19 vaccines have a substantial ROI (larger than $60 for all simulation runs); the exact size depends, among other things, on the adopted framework (welfarist vs. extra-welfarist, discounted vs. undiscounted benefits).

The paper thoroughly shows that the massive investment into rapid development of COVID-19 vaccines paid off. I don’t think that the main finding of the paper will be qualitatively affected, but I have a few comments to increase the strength of the quantitative analysis.

- The large ROI estimates are largely driven by the estimated numbers of deaths/cases averted. The paper relies on results by one modeling study (Watson et al. 2022). The authors mention that these estimates tend to be higher than results from regression models. However, it is difficult for the reader to understand the quality/reliability of these estimates compared to other results from the literature. A more in-depth discussion of the consensus/uncertainty around COVID-19 vaccination impacts would be helpful. For example:
  - Are there other modeling studies that estimate the number of COVID-19 deaths/cases averted by vaccination? How do the Watson et al. estimates compare to the rest of the literature?
  - Is Sevilla et al. the only study estimating the benefits of COVID-19 vaccine with a regression model? That seems unlikely. Then, how do the estimates used in the paper compare with that literature?
  - What about the country-specific confidence intervals derived from Watson et al? How big are they?
- The authors adopt two different evaluation methods to estimate the benefits of vaccination. For the welfarist approach, they use country-specific VSLY estimates and multiply them by the number of life years gained. The proposed welfarist approach does not include productivity losses and healthcare costs because “these costs are assumed to be factored into individuals’ valuations of their preferences for reductions in mortality risk.” The welfarist approach also does not include the value of non-fatal cases. I find both assumptions restrictive:
  - Mortality gains are the most important source of benefits, but I don’t understand why the value of non-fatal cases should be included in the extra-welfarist approach but not in the welfarist one. The authors could derive a willingness-to-pay per QALY from the VSL estimates and multiply this value by the QALY gains as in the extra-welfarist approach.
  - It’s not clear to me that healthcare costs and productivity benefits are already captured in VSL estimates. Willingness to pay for an increase in survival may be affected by the out-of-pocket healthcare costs paid by the individual, but not all COVID-19-associated healthcare costs were paid out of pocket. In a standard BCA calculus, these third-party costs are typically added to the monetized value of deaths avoided. I don’t know if there are estimates of COVID-19 out-of-pocket healthcare costs, but a useful proxy could be WHO estimates of out-of-pocket expenditures. The same argument applies to productivity costs: in addition to a potential individual productivity loss (even though, in countries with social insurance, there may be no loss at all), there is an economy-wide loss that goes beyond the sum of individual ones (e.g., the GDP loss due to non-pharmaceutical interventions).
- In the extra-welfarist approach, the authors determine the productivity loss accounting for absenteeism due to sickness and premature death. Although this is a standard methodology, I am not sure it is appropriate for the COVID case. In particular, it does not seem to fully capture the GDP loss due to economic shutdowns, supply chain disruptions, and other effects of non-pharmaceutical interventions. One of the biggest benefits of COVID vaccination was that it allowed the reopening of the economy and the associated economic benefits (which do not seem to be much correlated with the number of days of work that sick people lost). I would encourage the authors to re-evaluate how to best capture the economic benefits of COVID vaccination.
- The extra-welfarist approach leads to significantly lower estimates than the welfarist approach. One of the main differences comes from how the value of a (quality-adjusted) life year is estimated. In particular, the welfarist approach relies on willingness to pay estimates, while the extra-welfarist approach relies on health-opportunity costs to estimate the willingness to pay thresholds. An alternative method to estimate the thresholds is to rely on willingness to pay estimates (a demand side approach). In that case, there should be basically no difference between what the authors call the “welfarist” and “extra-welfarist” approaches (once all the benefits are adequately accounted for as previously explained). Therefore, the entire paper can be reframed in terms of the impact of willingness-to-pay thresholds on the COVID-19 ROI and how different methods for estimating the threshold affect the results. So, I think my question is: Why is the health-opportunity cost method the best approach to estimate the threshold? And why don’t the authors simply compare different ways of estimating the threshold rather than invoking differences between the welfarist and extra-welfarist approaches?
- About the difference between the welfarist and extra-welfarist approaches, I think it would be useful if the authors included a definition of what they mean with the two terms. There is a debate in the BCA literature whether the Kaldor-Hicks criterion (which is implicitly adopted in the paper) has normative value (i.e., is a standard BCA calculus that compares total monetary costs and total monetary benefits really promoting welfare). Thus, I am not sure I would call standard BCA a “welfarist” approach (which goes back to my comment about defining the terminology).
  - See, e.g., James K. Hammitt, [Positive versus Normative Justifications for Benefit-Cost Analysis: Implications for Interpretation and Policy](https://www.tse-fr.eu/fr/articles/positive-versus-normative-justifications-benefit-cost-analysis-implications-interpretation-and), *Review of Environmental Economics and Policy*, vol. 7, n° 2, 2013, p. 199–218.
- Most importantly, summing income-specific VSLYs across countries without acknowledging differences in the marginal value of money leads to an overestimation of the vaccination benefits in high-income countries and an underestimation of its benefits in low-income countries. I think a discussion of this issues should be included in the paper, especially given that most mortality/morbidity benefits occur in high-income countries. The authors could also include an additional sensitivity analysis where all life years are equally valued with a global VSLY. Or adjust country-specific VSLY estimates with weights as recommended, e.g., in the UK Green Book.
- The discounted welfarist ROI is larger than the undiscounted one. At first sight, this sounds odd. From my understanding, this is due to the fact that zero discounting affects both the total number of life years gained *and* the VSLY. Is this a standard approach? I may be wrong, but I don’t think it is (or, at least, not in the literature that I know). First of all, I think this computation should be explained in the main text. Second, I am not sure what is the rationale here. If VSL derives from individuals’ willingness to trade changes in mortality with changes in income/wealth, then it should account for individuals’ (rational) preferences, including discounting. In contrast, whether each death or life year should count the same is a normative issue, which may have nothing to do with individuals’ preferences (unless you assume that individuals’ preferences dictate normative preferences). I would recommend the authors to spell out what their discounted and undiscounted approaches represent (rather than just saying that this is what the WHO recommends).
- The authors assume that the income elasticity of VSL is equal to 1. Robinson et al. suggests using higher income elasticities for lower-income countries (e.g., 1.5) on the ground that income elasticity seems to vary by income group. This would be an interesting sensitivity analysis.
- The VSL approach adopted in the paper implicitly assumes that COVID was a “small risk” (and thus one can simply multiply VSLY by the number of LYs to determine the overall impact). There was some debate about whether COVID-19 can be considered a small risk or whether a different methodology should be applied. Not sure where the debate landed, but it would be interesting to include such a discussion in the paper.
  - See, e.g., Hammitt JK. Valuing mortality risk in the time of COVID-19. J Risk Uncertain. 2020;61(2):129-154. doi: 10.1007/s11166-020-09338-1.
- I don’t understand how to read Supplementary Table 1. In particular, what is the first column? And what do all those numbers refer to? Different studies?
- It’s not very easy to appreciate the heterogeneity of estimates in Figure 1. Perhaps the authors could use a different set of colors (e.g., going from green to red).
- As a reader, I would also like to have a better sense of the parameter estimates used in the analysis. The authors report the sources of the estimates, but I don’t see any numbers. I understand that there are many parameters and many countries, but it would be useful to add a few tables with summary statistics (e.g., what is the US VSL baseline value used and its range in sensitivity analysis? How many averted deaths/hospitalizations (mean and 95% CI) by income group or region? etc.)
