# Supplementary material for: Global return-on-investment of COVID-19 vaccines in the first year of the vaccination programme: A model-based economic evaluation
Source: PLoS Med. 2026 Nov 5;23(11):e1004772. doi: 10.1371/journal.pmed.1004772 (PMC13739145; doi:10.1371/journal.pmed.1004772)
Supplement: Supplementary file 4 [file pmed.1004772.s006.docx]

Review PMEDICINE-D-25-03197R2.

I thank the authors for their thoughtful revisions and detailed responses to my comments. The paper addresses an important and timely question, and the main finding — that COVID-19 vaccine development yielded substantial returns — is convincing and robust to a range of assumptions. Several of my original comments have been well addressed, including the addition of sensitivity analyses for income elasticity and uniform VSLY, the discussion of non-marginal risk, and the more detailed reporting of parameter estimates and results by income group. However, some comments have not been fully addressed, and the revision of the paragraph on the welfarist and extra-welfarist frameworks has introduced new inaccuracies. I outline my remaining concerns below.

Definition of welfarist/extra-welfarist. I appreciate that the authors now include a paragraph that defines the difference between welfarist and extra-welfarist approaches. However, I don't think the revision fully addresses my original comment, and the added material introduces several inaccuracies.

I think the main issue is that the authors conflate welfarism with something narrower (unweighted CBA or the Kaldor Hicks compensation criterion) and then present extra-welfarism as the solution to limitations that welfarism itself does not necessarily have. Whether the unweighted CBA framework used in the paper constitutes a "welfarist" evaluation is contested in the literature, and in a study of global vaccine ROI, where both VSL and the distribution of benefits vary enormously across income levels, this is not merely a terminological issue. Several specific claims in the revised paragraph reflect this conflation:

- “The welfarist approach does not admit broader societal values.” The specific version used in the paper may not incorporate societal values, but that’s not true for a more general approach – see all the literature on the use of social welfare functions in policy evaluation (e.g., the work by Matthew Adler, Marc Fleurbaey, etc.)
- “The welfarist approach is the dominant view in welfare economics.” Welfare economics is much broader than what the authors consider “the welfarist approach”.
- CBAs are "usually operationalised using VSL." VSL is used for the mortality-risk component of a CBA, but CBAs can encompass many types of benefits and costs — time savings, morbidity, productivity, environmental quality, etc. The paragraph makes it sound like VSL is the CBA methodology, when it's one input.
- VSL "captures the amount of consumption that an individual is willing to give up to reduce their own mortality risk." VSL is the marginal rate of substitution between wealth (or income) and mortality risk — it's derived from WTP for small risk reductions, then scaled up.
- The authors also write that “In principle, this could give similar results to a welfarist approach if

health is valued based on individual willingness-to-pay.” This is logically correct, but in a paragraph that is building a case for why both approaches are used, it undercuts the argument for why extra-welfarism is needed.

I would encourage the authors to define their two approaches operationally, stating what each includes, how benefits are valued, and why — as the original version of this paragraph did. If the authors wish to additionally frame these approaches as “welfarist” and “extra-welfarist”, they should provide clear definitions of what they mean by these terms in the context of this study, rather than characterizing the frameworks in general terms.

Double counting in the welfarist case. One of my original comments was about the absence of third-party costs in the CBA calculation. In this revision, the authors have added healthcare costs and productivity losses to both the “extra-welfarist” and CBA approaches. But the VSL is already supposed to include costs borne by the individual, thus leading to double counting. What I call third-party costs are healthcare costs borne by the healthcare system/insurance provider and productivity losses borne by other people (e.g., those who are not sick but required to miss work because of economic lockdowns). In the sensitivity analysis, the authors show that this issue is quantitatively minimal (excluding the added costs has not substantial effect on the ROI, even though this is because they underestimate the productivity loss – see my comment below on GDP loss); however, it is conceptually an important issue and the choice made by the authors is not justified or discussed.

Value of nonfatal outcomes in the welfarist case. In their response, the authors present the omission of morbidity from the welfarist approach as if it is simply a feature of the method rather than a limitation or a choice that could have been made differently. In the paper, there is no discussion of why non-fatal effects are excluded from the welfarist approach, and no acknowledgment that this understates welfarist benefits.

**GDP loss from lockdowns/economic reopening benefits.** The productivity costs still only capture absenteeism and death (lines 136–139). The massive economic benefit of vaccines enabling reopening is still missing from the analysis. I understand that there are methodological limitations here linked to the assumptions in the underlying transmission model. However, this issue should at least be acknowledged in the limitation section. The paper's framing — "global return-on-investment," whether the investment "paid off" — implies a comprehensive assessment of whether vaccine development was worth it. If what may be the single largest economic benefit of vaccination is excluded, the reader should understand that the ROI estimates are likely to be conservative for this reason. Neither the welfarist nor the extra-welfarist approach are incompatible with including the GDP loss, as the authors seem to imply in their response.

Minor comments:

- I don’t think the term “welfarist economics” exist
- I don’t see a definition of the acronyms BCAs and CBAs
- The study’ perspective is defined as “global payer perspective”. Maybe societal perspective at the global level would be a better characterization. It’s just that when people see “payer perspective” they automatically think of a narrow conception of costs and benefits – which does not correspond to what the authors do here.
- Line 132 says that QALYs were “monetised by applying societal willingness-to-pay (WTP) thresholds”. I think calling these values WTP thresholds mischaracterizes what the extra-welfarist estimates actually represent (opportunity cost thresholds). What society is willing to pay for health does not necessarily coincide with the marginal productivity of health spending (what a health system gives up when it funds one intervention rather than another).
- I don’t understand why the authors are introducing a new concept (VLY) as opposed to VSLY
- I am afraid I still don’t understand Table 1 in the SI (is each row a different value for that category?)
