# Supplementary material for: Global return-on-investment of COVID-19 vaccines in the first year of the vaccination programme: A model-based economic evaluation
Source: PLoS Med. 2026 Nov 5;23(11):e1004772. doi: 10.1371/journal.pmed.1004772 (PMC13739145; doi:10.1371/journal.pmed.1004772)
Supplement: Supplementary file 5 [file pmed.1004772.s007.docx]

## Reviewer #1:

### Comments:

***A.*** *The paper "The global return-on-investment of COVID-19 vaccines in the first year of the vaccination programme" by Benjamin et al., addresses a key gap in the literature by assessing the economic value of COVID-19 vaccines in first year after their global roll out. It shows that vaccines played a major role in reducing the global health burden during the pandemic and estimates that for every dollar spent on development and delivery in the first year, the return ranged from $59 to $475. These findings highlight the extraordinary value of investing in vaccines, both for public health and the global economy but also showcase how unequal benefits are distributed globally.*

*It is a necessary study that will be of interest to the readers of the journal. I did not completely follow a few decisions the authors made and appreciate that my points below might be addressed by improving the clarity and provided rationales. However, alternatively major revisions may be required, especially re the mortality risk reduction valuation.*

Response: **Thank you for acknowledging the value of our study in contributing to the literature and promoting investments into future vaccine efforts. We hope that our revisions using your comments help strengthen the quality of our work.**

***B.*** *The introduction provides a concise and engaging overview of the existing literature, the research gap, and the study's objectives. However, the language could be clearer in places. For instance, the authors refer to "development, manufacturing, and distribution costs," but it's not immediately clear what these encompass. In the methods section, "delivery costs" are mentioned, yet it remains ambiguous whether this includes costs beyond the point of vaccination—such as storage at administration sites or the costs of administering the vaccine. Clarifying this distinction would strengthen the transparency of the cost analysis and this needs to be taken care off across the whole work. Finally, the last sentence of the introduction could be simplified to more clearly convey its meaning and relevance to the results section.*

**Response: Thank you for these suggestions to clarify our language. We faced challenges describing the investments made into COVID-19 vaccine research and development, given that many publicly announced investments lacked granular detail on what the funds were exactly used for. We added the following sentences to describe the costs of the vaccines to the best of our abilities with publicly available information. We also added this as a limitation to our paper.**

**Page 11, Lines 150-153: “Vaccine costs considered in this study include both public and corporate investments into the basic research, development, and manufacturing of COVID-19 vaccines, as well as APAs that were used to accelerate the development of the vaccines. Vaccine research includes funding for development and testing of vaccine candidates and clinical trials.^19–21^”**

**Page 11, Lines 159-165: “This delivery cost estimate includes human resources for vaccine administration, training and supervision, cold-chain infrastructure and maintenance, transportation and logistics, social mobilization and communication activities, data systems and monitoring, waste management, and other service delivery-related inputs. The estimate was obtained from a study modelling COVID-19 vaccine delivery costs across LMICs using a standardized, aggregate delivery costing framework informed by global data and country-level parameters and was applied to all countries included in the current study.”**

**Page 23, Lines 441-445: “The certainty of our results is also limited by the lack of granular publicly available information on what investments made into vaccine research, development, and manufacturing were used for. Given this limitation, we took a maximal approach in estimating the costs to be cautious of this uncertainty yet still estimated a substantial net positive ROI.”**

**In addition, we have simplified the last sentence of our introduction as per your suggestion. The sentence now reads (Page 7, Lines 53-55): “We also aimed to estimate the wider societal benefits of COVID-19 vaccination, including reduced mortality and morbidity, as well as improved productivity, and to compare these benefits generated across World Bank income groups.”**

***C.*** *The methods section is concise, maybe to conscise, but useful detail is provided in the appendix. However, the authors should more clearly explain how costs and benefits were aggregated and monetised. Referring to the *Reference Case Guidelines for Benefit-Cost Analysis in Global Health* (Robinson et al., 2019) could help clarify this, even if the approach does not explicitly follows this guidance. It should be explicitly stated whether immunisation-related costs—such as administration, side effects, and productivity losses from attending vaccination—are included or excluded and the implications of the decision could be discussed in the relevant section. A table or graphic summarising inputs, outcomes, and monetisation approaches would greatly improve clarity and transparency.*

**Response: Thank you for this suggestion. We have further elaborated on our methods and moved material from the Appendix to the main text to enhance readers’ understanding. Additionally, we believe conceptual diagram (Fig 1.) below can help better understand how costs and benefits were aggregated and monetized.**

**Additions:**

**Page 11, Lines 157-166: “A single vaccine delivery cost reflecting the average delivery cost for a fully vaccinated individual was used and was multiplied by the number of fully vaccinated individuals to obtain the overall cost of delivering vaccines in each country.^22^ This delivery cost estimate includes human resources for vaccine administration, training and supervision, cold-chain infrastructure and maintenance, transportation and logistics, social mobilization and communication activities, data systems and monitoring, waste management, and other service delivery-related inputs. The estimate was obtained from a study modelling COVID-19 vaccine delivery costs across LMICs using a standardized, aggregate delivery costing framework informed by global data and country-level parameters and was applied to all countries included in the current study. Immunisation related-costs including side effects and productivity losses from attending vaccination, were not included in this study.”**

**Fig 1. An overview of all study inputs, outputs, costs, and benefits.**

***D.*** *Moreover, the rationale for valuing mortality risk reduction using life years gained multiplied by VSLY, rather than number of deaths multiplied by VSL (or both), should be explained in the main text. Since VSLY introduces an implicit age adjustment of mortality risk reduction, recent guidance (e.g. Robinson et al., OECD 2023) recommends presenting this as a sensitivity analysis. This would also improve comparability with studies such as Owsawa et al., even if it results in higher ROI estimates. This choice is therefore unclear to me and there should be at least a strong rational for why VSL valuation was not performed.*

**Response: Thank you for this suggestion. After carefully reviewing Robinson et al., we decided to add VSL as our main outcome and present VSLYs as a sensitivity analysis.**

**Page 10, Lines 129-133: “For the welfarist analysis, deaths averted were monetised by apply the value of statistical life (VSL). Following recommendations from Robinson et al.,^18^ VSLs were derived using country-specific income adjustments. For the extra-welfarist analysis, undiscounted and discounted QALYs gained were monetised by applying societal willingness-to-pay (WTP) thresholds, which are based on income-group dependent percentages of gross domestic product (GDP) per capita. Further details are in Appendix B.”**

**Page 13, Lines 192-196: “Another analysis was conducted within the welfarist perspective to determine the monetary value of lives saved by applying the value of statistical life years (VSLY), as per Robinson et al. (2019).^18^ Country-specific VSLY values were obtained by dividing the VSL by the undiscounted future life expectancy of each country to get the value of a life year (VLY). This VLY is then multiplied by the number of undiscounted life years gained from COVID-19 vaccination.”**

**Page 14-15, Lines 233-237: “From the welfarist perspective, it is estimated that COVID-19 vaccination generated economic value of $63.5 trillion (95% CrI 61.4 – 66.6) in VSLs from averting deaths. HICs experienced the highest gains in VSLs, while LICs experienced the lowest gains. However, when expressed as per-person vaccinated as a percentage of GDP per capita (GDPpc), HICs and LICs incurred higher gains than UMICs and LMICs. Table 2 reports VSLs globally and across the World Bank income groups.”**

**Page 16, Lines 274-276: “When using VSLY estimates in the welfarist perspective, a ROI well more than 1 was still observed. VSLY estimates globally and per income group can be found in Supplementary Table 4.”**

**Page 17-18, Lines 310-320: “Second, when averted mortality benefits were valued using country-specific values of VSLYs, a reduction in the estimated ROI was observed, yet did not change the interpretation of our results. This finding was expected, as VSLY-based calculations capture the value of life years gained rather than deaths averted. Thus, an averted death in an older individual would yield lower value as less life years were gained, in comparison to a younger individual. Given that COVID-19 disproportionately affected older populations, valuing the vaccines based on the number life years gained generates lower estimated monetized benefits in comparison to valuing them based on deaths averted, as is the case with VSL-based approaches. The value of VSLYs gained increased across income groups, reflecting both higher willingness to pay for mortality risk reductions and longer life expectancy in HICs, which together yield a greater monetized value of life years gained.”**

***E.*** *It is also not clear to me why the welfarist approach would exclude all the value from non-fatal events? They might be small compared to mortality risk reduction, I agree, but the authors should lay out their rational why productivity costs averted for those who survive and HCRU is not included.*

**Response: Thank you for this comment. After consideration and reviewing the literature, we agree that productivity costs and healthcare utilization costs averted for those who survive should be included in the welfarist approach. We have now changed our methods and results to reflect this addition. Adding the productivity and healthcare costs to the welfarist perspective did not meaningfully change the results as they only contribute less than 1% to the overall welfarist benefits. We also created a sensitivity analysis where we limit the welfarist approach to only VSLs to assess how the ROI estimate changes in response to valuing the vaccines based on averted mortality alone.**

***F.*** *The results are adequately described and clearly presented. However, the authors may wish to consider whether a graphical representation of parts of the results section would be worthwhile.*

**Response: Thank you for this suggestion to enhance the clarity of our results section. We have decided to include an additional figure, Fig 3, to graphically represent our results. This figure depicts the proportion of each economic benefit to the overall benefits under both the welfarist and extra-welfarist perspectives.**

**Fig 3. Relative contribution of benefit components to total economic benefits under the extra-welfarist and welfarist approaches. All contributions are expressed as proportions of the total estimated monetized benefits within each approach.**

***G.*** *The discussion is engaging and well-structured, but the authors should place greater emphasis on the implications of how mortality benefits are valued. Specifically, more attention should be given to what VSL and VSLY represent in terms of tangible and intangible benefits, and how these choices influence the overall ROI estimates. For example, in the extra-welfarist approach results section, the productivity gains alone appear to exceed the total development and delivery costs by a significant margin, which should be highlighted as it is of interest to decision makers sitting in the relevant departments for economic planning. Clarifying these valuation choices and their impact would strengthen the interpretation of the results and help readers better understand the drivers of the estimated returns.*

**Response: Thank you for this important suggestion. We have added the following paragraph which we believe has strengthened our discussion and helps explain what the drivers of the ROI estimate are and the relevance of this for policy decision making.**

**Added paragraph on page 17, lines 292-300: “Under the welfarist perspective, averted mortality accounts for >99% of the high ROI estimate, reflecting the high value of individuals place on reductions in mortality risk. Under the extra-welfarist perspective, averted mortality and morbidity, quantified by QALYs gained, contribute most strongly to the estimated returns, highlighting the considerable intrinsic economic value of health gains. However, even the tangible economic benefits such as improved productivity exceeded the total global cost of vaccine development and delivery, indicating that investments into COVID-19 vaccines would have yielded net positive economic returns from a purely monetary perspective. This finding highlights the importance of vaccination for economic planning and development, in addition to public health and health systems.”**

***Reviewer #2:***

### Comments:

***A.*** *This manuscript offers a timely and rigorous health economic analysis of the global return on investment (ROI) from COVID-19 vaccination. The study's strengths are evident in its robust methodology, extensive sensitivity analyses, and clear policy relevance. The authors employ both extra-welfarist and welfarist perspectives to monetize health benefits, demonstrating strong technical rigor. The results - showing ROIs far exceeding 1 under all assumptions - convincingly underscore that COVID-19 vaccination is a highly cost-beneficial public health investment. The manuscript is well-written and the discussion appropriately acknowledges key drivers (e.g. differential benefits by income level) and limitations. In summary, this is an important and well-executed study that provides valuable evidence to policymakers about the enormous economic and health returns of vaccination. Only a few minor clarifications and additions are recommended below to further strengthen the context and clarity of the work.*

#### Response: Thank you for your positive feedback and for highlighting the strengths of our work. *B.* *Contrast with Prior ROI Estimates: To bolster the discussion, the authors could consider explicitly contrasting their ROI findings with those of previous studies. For instance, various papers including (but not limited to!) Theakston et al., (2025) and Harrison et al., (2025) report an ROI of roughly 19:1 for adult immunization programs when broader societal benefits are monetized. Further, El Banhawi et al., (2025) emphasize that analyses centered on the endemic period also found that the benefit of COVID-19 vaccination is large including to the national economy by averting productivity losses (in addition to healthcare costs). The authors are encouraged to note that differences in ROI estimates across studies likely stem from the focus on short-term, peak pandemic effects versus longer-term endemic vaccination strategies. By situating their results in this context, the authors can clarify that their own ROI estimates, while high, are nevertheless relatively conservative (covering the first-year program with comprehensive but bounded assumptions) and complementary to prior published estimates. This addition will reinforce the message that, whether one examines the intense initial pandemic phase or more routine immunization periods, COVID-19 vaccination consistently delivers a high return on investment under various scenario. This would strengthen the paper's positioning in the literature and assure readers that the findings align with - and add to - the growing evidence base on vaccination's value.*

#### Response: Thank you for the suggestion to bolster our discussion and for the references provided. We agree that contextualizing our results with these other papers can help strengthen our positioning within the growing evidence base on vaccination’s value. We have added the following to our discussion:

**Page 19, Lines 341-356: “Our findings are also consistent with other ROI analyses of adult respiratory vaccination. Recent studies have reported ROIs of approximately 19:1 for adult respiratory vaccination, including COVID-19, when broader societal benefits are monetised.^31^ These studies adopted longer time horizons ranging from five years to a cohort’s lifetime, reflecting the sustained benefits accrued during endemic periods under routine immunisation strategies. In contrast, our analysis focused on the first year of the COVID-19 vaccination programme during the period of peak pandemic transmission, morbidity, and mortality. Differences in ROI estimates across studies are likely due to the differences in time horizon and consequently epidemiological context, rather than inconsistencies in the underlying economic value of vaccination. Our study focused on the short-term peak pandemic effects to examine whether the extraordinary investment in vaccine development and delivery in 2020-2021 was justified, whereas these other studies evaluate the longer-term endemic vaccination strategies. Consistent with this interpretation, El Banhawi et al. (2025) report that COVID-19 continues to impose a major economic burden several years after the onset of the pandemic, and that COVID-19 vaccination continues to reduce morbidity and healthcare utilisation, especially during the flu season.^32^ Taken together, these findings indicate that COVID-19 vaccination delivers high economic benefits both during peak transmission and acute pandemic phases and under longer-term endemic vaccination strategies.”**

***C.*** *Terminology Clarification: For broader readership clarity, consider adding a concise explanation of the "welfarist" vs "extra-welfarist" approaches when first mentioned. Not all readers (e.g. policymakers or general public health audience) will be familiar with these health economics terms. A brief description of how the welfarist approach monetizes life-years (e.g. via value of statistical life or VSLY) versus the extra-welfarist approach (focused on direct costs, QALYs, and cost-of-illness savings) would help readers intuitively understand the two ROI figures presented for each scenario. This will ensure the impressive ROI results are interpretable to non-economist stakeholders.*

**Response: Thank you for this suggestion to improve the clarity of our work. We added the following to our methods:**

**Page 7-8, Lines 62-83: “The welfarist approach is the dominant view in welfare economics.^6^ Welfarist economics is based on maximizing individuals’ utility (the utility principle) so the ‘goodness’ of a situation is judged based on the utility derived from it.^11^ CBAs are generally considered to be a welfarist analysis as they judge a policy choice based purely on whether it increases aggregate individual utility, usually measured using the ‘value of a statistical life’ (VSL), which captures the amount of consumption that an individual is willing to give up to reduce their own mortality risk.^12^ VSLs are derived from stated (i.e. surveys) and revealed (i.e. data about how individuals act in real-world situations) preferences of individuals’ willingness-to-pay (WTP) for marginal reduction in their mortality risk.^12^ Hammitt^13^ has pointed out that that BCAs may not always be strictly welfarist since they involve analytical choices that correct individual preferences for various perceived biases, and whether it is welfarist or extra-welfarist depends on the justification adopted.**

**Furthermore, the welfarist approach does not admit broader societal values. These societal values may not align with individual preferences, particularly in health contexts. For instance, society may value good health and health improvements differently from individuals, particularly those with less income. To address these limitations, an extra-welfarist approach is adopted. Extra-welfarism differs in that it does not solely focus on individual’s utilities but assigns an intrinsic value to outcomes like ‘health’ and the inherent value of saving lives.^11^ In principle, this could give similar results to a welfarist approach if health is valued based on individual willingness-to-pay. Evaluations following extra-welfarist approaches include cost-effectiveness analyses (CEAs), which compare an interventions’ costs to its health outcomes, often use quality-adjusted life years (QALYs), which provide a non-monetary measure that combines both the quality and quantity of life, enabling the evaluation of health interventions based on the amount of health they produce,^14^ irrespective of monetary valuations of individual preferences.^14^”**

***D.*** *Contextualize ROI as Conservative: The discussion could more plainly state that the ROI estimated here is likely conservative (particularly under the extra-welfarist perspective) yet emphasize that despite this it is still extraordinarily high, reinforcing that even under cautious assumptions COVID-19 was and remains a sound investment. Another suggestion is to also more plainly state the the ROI using VSL estmates does not mean that every dollar invested will deliver the estimated return in cash. The authors are very prudent already by consistently using the term economic returns rather than financial but it may be good to leave no doubt as some readers may otherwise not fully appreciate this important nuance.*

**Response: Thank you for this comment. To enhance our discussion, we have added the following paragraph (page 23-24, lines 459-466): “This study’s ROI estimates are likely conservative, as health and economic benefits from the vaccines continued to accrue beyond the first year of this administration. The extra-welfarist framing, which values vaccine benefits solely in terms of health outcomes, productivity, and cost savings, may be particularly conservative. Despite these cautious assumptions, COVID-19 vaccines still emerge as a sound global investment.**

**The benefits valued using VSLs and QALYs gained represent economic valuation of health gains rather than financial returns or cash flows. These measures capture individual or societal willingness to pay for reductions in mortality and morbidity, rather than direct fiscal savings.”**

***E.*** *Minor Edits and Clarity: The manuscript is generally clear and well-structured. I encourage the authors to double-check that all acronyms (e.g., ROI, QALY, VSLY, LMIC, etc.) are defined at first use for clarity. In addition, ensure that figures and tables are referenced in order and that their captions are self-explanatory. For example, Table 4 presents ROI results; a brief mention in the text guiding the reader to "Table 4 shows that all ROI estimates exceed 1 (i.e., net positive returns)" would be helpful. Just some small editorial suggestions to enhance readability.*

**Response: Thank you for the suggestion and for this fine attention to detail. We have checked and updated our manuscript accordingly to define all acronyms at the first use. We have also made sure to reference our figures and tables in order as well as improve the clarity of their captions.**

**We have made the following changes in our results section:**

**Page 14, Lines 237: “Table 2 reports discounted and undiscounted VSLYs globally and across the World Bank income groups.”**

**Page 15, Lines 242-244: “Table 3 reports the discounted and undiscounted monetized QALYs globally and across the World Bank income groups.”**

**Page 15, Lines 260-261: “Table 5 shows that all ROI estimates are well in excess of 1 (i.e., net positive returns) for COVID-19 vaccines globally for the first year of the vaccination programme, under both approaches.”**

***F.*** *Overall, the authors are to be commended for a high-quality analysis. With the above minor additions, the paper will convincingly communicate that investment in COVID-19 vaccination yields substantial health and economic dividends, complementing earlier estimates and providing a strong evidence-based argument for continued support of vaccination programs globally. The revisions will further solidify the manuscript's contribution and clarity for both the journal's academic readership and decision-makers who may act on its findings. Congratulations on an excellent study.*

**Response: Thank you very much for this positive feedback and for the important revisions to improve the quality of our work.**
________________________________________
Theakston C, Napier M, Brassel S, et al. A benefit-cost analysis quantifying the broader socioeconomic value of adult respiratory vaccination programs in Japan. Expert Rev Vaccines. 2025;24(1):633-643.
Harrison C, Brassel S, Chowdhury S, et al. The broader socio-economic value of adult respiratory disease vaccinations in the UK - results from a benefit cost analysis. Expert Rev Vaccines. 2025;24(1)
El Banhawi H., Hodgson S., Neri M., Steuten L. The broader economic benefits of COVID-19 vaccination (2025). Contract Research. Available from <https://www.ohe.org/publications/the-broader-economic-benefits-of-covid-19-vaccination/>.

## Reviewer #3:

***A.*** *Thank you to the authors for submitting this paper, it is clearly on an important topic.

This is clearly a well written paper, but to be honest I'm struggling to see why this needs to be published given the previous evidence base in this area. Some specific comments below.*

**Response: Thank you for the positive comment on the quality of our paper. We hope that our responses to your guiding feedback further explains the importance of our paper in comparison to other previously published studies.**

***B***. *I think the authors need to further justify the need for the paper. There have seemingly been a variety of economic evaluations at a country level. What is the specific need for a combined study also including the vaccine development?*

**Response: Thank you for this suggestion. This study addresses a specific gap in the literature by evaluating the global health and economic benefits of COVID-19 vaccination in relation to the cumulative costs of vaccine development, manufacturing, and delivery. Unlike most vaccine initiatives, COVID-19 vaccine development was driven primarily by state actors (e.g. US federal government) and transnational organizations (e.g. CEPI), rather than by private developers pursuing sales to individual countries. Development proceeded at exceptional speed (and at substantial cost) in a race to mitigate the pandemic’s impact.**

**As a result, a global economic evaluation is not simply the aggregation of country-level analyses. Instead, it must answer a broader question: was the overall global investment worthwhile? While numerous economic evaluations have been conducted at the country level, these analyses typically focus on national procurement and programme implementation costs and do not capture the substantial upstream investments that made vaccines available in the first place.**

**The development and large-scale production of COVID-19 vaccines were unprecedented, relying on coordinated global partnerships and extensive public-sector subsidies to de-risk research, development, and manufacturing at an accelerated timescale. As such, a global analytical framework is required to assess whether these collective investments yielded commensurate returns for the global population. We have revised the background to more clearly articulate this rationale and to clarify the necessity of a global ROI analysis that explicitly incorporates vaccine development costs alongside downstream health and economic benefits.**

**Addition on page 5-6, lines 18-27: “Additionally, the growing literature of economic evaluations on COVID-19 vaccines has primarily assessed the value of vaccines on the country level, limiting their analyses to national procurement and delivery costs. These analyses do not capture the full scale of investments that enabled vaccine availability in the first place. COVID-19 vaccine development was unique, because the vaccines’ rapid development required substantial, coordinated public investment across countries, including direct funding of research and development and advanced purchase agreements that de-risked private-sector production. These investments were made upstream of national vaccination programmes and generated global health and economic benefits that extend beyond any single country’s health system.^4^ As governments and global institutions consider how best to protect global populations in future pandemics, understanding the global economic returns to these upstream investments is critical.”**

***C.***  Need a lot more about the Watson et al model in the main text. This is a key part of the paper and there's one paragraph on it

**Response: Thank you for this suggestion. We agree that the Watson et al. transmission model is fundamental to all health and economic estimates in this study and therefore warrants clearer description in the main text.**

**We have substantially expanded the Methods section to provide a concise but detailed summary of the Watson et al. model, including its structure, calibration approach, data sources, counterfactual assumptions, age stratification, and uncertainty propagation. This description clarifies how estimates of deaths, infections, and hospitalizations averted by vaccination were generated and how uncertainty from the epidemiological model is carried forward into the economic analysis. Specifically, we have added (page 8-8-9, lines 84-106):**

**“To accurately assess the impact of COVID-19 vaccination on mortality and morbidity, we used the results of a previously published global transmission model-based analysis of COVID-19 vaccination impact by Watson et al.^1^. This study used an age-structured, country-specific transmission model of SARS-CoV-2 to estimate the number of infections, hospitalisations, and deaths averted by COVID-19 vaccination during the first year of global rollout (8 December 2020 to 8 December 2021) for seventeen discrete age groups (0-5, 5-10, ..., 75-80, 80+) (Table 1). The model is a deterministic compartmental transmission model stratified by age and vaccination status, which models SARS-CoV-2 infection, progression to severe disease, hospitalisation, and death. The model accounts for age-specific susceptibility, severity, and infection fatality risk and incorporates country-specific demographic structures, contact patterns, and vaccination roll-out. The model was fit to excess all-cause mortality^14^ to mitigate biases arising from under-ascertainment and differences in COVID-19 death reporting systems globally. Model parameters were inferred separately for each country. The fitting framework explicitly incorporated uncertainty in key epidemiological parameters, including transmission intensity, infection fatality ratios, and vaccine effectiveness against severe outcomes.**

**To estimate vaccine impact, the fitted model was simulated under two scenarios: (i) the observed vaccination rollout, and (ii) a counterfactual scenario in which no COVID-19 vaccines were administered, while all other factors, including non-pharmaceutical interventions, behavioural changes, and variant emergence, were assumed to remain as observed. The difference between these scenarios yielded estimates of infections, hospitalisations, and deaths averted by vaccination. Uncertainty in epidemiological outcomes was propagated by drawing 100 posterior samples per country from the fitted model. These samples were used directly as inputs to the economic evaluation, ensuring that uncertainty from the transmission model was carried through to all health and ROI estimates. Further methodological details are provided in Appendix B and in Watson et al.¹”**

***D.*** I would strongly suggest that the authors drop the undiscounted analysis, there is simply no need to present these results if the time horizon of the analyses is beyond one year.

**Response: Thank you for this suggestion. After careful review of Robinson et al. (2019), we dropped the undiscounted analysis for the welfarist perspective. Following our revisions, our main analysis for the welfarist perspective considers VSL estimates and productivity and healthcare costs averted. As such, it does not include discounting. However, as per Robinson et al. (2019) recommendations, we used our VSLY estimates in an additional analysis, given that COVID-19 disproportionately effects older populations. As such, this additional analysis uses undiscounted life years to determine the VSLYs gained from averted COVID-19 mortality (which can occur in the years beyond the intervention time horizon of the model). Additionally, we present both undiscounted and discounted extra-welfarist ROIs, as per Robinson et al (2019).**

***E.*** Need more information from Appendix B in the main text related to the benefits. Nobody really reads the appendices.

**Response: As per your suggestion, we added more information from Appendix B to the benefits, specifically related to the Watson et al. modelling. We believe that the addition to our methods to address your comment C help improve readers’ understanding of our methods.**

**These changes can be found on page 8-9, lines 84-106.**

***F.*** The numbers quoted in the results are so large and heterogenous that they don't really mean anything. I think more work needs to be done to put these into more context (if possible)

**Response: Thank you for this suggestion. To help contextualize our results, we have added the percentage of global GDP that monetary results correspond to. Additionally, we created Fig 2 to visually depict the proportion of the overall benefits within each perspective that each health benefit and economic benefit contributes to.**

**Added lines to results:**

**Page 15, lines 252-254: “Under the welfarist approach, the benefits, including VSLs and productivity and healthcare costs averted, sum to an estimated total of $64.3 trillion (95% CrI 62.1 – 67.3), which is 82.7% (95% CI 80.0 – 86.7) of the global GDP.”**

**Page 15, lines 245-246: “Productivity costs saved from averted COVID-19 morbidity and mortality are estimated to be $564 billion (95% CrI 541–586) and 0.73% of global GDP (95% CrI 0.70 – 0.75)…”**

**Page 15, lines 247-249: “COVID-19 vaccines were estimated to yield additional savings of $222 billion (95% CrI 213 – 229) in healthcare costs, which is an estimated 0.29% of global GDP (95% CrI 0.27 – 0.30)”**

**Added figure: Fig 3. Relative contribution of benefit components to total economic benefits under the extra-welfarist and welfarist approaches. All contributions are expressed as proportions of the total estimated benefits within each approach.**

***G.*** For me, the headline results (estimated returns of $42 - $775 per dollar) are so heterogenous that they're pretty meaningless. This is no surprise given the limitations regarding the data mentioned later in the discussion. Perhaps these need to be presented differently (i.e. under what conditions would the return be $42, under what conditions would the return be $775 etc)

**Response: Thank you for this comment. The range of ROI estimates in our paper are from a base case scenario analysis of two approaches to value economic and health benefits of COVID-19 vaccination globally, as well as probabilistic sensitivity analyses (PSA). After adjusting our welfarist approach to be based on VSL estimates, our ROI estimates have changed; however, this change did not introduce any meaningful differences in our results. Hence the difference in the two values do not primarily reflect uncertainty in our data or modelling methods, but fundamentally different philosophical views on how health should be valued. We think one of the strengths of our study is that (unlike most other economic evaluations) we have captured both views and shown that they both lead to the same overall conclusion.**

**The lower ROI estimate of $59.6 per dollar invested is from base case analysis of the extra-welfarist perceptive, where benefits are primarily driven by discounted monetized QALYs gained. The monetized QALYs are based on conservative, societal willingness-to-pay thresholds for improvements in quality of life and discount future benefits incurred from adverted morbidity and mortality due to COVID-19. In contrast, the upper estimate of $809 per dollar invested is from the base case analysis of the welfarist perspective, in which averted mortality is valued using VSL-based estimates that reflect individuals’ willingness to pay for mortality risk reductions. Regardless of the approach and uncertainty in our parameters, our results estimate that there will be a net positive ROI (i.e. a return of more than $1 per dollar invested) for COVID-19 vaccines in the first year of the vaccination program. We highlight this effect of approaches on the ROI estimates through the following addition to our discussion (page 16-17, lines 284-286): “Globally, for every dollar invested in vaccine development and delivery, the estimated returns ranged from $59.6 to $809, depending on the valuation approach (i.e., welfarist or extra-welfarist approach.”**

**Your suggestion also emphasises the importance of parameter uncertainty and how it effects our estimates. To clarify how specific parameters affect the ROI estimates, we added the following sentences to the discussion (Page 22, lines 429-440): “Other limitations include the lack of precise data and uncertainty estimates around health utility values and …. This study used QALY loss estimates per symptomatic infections, hospitalizations, and ICU admissions, which may not reflect the true QALY losses experienced by those who were infected or hospitalized with COVID-19, including the losses experienced by those affected by long COVID-19. Some infections may be more severe than others, and our use of one value for infection-related QALY losses may not capture the heterogeneity in the clinical outcomes of COVID-19 and the subsequent impacts on quality of life. Additionally, this study used one value for a cost of vaccine delivery for each country that was determined from a study in LMICs. Details on COVID-19 delivery costs, specifically in HICs, where the delivery costs are assumed to be higher than the value used, would enhance the ROI estimate’s precision and likely yield a lower ROI estimate for both approaches by increasing the costs of vaccine delivery.”**

***H.*** Alternatively, could the presentation of these results be reframed as upper/lower bounds of the ROI?

**Response: Thank you for this suggestion. We believe the changes we implemented to help contextualize our range of results as per your above suggestions help address this comment as well.**

***I.*** *I would personally emphasise the disparity amongst income groups more (as for me this is the most interesting part of the study)*

**Response: Thank you for this suggestion. We agree that the disparity amongst income groups is an important aspect of our study that should be given greater emphasis.**

**We have added the following changes to our discussion to emphasis this importance of this finding:**

**Page 17, lines 286-287: “Despite this net positive return globally, there were disparities in the health and economic benefits generated from COVID-19 vaccination across World Bank income groups.”**

**Page 24, lines 459-462 “Our findings highlight …, as well as the importance of equitable vaccine access and distribution to reduce disparities across World Bank income groups.”**

***Reviewer #4:***

***A.*** *This paper makes an important contribution to the literature. It calculates, in a careful way, the return on investment for the development and delivery of the COVID vaccine. The estimates are, not surprisingly, very large with ROIs between $60 and $475 dollars per dollar invested in vaccine development and delivery.*

**Response: Thank you for the positive feedback on the quality of our work.**

***B.*** *In the third paragraph the authors motivate the paper by citing surveys on vaccine skepticism. I'm not convinced this is a good way to motivate the paper. Simply put, this paper is not going to convince any vaccine skeptics, and they are not really the audience of the paper. I very much prefer the motivation to shape future policy about pandemic preparation. It may also be helpful to point out that this paper informs the debate about the pricing of the COVID vaccines. During the pandemic, there was discontent that companies that took public money could sell back the vaccine to the public at high prices. This paper shows that the surplus is so enormous that even at high prices there is substantial benefit to the public.*

**Response: Thank you for this suggestion to amend the framing of our paper. We believe the following changes have better contextualized our paper and highlight the importance of our research. We removed the lines about vaccine hesitancy and have chosen to focus our background on pandemic preparedness. The following changes were made:**

**Removed: “The ROI of COVID-19 vaccine development is an important unanswered question because the unprecedented effort in developing, manufacturing, and distributing COVID-19 vaccines to achieve high coverage is increasingly being questioned. A Pew Research survey found that a substantial minority of U.S. respondents were skeptical about the health benefits of COVID-19 vaccines, with notable levels of distrust in mRNA vaccines also observed in other countries.^6,7^”**

**Addition (Page 5, lines 18-27): “Additionally, the growing literature of economic evaluations on COVID-19 vaccines has primarily assessed the value of vaccines on the country level, limiting their analyses to national procurement and delivery costs. These analyses do not capture the full scale of investments that enabled vaccine availability in the first place. COVID-19 vaccines development was unique, because the vaccines’ rapid development required substantial, coordinated public investment across countries, including direct funding of research and development and Advanced Purchase Agreements (APAs) that de-risked private-sector production. These investments were made upstream of national vaccination programmes and generated global health and economic benefits that extend beyond any single country’s health system.^4^ As governments and global institutions consider how best to protect global populations in future pandemics, understanding the global economic returns to these upstream investments is critical.”**

**Addition (Page 6, lines 32-34): “Estimating the global ROI of COVID-19 vaccine development and delivery is therefore relevant and necessary to pandemic preparedness planning, including decisions about whether similar public investments should be completed for future global health emergencies.”**

**Addition (Page X, lines X-X): “Quantifying the ROI of COVID-19 vaccine development may also inform ongoing debates regarding vaccine pricing. During the pandemic, concerns were raised that vaccines developed with public funding were subsequently sold back to governments at higher prices and at variable prices between governments, raising questions about equity, affordability, and corporate profits.^7,8^ By estimating the total economic and health surplus generated by COVID-19 vaccines in monetary value relative to their development and distribution costs, conducting a ROI analysis may provide evidence that the health and economic benefits provided by the vaccines were worth their cost.”**

***C.*** *On page 4 I think the paper needs a clearer discussion of the difference between the welfarist and extra-welfarist approach. Specifically, the paper says the extra-welfarist approach broadens the evaluation. The reader may then be surprised that the broader extra-welfarist approach produces smaller benefits than the welfarist approach. This is a common result because VSL measures value a QALY more highly than extra-welfarist approach. The paper should clarify this issue.*

**Response: Thank you for this suggestion as it is interesting to note that while the extra-welfarist approach broadens the valuation, it yielded lower estimated benefits. To clarify this finding, we added this sentence to our discussion (page 17, lines 300-306): “The two approaches value different aspects of the vaccines, yet they both yield a net positive ROI, demonstrating that regardless of how the vaccines are valued (i.e., individuals’ preferences for reductions in mortality or the value society places on improvements in health). While the extra-welfarist approach broadens the evaluation by capturing the intrinsic value of health, it yielded lower estimated benefits than the welfarist approach because the monetary value assigned to a QALY, typically determined by societal WTP thresholds, is lower than the value individuals are willing to pay for a reduction in their mortality risk (i.e., VSL-based valuations).”**

**Additionally, Reviewer #2 made a similar suggestion to further describe the differences between the two approaches. We added the following to explain the differences between the approaches (page 7-8, lines 62-83): “The welfarist approach is the dominant view in welfare economics.^6^ Welfarist economics is based on maximizing individuals’ utility (the utility principle) so the ‘goodness’ of a situation is judged based on the utility derived from it.^11^ CBAs are generally considered to be a welfarist analysis as they judge a policy choice based purely on whether it increases aggregate individual utility, usually measured using the ‘value of a statistical life’ (VSL), which captures the amount of consumption that an individual is willing to give up to reduce their own mortality risk.^12^ VSLs are derived from stated (i.e. surveys) and revealed (i.e. data about how individuals act in real-world situations) preferences of individuals’ willingness-to-pay (WTP) for marginal reduction in their mortality risk.^12^ Hammitt^13^ has pointed out that that BCAs may not always be strictly welfarist since they involve analytical choices that correct individual preferences for various perceived biases, and whether it is welfarist or extra-welfarist depends on the justification adopted.**

**Furthermore, the welfarist approach does not admit broader societal values. These societal values may not align with individual preferences, particularly in health contexts. For instance, society may value good health and health improvements differently from individuals, particularly those with less income. To address these limitations, an extra-welfarist approach is adopted. Extra-welfarism differs in that it does not solely focus on individual’s utilities but assigns an intrinsic value to outcomes like ‘health’ and the inherent value of saving lives.^11^ In principle, this could give similar results to a welfarist approach if health is valued based on individual willingness-to-pay. Evaluations following extra-welfarist approaches include cost-effectiveness analyses (CEAs), which compare an interventions’ costs to its health outcomes, often use quality-adjusted life years (QALYs), which provide a non-monetary measure that combines both the quality and quantity of life, enabling the evaluation of health interventions based on the amount of health they produce,^14^ irrespective of monetary valuations of individual preferences.^14^”**

***D. i)*** *On pp. 6 I am confused by a few things in paragraph 4. For example, "it was estimated that COVID-19 vaccination generated and economic value of 27.2 trillion." I think this is a description of table 2. But table 2 lists the benefits as 35 trillion. I am having similar difficulty mapping the number in this paragraph for the extra-welfarist approach as well in table 3.* ***ii)*** *Also, should the benefits across the four income groups add up to the total benefit?* ***iii)*** *Finally, I don't understand how applying a discount rate increased the measured benefits from 27.2 trillion to 37.8 trillion (paragraph 4)*. **iv)** *In paragraph 5 on pp. 6, it would be more accurate to say the value of QALYs gained decreased instead of just QALYs gained decreased since the unit of measurement is dollars here.*

**Response:**

**Thank you for pointing out areas where we could improve the clarity of our work.**

**i)** *On pp. 6 I am confused by a few things in paragraph 4. For example, "it was estimated that COVID-19 vaccination generated and economic value of 27.2 trillion." I think this is a description of table 2. But table 2 lists the benefits as 35 trillion. I am having similar difficulty mapping the number in this paragraph for the extra-welfarist approach as well in table 3.*

**i) Table 2 and Table 3 report the totals as a percentage of GDP, rather than the totals in dollars, which is why there is a difference in the tables and the text. We believe that reporting the percentages helps contextualize our results better and can enable comparisons more easily. However, we can see why this may be confusing for readers, so we have now added the totals as percentages of GDP to the text to enhance understanding.**

**We added the following to our results section:**

**Page 15, lines 245-247: “Productivity costs saved from averted COVID-19 morbidity and mortality are estimated to be $564 billion (95% CrI 541–586) and 0.73% of global GDP (95% CrI 0.70 – 0.75), with LICs experiencing the greatest returns in dollars expressed as a percentage of GDPpc (Table 3).”**

**Page 15, lines 247-249: “COVID-19 vaccines were estimated to yield additional savings of $222 billion (95% CrI 213 – 229) in healthcare costs, which is an estimated 0.29% of global GDP (95% CrI 0.27 – 0.30).”**

**Page 15, lines 251-256: Under the welfarist approach, the benefits, including VSLs and productivity and healthcare costs averted, sum to an estimated total of $64.3 trillion (95% CrI 62.1 – 67.3), which is 82.7% (95% CI 80.0 – 86.7) of the global GDP. The extra-welfarist approach estimates a total of $6.44 trillion (95% CrI 6.22–6.75) gained in value from COVID-19 vaccination. After applying a discount rate, these overall benefits are estimated at $4.81 trillion (95% CrI 4.66 – 5.04). These extra-welfarist values correspond to 8.27% (95% CI 7.99 – 8.66) and 6.19% (95% CI 5.99 – 6.48), respectively.**

***ii)*** *Also, should the benefits across the four income groups add up to the total benefit?*

**ii) Thank you for raising this point. The short answer is that the reported median estimates for the four income groups do not necessarily sum to the reported global median estimate, and this is expected given that the uncertainty distributions are not symmetrical.**

**Specifically, all results are summarized using medians from simulated posterior draws. The global estimate is calculated by summing country-level outcomes within each simulation draw and then taking the median across draws. In contrast, income-group estimates are calculated by first summing outcomes across countries within each income group for each draw and then taking the median separately for each group.**

**Because the median of a sum is not generally equal to the sum of medians and our distributions are not symmetric, the sum of the income-group medians will generally differ from the global median. This reflects standard properties of summary statistics under uncertainty rather than any inconsistency in the underlying calculations.**

**We have clarified this in the manuscript to avoid potential confusion for readers by adding the following to the table legends where needed:**

**Tables 2, 3 and 4: *“Global and income-group estimates are reported as medians from simulated posterior draws. Income-group medians do not necessarily sum to the global median due to the non-additivity of medians under uncertainty.”***

***iii)*** *Finally, I don't understand how applying a discount rate increased the measured benefits from 27.2 trillion to 37.8 trillion (paragraph 4)*.

**iii) We changed our main analysis for the welfarist approach to be based on VSLs as per Robinson et al. (2019); however, we included VSLYs as a sensitivity analysis. As per Robinson et al. (2019), VSLYs are calculated using undiscounted life-years gained, therefore we no longer calculate nor report discounted VSLYs.**

**iv)** *In paragraph 5 on pp. 6, it would be more accurate to say the value of QALYs gained decreased instead of just QALYs gained decreased since the unit of measurement is dollars here.*

**iv) Thank you for the suggestion. We have implemented the suggested change, and the line now reads as follows (page 15, lines 240-241): “After applying a discount rate of 3%, the value of monetized QALYs gained decreased to $4.03 trillion (95% CrI 3.90 – 4.23).”**

***E.*** *The last paragraph on page six states that the ROI is well in excess of 1. This is such an understatement that I would consider revising it.*

**Response: Thank you for this suggestion. We agree that stating that the ROI is well in excess of 1 understates the huge return-on-investment. Accordingly, it now reads:**

**Page 15, lines 260-261: “Table 5 shows that all ROI estimates substantially exceed 1 (i.e., net positive returns) for COVID-19 vaccines globally for the first year of the vaccination programme, under both approaches.”**

**Page 16, lines 264-265: “Across all approaches, the 95% confidence intervals (CI) of the ROI far exceeded an ROI of 1 for the first year of the COVID-19 vaccination programme globally.”**

***F.*** *In the last paragraph on page 7 it is important to note that the benefits are high for high income countries because their VSL estimates are higher as well.*

**Response: Thank you for pointing out this important consideration. We added the following sentence to our discussion (page 20, lines 370-371): “Additionally, HICs gained higher economic value from averted COVID-19 mortality due to their high VSL estimates, which are based on GNIpc values.”**

***G.*** *In the discussion on page 8 of on equitable vaccine distribution. I would consider the Baker et al. (2021) point that investing in vaccine capacity to increase the supply of vaccines could help allowing for a more equitable distribution by limiting the incentive for vaccine nationalism.*

**Response: Thank you for providing this reference. We read and considered the important comments of Baker et al. (2021) and included the following sentences in our discussion (page 21, 403-407): “Additionally, investments into increasing vaccine capacity and supply globally in advance of pandemics may enable a more equitable distribution of vaccines by limiting the incentive for vaccine nationalism.^4^ By enhancing the global supply of vaccines and capacity to produce vaccines quickly in combination with limits on export bans and supply chain, will enable countries to vaccinate their populations sooner and share their vaccine supplies with our countries, especially LMICs.^4^”**

***H.*** *In summary, this is a nice paper which could be improved with a minor revision. In my view, the most urgent issue is* *to clarify the discrepancy between the text and table 2 and 3. And I hope the other suggestions are useful as well.*

**Response: Thank you very much for this positive feedback. We agree that your suggestions will help improve the quality and strength of our paper.**

References
Baker, Arthur, Esha Chaudhuri, and Michael Kremer. "Accelerating vaccinations." Finance Dev (2021): 12-16.

## Reviewer #5:

The paper estimates the return-on-investment (ROI) of COVID-19 vaccines globally by comparing vaccines’ development and manufacturing costs to the (monetized) benefits in terms of reductions in mortality, morbidity, and associated economic costs. The authors find that COVID-19 vaccines have a substantial ROI (larger than $60 for all simulation runs); the exact size depends, among other things, on the adopted framework (welfarist vs. extra-welfarist, discounted vs. undiscounted benefits).

The paper thoroughly shows that the massive investment into rapid development of COVID-19 vaccines paid off. I don’t think that the main finding of the paper will be qualitatively affected, but I have a few comments to increase the strength of the quantitative analysis.

A. The large ROI estimates are largely driven by the estimated numbers of deaths/cases averted. The paper relies on results by one modeling study (Watson et al. 2022). The authors mention that these estimates tend to be higher than results from regression models. However, it is difficult for the reader to understand the quality/reliability of these estimates compared to other results from the literature. A more in-depth discussion of the consensus/uncertainty around COVID-19 vaccination impacts would be helpful. For example:

***i)*** Are there other modeling studies that estimate the number of COVID-19 deaths/cases averted by vaccination? How do the Watson et al. estimates compare to the rest of the literature?

**Response: Yes, there is a substantial literature estimating COVID-19 cases and deaths averted by vaccination. However, almost all of such studies are national and regional rather than global. National and regional analyses can help to inform country-level vaccine procurement and resource allocation decisions. However, global analyses are needed to compare the total health and economic impact of vaccination with the investments made into their development.**

**There have been very few global studies assessing COVID-19 deaths. Some notable studies include those by Yang et al., Sevilla et al, and Moore et al. Watson et al. differs from other global studies in two key regards. First, they use a dynamic transmission model, which captures both direct and indirect effects of vaccination, while some of these COVID-19 modelling studies use *direct attribution approaches*, combining observed deaths over time with contemporaneous vaccine coverage and assumed vaccine effectiveness to estimate deaths averted through direct protection only. These methods generally do not model indirect effects via reduced transmission, do not capture feedback from reduced infection incidence, and do not represent how avoiding large waves of hospitalization can prevent health system saturation and associated increases in mortality. Second, Watson et al.’s estimates use excess mortality, compared to other studies which rely on reported COVID-19 deaths. Using reported deaths leads to systematic underestimation of vaccine impact, particularly in countries with incomplete vital registration and constrained health system reporting. Below we describe in detail how estimates in Watson et al. differ from the other global studies:**

**(Please find the table on the following page)**

| Study | Time frame | No. of countries included | Type of model | Estimation of COVID-19 deaths | Vaccine coverage assumption | Vaccine efficacy | Total COVID-19 deaths | Death estimate without vaccines | Deaths averted |
| --- | --- | --- | --- | --- | --- | --- | --- | --- | --- |
| Watson et al. | December 2020 – December 2021 | 185 | Age-structured dynamic transmission model fitted to country-level mortality and excess death data (compartmental SEIR framework) | Estimated COVID-19 deaths averted by comparing fitted epidemic trajectories with vaccination vs. counterfactual no-vaccination scenario; incorporated excess mortality adjustments | Vaccination rates were taken from publicly available data; assumed a vaccination strategy that targets those most at-risk first; vaccine coverage of 38.3% globally | Inferred based on the vaccine types predominately used in each country, with efficacy against disease ranging from 14%-96%, depending on vaccine type and dose number. | Reported deaths: 5.5 million  Excess mortality: 18 million | Reported deaths: 18.1 million  Excess mortality: 31.4 million | Reported deaths: 14 million  Excess mortality: 19.8 million |
| Yang et al. | December 2020 – September 2021 | 215 | Static Susceptible-Infectious- Recovered model | Used reported numbers of daily COVID-19-related cases and deaths to estimate the number of cases and deaths that would have occurred if there was no vaccination and found difference between this estimate and the reported number to obtain deaths averted | Number of individuals vaccinated was based on country-specific counts from Our World in Data (OWID)’s coronavirus pandemic data hub; estimated 2,663,000,000 people vaccinated globally | Used a vaccine effectiveness value against death of 86.3% globally | 4.66 million | Not reported | 620,500 |
| Moore et al. | Early 2020 – late 2021 | 152 | Simulated SARS-CoV-2 transmission and COVID-19 outcomes using deterministic compartmental model to assess alternative vaccine sharing strategies (alternative allocation); used age-dependent transmission and allowed for the effects of NPIs | COVID-19 deaths were estimated from excess mortality modelling using excess mortality estimates from the Institute for Health Metrics and Analysis | Based on past reports for daily vaccines administered in each country; 44.4% of global population was vaccinated with 2 doses by the end of 2021 | Based on efficacy characteristic similar to one or two doses of AstraZeneca vaccine, with efficacy ranging from 45% - 98% for infections, transmission, symptoms, and severe disease, as well as the number of doses | Mortality rate of 133.1 deaths per 100,000; 133.1 x 7.9 people = approx. 10.5 million deaths | Not estimated | 1.3 million (deaths averted under full sharing vs. observed roll-out) |
| Sevilla et al. | 2020 Q1 – 2021 Q4 | 148 | Regression analyses | Used reported deaths from Institute for Health Metrics and Evaluation COVID-19 Projections data, as well as other data points including vaccine doses administered, lag of new and cumulative infections, and NPIs, to estimate deaths averted using regression equations | Used data from Institute for Health Metrics and Evaluation (IHME) COVID-19 Projections (0.142 – 0.642 mean doses administered per capita per quarter) | Vaccine efficacy not explicitly considered, but assumed that more recent vaccination had stronger impacts | 15.4 million | 19.4 million | 4.1 million |

**From this comparison, we see that Watson et al. report higher estimates of deaths averted largely attributable to the incorporation of both direct protection and indirect effects through reduced transmission. Additionally, Watson et al. uses country-specific vaccine efficacy to reflect the predominate vaccine type used in each country. In contrast, the other studies use lower efficacy estimates, including Moore et al. which widely bases results on AstraZeneca vaccines, which are known to be less effective. While Moore et al. use a dynamic transmission model and consider excess mortality, this model reports on how many deaths could have been averted if there was greater sharing of vaccines globally. Moore et al. estimates that 1.3 million deaths could have been averted, in comparison with Watson et al. 14 million deaths. Another reason for this difference is that Watson et al. considered a vaccination strategy that targets the most at-risk first, resulting in greater health benefits from the vaccines. Each of these methodological differences compounds on each other leading to large differences in results.**

**Another global analysis, by Ioannidis et al., estimated approximately 2.5 million deaths averted globally over a longer time horizon, using a fundamentally different and highly conservative framework. Specifically, this analysis assumes infection fatality ratios roughly an order of magnitude lower than those estimated by Brazeau et al. which were used in Watson et al.’s model, assumes that only ~30% of the global population had received at least one vaccine dose prior to Omicron (with just 46% coverage even in the oldest age groups), applies vaccine effectiveness against death of 75%, and assumes independence between vaccination and prior infection, despite many high-income countries achieving high vaccine coverage before large post-vaccination waves occurred. Taken together, these assumptions compound multiplicatively. Applying their implied reductions (approximately 0.1 for IFR, 0.5 for vaccine coverage, 0.9 for vaccine effectiveness, and 0.5 for timing of vaccination relative to infection) to our reported-death estimate of 14.4 million deaths averted yields values on the order of a few hundred thousand deaths, consistent with their estimate. The divergence therefore reflects modelling assumptions rather than disagreement about the effectiveness of vaccines.**

**In response, we have added some of this comparison to the Discussion to place the results in contrast to the wider literature.**

**Page 22, lines 413-423: “Compared with other global studies,^3,26,37,38^ these estimates are notably higher due to methodological differences. Watson et al. model both direct and indirect effects of vaccination and relies on excess mortality estimates, rather than COVID-19 deaths alone. In contrast, other analyses use different modelling approaches with some using static models, and another using a similar dynamic model to Watson et al. Of those that use static models, these models are unable to capture transmission effects and therefore underestimate vaccine benefits. They also do not consider excess mortality in their analyses. On the other hand, Moore et al.^37^ used a dynamic model to evaluate how greater sharing of vaccines would have averted more deaths. However, Watson et al. reports a higher number of deaths averted likely due to the incorporation of vaccine prioritization amongst high-risk individuals and country-specific estimates of vaccine effectiveness. Watson et al. also includes a greater number of countries.”**

**References:**

**Brazeau NF, Verity R, Jenks S, et al. Estimating the COVID-19 infection fatality ratio accounting for seroreversion using statistical modelling. *Commun Med (Lond)*. 2022;2:54. Published 2022 May 19. doi:10.1038/s43856-022-00106-7**

**Sevilla JP, Burnes D, Knee JS, et al. The global health and economic value of COVID-19 vaccination. *BMJ Glob Health*. 2024;9(9):e015031. Published 2024 Sep 7. doi:10.1136/bmjgh-2024-015031**

**Yang J, Vaghela S, Yarnoff B, et al. Estimated global public health and economic impact of COVID-19 vaccines in the pre-omicron era using real-world empirical data. *Expert Rev Vaccines*. 2023;22(1):54-65. doi:10.1080/14760584.2023.2157817**

**Moore S, Hill EM, Dyson L, Tildesley MJ, Keeling MJ. Retrospectively modeling the effects of increased global vaccine sharing on the COVID-19 pandemic. *Nat Med*. 2022;28(11):2416-2423. doi:10.1038/s41591-022-02064-y**

**Ioannidis JPA, Pezzullo AM, Cristiano A, Boccia S. Global Estimates of Lives and Life-Years Saved by COVID-19 Vaccination During 2020-2024. *JAMA Health Forum*. 2025;6(7):e252223. Published 2025 Jul 3. doi:10.1001/jamahealthforum.2025.2223**

***ii)*** Is Sevilla et al. the only study estimating the benefits of COVID-19 vaccine with a regression model? That seems unlikely. Then, how do the estimates used in the paper compare with that literature?

**Response: Thank you for this comment. The body of literature published on COVID-19 vaccine models during the pandemic have been described in systematic reviews like those of Saadi et al. and Burch et al. There are two kinds of models: (i) mechanistic dynamic models, which explicitly model social contacts to capture indirect effects (e.g., herd immunity), and feedback between infection risk and population immunity, and (ii) static decision analytic models, which apply assumptions about vaccine coverage and efficacy directly to change the risk of individuals getting infected. By contrast, regression-based approaches, like those used by Sevilla et al., which use statistical methods to analyze risk factors, estimate transmission rates, and predict transmission rates, and predict spread by analyzing key variables, are relatively rare. The systematic reviews did not identify any regression models, suggesting that they are rare in the epidemiological modelling literature. While regression models are common in econometrics to assess the impact of different interventions such as policy changes like taxation and regulation, they are much less common in epidemiological studies, especially regarding infectious diseases. There may be other studies using regression-based analyses in the modelling literature that were not found in the previous reviews, but we did not have the time to conduct a full systematic review of both medical (e.g. PubMed) and economic (e.g. EconLit, RePEc, JSTOR) due to time constraints). Besides Sevilla et al., we identified only one other regression-based analysis in a report by the US government estimating the reduction in COVID-19 infections, hospitalizations, and deaths attributable to vaccination between December 2020 and July 2021. This report estimated that the vaccines reduced 213,349 COVID-19 deaths, which is much lower than those estimated by Watson et al, highlighting how estimates differ by methodologies (Holtkamp et al., 2021). This study was not found in the previous reviews (Saadi et al. and Burch et al.) because it did not meet their inclusion criteria (i.e., not a peer-reviewed mathematical modelling study).**

**References:**

**Saadi N, Chi YL, Ghosh S, et al. Models of COVID-19 vaccine prioritisation: a systematic literature search and narrative review. *BMC Med*. 2021;19(1):318. Published 2021 Dec 1. doi:10.1186/s12916-021-02190-3**

**Burch E, Khan SA, Stone J, et al. Early mathematical models of COVID-19 vaccination in high-income countries: a systematic review. *Public Health*. 2024;236:207-215. doi:10.1016/j.puhe.2024.07.029**

**Holtkamp, N., Kolbe, A., and Beleche, T. COVID-19 Vaccination Associated with Reductionsin COVID-19 Mortality and Morbidity in the United States, and an Approach to Valuing these Benefits. Washington, DC: Office of the Assistant Secretary for Planning and Evaluation, U.S. Department of Health and Human Services. December 2021.**

***iii)*** What about the country-specific confidence intervals derived from Watson et al? How big are they?

**Response: Watson et al. report country-specific uncertainty intervals for all estimates of lives saved, derived from uncertainty in model fit, epidemiological parameters, vaccine effectiveness, and mortality calibration. The absolute width of these intervals varies substantially by country, reflecting differences in epidemic size, data completeness, and signal-to-noise in reported mortality.**

**For countries with large epidemics, high vaccine coverage, and good mortality reporting, uncertainty intervals are relatively tight in proportional terms. In many high-income settings, 95% intervals typically span ±10–25% of the point estimate, even when the absolute number of lives saved is very large (often tens or hundreds of thousands). For example, countries with central estimates in the hundreds of thousands often have intervals differing by only a few tens of thousands.**

**In contrast, wider relative uncertainty is observed in countries with small epidemics, low reported mortality, limited vital registration, or sparse data, where point estimates are small and uncertainty intervals may span a factor of two or more. In a small number of settings with negligible reported mortality or vaccine uptake, the estimated number of lives saved is close to zero, with intervals correspondingly including zero.**

**We have added some of this to the Discussion as follows:**

**Page 20-21, lines 383-388: “In terms of uncertainty in public health estimates at the country-specific level, the intervals vary widely. However, they are proportionally narrow (95% intervals typically span ±10–25% of the central estimate) for countries with large epidemics and high vaccine coverage that contribute most to global mortality, and wider primarily in settings with small epidemics or limited mortality data, without materially affecting regional or global conclusions.”**

**B.** The authors adopt two different evaluation methods to estimate the benefits of vaccination. For the welfarist approach, they use country-specific VSLY estimates and multiply them by the number of life years gained. The proposed welfarist approach does not include productivity losses and healthcare costs because “these costs are assumed to be factored into individuals’ valuations of their preferences for reductions in mortality risk.” The welfarist approach also does not include the value of non-fatal cases. I find both assumptions restrictive:

i) Mortality gains are the most important source of benefits, but I don’t understand why the value of non-fatal cases should be included in the extra-welfarist approach but not in the welfarist one. The authors could derive a willingness-to-pay per QALY from the VSL estimates and multiply this value by the QALY gains as in the extra-welfarist approach.

**Response: Thank you for this comment. In the welfarist approach (as we describe it), benefits are valued based on individuals’ willingness to pay for reductions in mortality risk, which in our analysis was operationalized using value-of-statistical-life estimates. For COVID-19 vaccination, this reflects individuals’ stated and revealed preferences for avoiding premature death due to infection. We therefore did not include a separate valuation of non-fatal health outcomes in the welfarist approach.**

**While it is possible to derive an implied willingness-to-pay per QALY from VSL estimates and apply this to non-fatal health gains, doing so would impose assumptions that are not inherent to the welfarist framework. In particular, VSL-based valuations capture preferences over small changes in mortality risk and do not necessarily translate consistently to preferences over morbidity, quality-of-life changes, or temporary health states. As a result, extending VSL-derived values to non-fatal outcomes risks double counting or misrepresenting individual preferences. Consequently, Robinson et al. (2019) describes that using VSLYs to value QALYs is based on several simplifying assumptions that are not well supported in theory nor in empirical evidence.**

**In contrast, the extra-welfarist approach explicitly values both fatal and non-fatal health outcomes in terms of health gains (e.g., QALYs), reflecting its focus on health as the primary outcome of interest. Health gains are then valued using some external (i..e. “extra”) measure of society’s willingness-to-pay for health improvements, as evidenced for instance by the health opportunity costs implied by current levels of healthcare spending. This framework is therefore better suited to capturing morbidity effects alongside mortality reductions. To ensure comparability, we now include both productivity losses and healthcare costs averted in both approaches but restrict the monetized valuation of health outcomes to mortality risk in the welfarist analysis, consistent with its conceptual foundations.**

ii) It’s not clear to me that healthcare costs and productivity benefits are already captured in VSL estimates. Willingness to pay for an increase in survival may be affected by the out-of-pocket healthcare costs paid by the individual, but not all COVID-19-associated healthcare costs were paid out of pocket. In a standard BCA calculus, these third-party costs are typically added to the monetized value of deaths avoided. I don’t know if there are estimates of COVID-19 out-of-pocket healthcare costs, but a useful proxy could be WHO estimates of out-of-pocket expenditures. The same argument applies to productivity costs: in addition to a potential individual productivity loss (even though, in countries with social insurance, there may be no loss at all), there is an economy-wide loss that goes beyond the sum of individual ones (e.g., the GDP loss due to non-pharmaceutical interventions).

**Response: Thank you for this comment. Another reviewer made a similar comment and after consideration and reviewing the literature, we agree that productivity costs and healthcare utilization costs averted for those who survive should be included in the welfarist approach. We have now changed our methods and results to reflect this addition. Adding the productivity and healthcare costs to the welfarist perspective did not meaningfully change the results as they only contribute less than 1% to the overall welfarist benefits.**

***C.*** In the extra-welfarist approach, the authors determine the productivity loss accounting for absenteeism due to sickness and premature death. Although this is a standard methodology, I am not sure it is appropriate for the COVID case. In particular, it does not seem to fully capture the GDP loss due to economic shutdowns, supply chain disruptions, and other effects of non-pharmaceutical interventions. One of the biggest benefits of COVID vaccination was that it allowed the reopening of the economy and the associated economic benefits (which do not seem to be much correlated with the number of days of work that sick people lost). I would encourage the authors to re-evaluate how to best capture the economic benefits of COVID vaccination.

**Response: Thank you for this comment and for highlighting the broader macroeconomic effects of COVID-19 vaccination programmes. We agree that COVID-19 vaccination played a key role in enabling economic reopening and reducing the need for non-pharmaceutical interventions. We did not incorporate GDP-level effects into our extra-welfarist estimates for conceptual and methodological reasons.**

**Firstly, the epidemiological modelling by Watson et al. assumes that there are no changes to transmission apart from vaccine protection. If we assume that non-pharmaceutical interventions were relaxed following vaccination, then transmission of the infection would increase (compared to the current vaccination scenario in Watson et al), and hence the health impact of vaccination would be attenuated (even though economic impact would be greater).**

**Secondly, our analysis adopts an extra-welfarist framework that values health gains (averted mortality and morbidity, captured via QALYs) alongside productivity losses attributed to absenteeism due to sickness and premature death. Broader macroeconomic effects such as economic shutdowns and supply-chain disruptions were largely responses to population health risks and health system strain. As such, we aimed to assess the economic benefits related directly to the impact of COVID-19 vaccination on individuals’ health. We agree that there are other economic benefits of vaccination beyond the productivity losses averted. However, it is difficult to disentangle these macroeconomic gains from the direct productivity gains from averting sickness, since GDP aggregates embed labour supply and productivity components that overlap with productivity losses from illness and mortality.**

***D.*** The extra-welfarist approach leads to significantly lower estimates than the welfarist approach. One of the main differences comes from how the value of a (quality-adjusted) life year is estimated. In particular, the welfarist approach relies on willingness to pay estimates, while the extra-welfarist approach relies on health-opportunity costs to estimate the willingness to pay thresholds. An alternative method to estimate the thresholds is to rely on willingness to pay estimates (a demand side approach). In that case, there should be basically no difference between what the authors call the “welfarist” and “extra-welfarist” approaches (once all the benefits are adequately accounted for as previously explained). Therefore, the entire paper can be reframed in terms of the impact of willingness-to-pay thresholds on the COVID-19 ROI and how different methods for estimating the threshold affect the results. So, I think my question is: Why is the health-opportunity cost method the best approach to estimate the threshold? And why don’t the authors simply compare different ways of estimating the threshold rather than invoking differences between the welfarist and extra-welfarist approaches?

**Response: Thank you for this thought-provoking comment. We agree that a central driver of the difference between the welfarist and extra-welfarist results in our analysis is how health gains are valued.**

**Our decision to distinguish between welfarist and extra-welfarist approaches is to explore whether our overall conclusions are robust to the different normative foundations and corresponding evaluation frameworks of the two approaches. The welfarist approach aligns with a cost-benefit analysis framework, in which the efficiency of a health intervention is assessed based on individuals’ perceived welfare gains, typically captured through stated or revealed preferences for reductions in mortality risk.**

**In contrast, the extra-welfarist approach underpins cost-utility analyses, in which there is an external source of valuation for health gains apart from individual preferences, namely societal judgment. While in principle this external source could be derived from a variety of sources, including estimates of healthcare demand and willingness to pay for improved health, in our formulation (which is one of the most common), we take health opportunity costs in a particular country as a function of the revealed preference of that society for trading consumption for health on a national level. Our intent in applying these two frameworks is therefore not simply to compare alternative mathematical formulations for converting health into monetary terms, but to explore whether different normative approaches (welfarism versus extra-welfarism, cost-benefit versus cost-utility) still yield a net positive ROI to appeal to approaches and methodologies used by different policy makers (e.g. Ministry of Health, Ministry of Finance, private sector, global donors) in different countries and their intentions for health budgets and investments into public health interventions. While it is indeed still methodologically correct to conduct a cost-utility analysis using individual willingness-to-pay as the cost per QALY threshold, this is both less commonly applied and also would give largely the same results as the welfarist analysis as the reviewer correctly points out, so would be less informative as a sensitivity analysis. We have added the following text to explain the welfarist and extra-welfarist approaches to clarify this distinction and the utility of both approaches to value vaccines.**

**Addition (page 7-8, lines 62-83): “The welfarist approach is the dominant view in welfare economics.^6^ Welfarist economics is based on maximizing individuals’ utility (the utility principle) so the ‘goodness’ of a situation is judged based on the utility derived from it.^11^ CBAs are generally considered to be a welfarist analysis as they judge a policy choice based purely on whether it increases aggregate individual utility, usually measured using the ‘value of a statistical life’ (VSL), which captures the amount of consumption that an individual is willing to give up to reduce their own mortality risk.^12^ VSLs are derived from stated (i.e. surveys) and revealed (i.e. data about how individuals act in real-world situations) preferences of individuals’ willingness-to-pay (WTP) for marginal reduction in their mortality risk.^12^ Hammitt^13^ has pointed out that that BCAs may not always be strictly welfarist since they involve analytical choices that correct individual preferences for various perceived biases, and whether it is welfarist or extra-welfarist depends on the justification adopted.**

**Furthermore, the welfarist approach does not admit broader societal values. These societal values may not align with individual preferences, particularly in health contexts. For instance, society may value good health and health improvements differently from individuals, particularly those with less income. To address these limitations, an extra-welfarist approach is adopted. Extra-welfarism differs in that it does not solely focus on individual’s utilities but assigns an intrinsic value to outcomes like ‘health’ and the inherent value of saving lives^10^. In principle, this could give similar results to a welfarist approach if health is valued based on individual willingness-to-pay. Evaluations following extra-welfarist approaches include cost-effectiveness analyses (CEAs), which compare an interventions’ costs to its health outcomes, often use quality-adjusted life years (QALYs), which provide a non-monetary measure that combines both the quality and quantity of life, enabling the evaluation of health interventions based on the amount of health they produce,^12^ irrespective of monetary valuations of individual preferences.^12^”**

***E.*** About the difference between the welfarist and extra-welfarist approaches, I think it would be useful if the authors included a definition of what they mean with the two terms. There is a debate in the BCA literature whether the Kaldor-Hicks criterion (which is implicitly adopted in the paper) has normative value (i.e., is a standard BCA calculus that compares total monetary costs and total monetary benefits really promoting welfare). Thus, I am not sure I would call standard BCA a “welfarist” approach (which goes back to my comment about defining the terminology).

a. See, e.g., James K. Hammitt, [Positive versus Normative Justifications for Benefit-Cost Analysis: Implications for Interpretation and Policy](https://www.tse-fr.eu/fr/articles/positive-versus-normative-justifications-benefit-cost-analysis-implications-interpretation-and), *Review of Environmental Economics and Policy*, vol. 7, n° 2, 2013, p. 199–218.

**Thank you for the helpful reference, which has been enlightening, and which we agree makes many good points.**

**BCA evaluates alternative policy choices based on aggregating their benefits and costs using individual willingness to pay. This aggregation is generally justified by appeal to the Kaldor-Hicks compensation test. This treats individuals as the best judge of their own welfare (consumer sovereignty), so in the conventional understand BCA is welfarist.**

**However, we take the point that passing the Kaldor-Hicks compensation test guarantees that social welfare actually increases, given the various issues mentioned in the Hammitt et al. Paper. In that sense, BCA may actually be extra-welfarist, in the sense that the analyst often corrects for individuals’ preference biases rather than respecting preference satisfaction as the sole aim of the analysis. However, I think both Hammitt and the reviewer would agree with us that BCA is still welfarist in some narrow meanings e.g. as a positive/pragmatic exercise in aggregating individuals’ preferences assuming they are well-informed.**

**We do not have the space to go into a discussion of the meaning of BCA in the level of depth of Hammitt et al, but we have revised our language to be more precise:**

**Page 7, lines 62-82): “The welfarist approach is the dominant view in welfare economics.^6^ Welfarist economics is based on maximizing individuals’ utility (the utility principle) so the ‘goodness’ of a situation is judged based on the utility derived from it.^11^ CBAs are generally considered to be a welfarist analysis as they judge a policy choice based purely on whether it increases aggregate individual utility, usually measured using the ‘value of a statistical life’ (VSL), which captures the amount of consumption that an individual is willing to give up to reduce their own mortality risk.^12^ VSLs are derived from stated (i.e. surveys) and revealed (i.e. data about how individuals act in real-world situations) preferences of individuals’ willingness-to-pay (WTP) for marginal reduction in their mortality risk.^12^ Hammitt^13^ has pointed out that that BCAs may not always be strictly welfarist since they involve analytical choices that correct individual preferences for various perceived biases, and whether it is welfarist or extra-welfarist depends on the justification adopted.**

***F.*** Most importantly, summing income-specific VSLYs across countries without acknowledging differences in the marginal value of money leads to an overestimation of the vaccination benefits in high-income countries and an underestimation of its benefits in low-income countries. I think a discussion of this issues should be included in the paper, especially given that most mortality/morbidity benefits occur in high-income countries. The authors could also include an additional sensitivity analysis where all life years are equally valued with a global VSLY. Or adjust country-specific VSLY estimates with weights as recommended, e.g., in the UK Green Book.

**Response: Thank you for this important suggestion. We have accordingly conducted an additional sensitivity analysis, where we calculated a population-weighted global mean of the value of a life year to calculate our VSLY estimates across income groups and subsequently the ROI estimate. We found that while using this single value, our ROI estimate decreased, it is still well in excess of 1. Please find the following associated changes in our manuscript:**

**Methods (page 13, lines 196-202): “In addition to the country-specific VSLYs, we also estimated an ROI using a uniform global VLY to determine VSLYs. This VLY was derived from a population-weighted mean VLY from the country-specific VLYs included in the analysis.** **This additional analysis was conducted as a contrasting scenario to the base case with country-specific VSLYs, acknowledging that differences in the marginal value of money across country income groups could affect the ROI estimates and the distribution of benefits across income settings, since an equivalent increase in income may gain more utility in a lower-income country.”**

**Results (page 16, lines 276-280): “Using a uniform VSLY value of $40,946.05 across countries, the ROI estimate from the welfarist perspective was still well in-excess of 1. VSLYs gained per income group using a single VLY value can be found in Supplementary Table 5 and associated ROI estimates in Table 5.”**

**Discussion (page 18, lines 320-325): “To account for differences in the marginal value of money across income groups, we also applied a uniform global VLY. While this approach produced similar trends in the absolute value of monetized benefits, expressing the results on a per-person vaccinated basis as a percentage of GDPpc revealed that low-income countries experienced the largest relative gains. This finding highlights the comparatively greater economic benefits gained by vaccination in lower income settings when assessed relative to available economic resources.”**

***G.*** The discounted welfarist ROI is larger than the undiscounted one. At first sight, this sounds odd. From my understanding, this is due to the fact that zero discounting affects both the total number of life years gained *and* the VSLY. Is this a standard approach? I may be wrong, but I don’t think it is (or, at least, not in the literature that I know). First of all, I think this computation should be explained in the main text. Second, I am not sure what is the rationale here. If VSL derives from individuals’ willingness to trade changes in mortality with changes in income/wealth, then it should account for individuals’ (rational) preferences, including discounting. In contrast, whether each death or life year should count the same is a normative issue, which may have nothing to do with individuals’ preferences (unless you assume that individuals’ preferences dictate normative preferences). I would recommend the authors to spell out what their discounted and undiscounted approaches represent (rather than just saying that this is what the WHO recommends).

**Response: Thank you for this comment. After reviewing Robinson et al. (2019), we changed our main welfarist analysis to use VSLs to monetize the number of deaths averted and used VSLY for a sensitivity analysis. In VSLY calculations, Robinson recommends dividing VSL by the undiscounted future life expectancy of each country and then multiplying that value by the number of undiscounted life years gained from COVID-19 vaccines. As such, this sensitivity analysis only uses undiscounted life years, and we have removed the discounted life years estimates from our analysis. The details of this sensitivity analysis can be found in an addition to our manuscript (page 12, lines 192-196):**

**“Another analysis was conducted within the welfarist perspective to determine the monetary value of lives saved by applying the value of statistical life years (VSLY), as per Robinson et al. (2019).^18^ Country-specific VSLY values were obtained by dividing the VSL by the undiscounted future life expectancy of each country to get the value of a life year (VLY). This VLY is then multiplied by the number of undiscounted life years gained from COVID-19 vaccination.**

**Further, in our methods, we describe that our discounted approaches are used to reflect time preferences for gains in current health benefits. In our methods it reads (page 10, lines 122-124): “LYs gained were determined by multiplying age group-specific average life expectancies for each country by the number of deaths averted, with a 3% discount rate applied to reflect time preferences for current health benefits.”**

***H.*** The authors assume that the income elasticity of VSL is equal to 1. Robinson et al. suggests using higher income elasticities for lower-income countries (e.g., 1.5) on the ground that income elasticity seems to vary by income group. This would be an interesting sensitivity analysis.

**Response: We appreciate this positive suggestion and included a sensitivity analysis where the income elasticity for LMICs and LICs was increased to 1.5. We found that this slightly decreased our ROI estimate for the welfarist perspective, but it did not introduce any meaningful changes, as the ROI estimate is still well in excess of $1.**

**To reflect this additional analysis, we added the following:**

**Page 12, lines 202-204: “Lastly, we also explored how increasing income elasticity to 1.5 for LMICs and LICs affects the welfarist ROI estimates, as per Robinson et al. (2019)’s recommendations.^18^”**

**Page 16, lines 279-280: “Additionally, when the income elasticity of LMICs and LICs was increased to 1.5, there was a slight decrease in the welfarist ROI estimate.”**

**Page 18, lines 325-328: “Lastly, increasing the income elasticity to 1.5 for LMICs and LICs resulted in a modest reduction in ROI estimates. This decrease reflects that the value of VSLs in LMICs and LICs is lower than in HICs, given that those in lower income setting must devote larger shares of their incomes to necessary and urgent expenses.^18^”**

***I.*** The VSL approach adopted in the paper implicitly assumes that COVID was a “small risk” (and thus one can simply multiply VSLY by the number of LYs to determine the overall impact). There was some debate about whether COVID-19 can be considered a small risk or whether a different methodology should be applied. Not sure where the debate landed, but it would be interesting to include such a discussion in the paper.

b. See, e.g., Hammitt JK. Valuing mortality risk in the time of COVID-19. J Risk Uncertain. 2020;61(2):129-154. doi: 10.1007/s11166-020-09338-1.

**Response: Thank you for providing this noteworthy point of discussion. This debate around using VSL to evaluate COVID-19 policies is pertinent to this paper and important for contextualizing our results. To highlight this debate, we added the following sentences (page 23, lines 445-458): “One concern is that the VSL represents the marginal rate of substitution between wealth and mortality risk, but COVID-19 mortality risk in 2021 was so large that the rate is no longer marginal (meaning that people could not afford the wealth sacrifice needed to eliminate this risk, given commonly used VSL estimates (e.g., approximately USD 10 million in the United States). In partial mitigation, it should be noted that vaccination coverage levels in 2021 fell far short of averting all mortality associated with COVID-19. Secondly, vaccination disproportionately averted deaths among older adults who might have a lower VSL due to their shorter remaining life expectancy. This would decrease the estimated ROI of vaccination, as demonstrated by our sensitivity analysis using VSLYs, although the ROI remains high even when using VSLYs. Thirdly, VSL reflects individuals’ preferences over risk, and risks that are perceived as more uncertain, salient, or dreadful, such as those associated with COVID-19, particularly early in the pandemic, may command higher willingness to pay for risk reductions. Under such circumstances, standard VSL values may understate the welfarist benefits of vaccination. Taken together, these considerations highlight uncertainty around the appropriate valuation of COVID-19 mortality risk.”**

***J.*** I don’t understand how to read Supplementary Table 1. In particular, what is the first column? And what do all those numbers refer to? Different studies?

**Response: Thank you for these clarifying questions. We have edited Supplementary Table 1 to be more easily interpretable. Now, the first column corresponds to the type of cost. The numbers refer to the monetary value of the cost, and the subsequent columns include the year the currency and year that the costs were incurred in and then the reference of where we obtained these values from.**

***K.*** It’s not very easy to appreciate the heterogeneity of estimates in Figure 1. Perhaps the authors could use a different set of colors (e.g., going from green to red).

**Response: We have adjusted the colors in Figure 1 to have more contrast, which we hope will improve readers’ interpretation of the figure.**

***L.*** As a reader, I would also like to have a better sense of the parameter estimates used in the analysis. The authors report the sources of the estimates, but I don’t see any numbers. I understand that there are many parameters and many countries, but it would be useful to add a few tables with summary statistics (e.g., what is the US VSL baseline value used and its range in sensitivity analysis? How many averted deaths/hospitalizations (mean and 95% CI) by income group or region? etc.)

**Response: Thank you for this suggestion. We added some summary statistics to Table 1 to facilitate readers’ understanding of our analysis inputs. Supplemental Tables 2 and 3 include the public health inputs to our model, including life years gained, and hospitalizations and infections averted, respectively.**
