# Supplementary material for: Global return-on-investment of COVID-19 vaccines in the first year of the vaccination programme: A model-based economic evaluation
Source: PLoS Med. 2026 Nov 5;23(11):e1004772. doi: 10.1371/journal.pmed.1004772 (PMC13739145; doi:10.1371/journal.pmed.1004772)
Supplement: Supplementary file 6 [file pmed.1004772.s008.docx]

## ***Reviewer #1:***

### ***Comments:***

***A.*** *I congratulate the author's to the great revision of their paper. This was an excellent rebuttal letter to read and I thank the authors for answering each reviewer in such detail. I only have two minor comments and I believe, if fixed, this work will be of great interest to the readers of PLOS Medicine.*

**Response: Thank you for the kind comment and feedback on our paper.**

***B.*** *L 73: "Furthermore, the welfarist approach does not admit broader societal values. " I do think that could be better phrased, because compared to extra-welfarism, welfarism is the broader framework as it takes individual preferences across all domains of life into account. I think it is more that the welfarist approach does not accommodate values that cannot be expressed through individual utility. Hence, I'd consider rephrasing the sentence.*

**Response: Thank you for this suggestion. We believe the changes we implemented in response to Reviewer #4’s comments help address this inaccuracy. Specifically, we changed our two approaches to be the i) cost-benefit (CBA) analysis and ii) cost-effectiveness analysis (CEA). While we do mention that these are applications drawn from welfarist and extra-welfarist frameworks, we no longer make general statements about welfarism and extra-welfarism that strictly only apply to our particular approaches.**

**The main changes can be found in the following paragraphs:**

**Page 7-8, lines 57-84: The ROI of COVID-19 vaccination over the first year of the vaccination programme globally was determined by estimating the value of its public health outcomes and then comparing them to the investments made into COVID-19 vaccine development and delivery using two standard approaches: a cost-benefit analysis (CBA) and a cost-effectiveness analysis (CEA).**

**In the CBA, health benefits are expressed in monetary terms based on individuals’ willingness-to-pay (WTP) for them. This reflects the maximum amount of consumption that an individual is willing to forgo for the benefit. In the CBA, vaccine-attributed reductions in mortality are valued using estimates of individuals’ WTP for small reductions in mortality risk, aggregated to derive the value of a statistical life (VSL). VSL estimates are typically derived from stated (i.e. surveys) and revealed (i.e. data about real-world risk-trade-off situations) preferences.^11^ Our CBA framework also incorporates the monetary value of other benefits such as morbidity reductions and productivity gains.**

**CBA is commonly associated with a welfarist framework, which evaluates the desirability of an option according to its effect on individual utility.^12^ However, Hammitt^13^ notes that CBAs are not always strictly welfarist, since they involve analytical choices that correct individual preferences for various perceived biases. Depending on their justification, these corrections reflect either welfarist or extra-welfarist principles. Moreover, alternative formulations of welfarism from the CBA approach we used aggregate individual preferences differently; for example, some incorporate distributive concerns.^14^**

**The CEA typically evaluates interventions by comparing their incremental costs and health gains separately. The ratio of the two (known as the incremental cost-effectiveness ratio or ICER) is then compared with a cost-effectiveness threshold representing the opportunity cost of additional health gains. These opportunity costs are the value of health foregone elsewhere in the health system when resources are allocated to the next best alternative intervention or health programme.^15^ Rather than monetizing health gains directly, CEAs use non-monetary health measures such as quality-adjusted life years (QALYs), which combine both the quality and quantity of life into a single metric.^16^ It is therefore considered an extra-welfarist approach, since it differs from welfarism in that it does not assign value solely based on individual preferences but assigns an intrinsic value to health.^12^ In our analysis, we adapt this approach to produce an ROI by estimating QALYs gained in monetary terms using estimates of the health opportunity costs of healthcare spending by country.^17^**

**Page 11, lines 159-162: Overall, the health and economic benefits considered in the CBA included averted mortality valued by VSLs and reduced third-party healthcare and productivity costs due to averted mortality and morbidity; while the CEA’s health and economic benefits included averted mortality and morbidity measured using QALYs and reduced third-party healthcare and productivity costs due to averted mortality and morbidity.**

**We have also updated the language elsewhere in the manuscript to be consistent with the terminology used in our methods.**

***C.*** *I am also aware that reference 32 (Banhawi et. al 2025) was now published in two journals. The quantitative results the author's are referring two is published in:*[*https://pubmed.ncbi.nlm.nih.gov/41410417/*](https://pubmed.ncbi.nlm.nih.gov/41410417/) *and the targeted review was published in:* [*https://pubmed.ncbi.nlm.nih.gov/39203978/*](https://pubmed.ncbi.nlm.nih.gov/39203978/)*.*

*Very best wishes.*

**Response: Thank you for pointing this out to us. We have accordingly changed our reference to cite the quantitative results by Chowdhury et al. (2025).**

***Reviewer #3:***

### ***Comments:***

***A.*** *The authors have comprehensively responded to my comments, I am happy to accept.*

**Response: Thank you very much for the consideration of our paper and our response to your comments. We believe they added great value to the quality and content of our paper.**

***Reviewer #4:***

### ***Comments:***

A. *I thank the authors for their thoughtful revisions and detailed responses to my comments. The paper addresses an important and timely question, and the main finding — that COVID-19 vaccine development yielded substantial returns — is convincing and robust to a range of assumptions. Several of my original comments have been well addressed, including the addition of sensitivity analyses for income elasticity and uniform VSLY, the discussion of non-marginal risk, and the more detailed reporting of parameter estimates and results by income group. However, some comments have not been fully addressed, and the revision of the paragraph on the welfarist and extra-welfarist frameworks has introduced new inaccuracies. I outline my remaining concerns below.*

**Response: Thank you for the positive feedback on our revisions. We are grateful for the opportunity to further strengthen our work by implementing your following suggestions and have made substantial changes to the terminology used in response to the concerns raised.**

B. *Definition of welfarist/extra-welfarist. I appreciate that the authors now include a paragraph that defines the difference between welfarist and extra-welfarist approaches. However, I don't think the revision fully addresses my original comment, and the added material introduces several inaccuracies.*

*I think the main issue is that the authors conflate welfarism with something narrower (unweighted CBA or the Kaldor Hicks compensation criterion) and then present extra-welfarism as the solution to limitations that welfarism itself does not necessarily have. Whether the unweighted CBA framework used in the paper constitutes a "welfarist" evaluation is contested in the literature, and in a study of global vaccine ROI, where both VSL and the distribution of benefits vary enormously across income levels, this is not merely a terminological issue.*

**Response: Thank you for highlighting the issues with how we conflated welfarism with a narrower unweighted CBA framework, and then presented extra-welfarism as a solution to limitations that welfarism does not necessarily have. We recognise that even though unweighted CBA is the most well-known application of welfarism, it was lazy to use the two interchangeably. We now describe one of the two approaches used in the paper as the “Cost-benefit analysis (CBA) approach” rather than the “welfarist approach”, and the alternative approach as the “Cost-effectiveness analysis (CEA) approach” rather than the “extra-welfarist approach”. We have also added text explaining (with references) that the CBA approach is one application of welfarism, but there are other welfarist approaches that do not require the same assumptions as CBA (see response to bullet points below).**

*Several specific claims in the revised paragraph reflect this conflation:*

- *“The welfarist approach does not admit broader societal values.” The specific version used in the paper may not incorporate societal values, but that’s not true for a more general approach – see all the literature on the use of social welfare functions in policy evaluation (e.g., the work by Matthew Adler, Marc Fleurbaey, etc.)*

**Response: Thank you for introducing this important nuance; we agree that we have been sloppy in our terminology. We acknowledge that the use of a social welfare function allows incorporation of weights that reflect social values into an evaluation and have revised our description of this approach.**

- *“The welfarist approach is the dominant view in welfare economics.” Welfare economics is much broader than what the authors consider “the welfarist approach”.*

**Response: Thank you very much for this important point. We agree; our intention was to position welfare economics as the overarching field of economics that encompasses both welfarist and extra-welfarist frameworks. The inclusion of this economic terminology was a response to several reviewers requesting more methodological detail. However, in retrospect, this level of pedagogical detail is not really necessary to convey our methods, so we have removed this sentence.**

- *CBAs are "usually operationalised using VSL." VSL is used for the mortality-risk component of a CBA, but CBAs can encompass many types of benefits and costs — time savings, morbidity, productivity, environmental quality, etc. The paragraph makes it sound like VSL is the CBA methodology, when it's one input.*

**Response: Thank you for highlighting this important distinction in describing our methods. We have now revised our writing to make it clear that VSL is one approach to valuing reductions in mortality risk that we have used, and that we also include other benefits such as morbidity reductions and productivity gains.**

- *VSL "captures the amount of consumption that an individual is willing to give up to reduce their own mortality risk." VSL is the marginal rate of substitution between wealth (or income) and mortality risk — it's derived from WTP for small risk reductions, then scaled up.*

**Response: Thank you providing this critical feedback on how we describe VSLs. We have now amended the text accordingly.**

- *The authors also write that “In principle, this could give similar results to a welfarist approach if health is valued based on individual willingness-to-pay.” This is logically correct, but in a paragraph that is building a case for why both approaches are used, it undercuts the argument for why extra-welfarism is needed.*

**Response: Thank you for this comment. We added this text at the request of another reviewer who noted that our two approaches were mathematically formulated in a way that they were almost equivalent if we used a cost-effectiveness threshold corresponding to individual willingness-to-pay to avoid mortality risks. We suspect you would argue that the two approaches are conceptually very different even if mathematically they are similar in our formulation, and we agree with you but did not want to begrudge the point with the other reviewer. However, we think that with the improvements in our terminology based on your other comments, the conceptual distinction between the two approaches is clearer, and we should indeed drop this sentence.**

*I would encourage the authors to define their two approaches operationally, stating what each includes, how benefits are valued, and why — as the original version of this paragraph did. If the authors wish to additionally frame these approaches as “welfarist” and “extra-welfarist”, they should provide clear definitions of what they mean by these terms in the context of this study, rather than characterizing the frameworks in general terms.*

**Response: This is a good idea. As mentioned, we now called our approaches the “CBA approach” and “CEA approach”, and while we do mention that these are applications drawn from welfarist and extra-welfarist frameworks, we no longer make general statements about welfarism and extra-welfarism that strictly only apply to our particular approaches.**

**Overall, thank you very much for this critical review of the health economic theory underpinning our paper. Part of the challenge has been addressing multiple reviewer comments about the health economic evaluation terminology that led in different directions. Using your feedback, we believe we have resolved the weaknesses in our terminology. We summarise the changes made here:**

**Page 7-8, lines 57-84: The ROI of COVID-19 vaccination over the first year of the vaccination programme globally was determined by estimating the value of its public health outcomes and then comparing them to the investments made into COVID-19 vaccine development and delivery using two standard approaches: a cost-benefit analysis (CBA) and a cost-effectiveness analysis (CEA).**

**In the CBA, health benefits are expressed in monetary terms based on individuals’ willingness-to-pay (WTP) for them. This reflects the maximum amount of consumption that an individual is willing to forgo for the benefit. In the CBA, vaccine-attributed reductions in mortality are valued using estimates of individuals’ WTP for small reductions in mortality risk, aggregated to derive the value of a statistical life (VSL). VSL estimates are typically derived from stated (i.e. surveys) and revealed (i.e. data about real-world risk-trade-off situations) preferences.^11^ Our CBA framework also incorporates the monetary value of other benefits such as morbidity reductions and productivity gains.**

**CBA is commonly associated with a welfarist framework, which evaluates the desirability of an option according to its effect on individual utility.^12^ However, Hammitt^13^ notes that CBAs are not always strictly welfarist, since they involve analytical choices that correct individual preferences for various perceived biases. Depending on their justification, these corrections reflect either welfarist or extra-welfarist principles. Moreover, alternative formulations of welfarism from the CBA approach we used aggregate individual preferences differently; for example, some incorporate distributive concerns.^14^**

**The CEA typically evaluates interventions by comparing their incremental costs and health gains separately. The ratio of the two (known as the incremental cost-effectiveness ratio or ICER) is then compared with a cost-effectiveness threshold representing the opportunity cost of additional health gains. These opportunity costs are the value of health foregone elsewhere in the health system when resources are allocated to the next best alternative intervention or health programme.^15^ Rather than monetizing health gains directly, CEAs use non-monetary health measures such as quality-adjusted life years (QALYs), which combine both the quality and quantity of life into a single metric.^16^ It is therefore considered an extra-welfarist approach, since it differs from welfarism in that it does not assign value solely based on individual preferences but assigns an intrinsic value to health.^12^ In our analysis, we adapt this approach to produce an ROI by estimating QALYs gained in monetary terms using estimates of the health opportunity costs of healthcare spending by country.^17^**

**Page 11, lines 159-162: Overall, the health and economic benefits considered in the CBA included averted mortality valued by VSLs and reduced third-party healthcare and productivity costs due to averted mortality and morbidity; while the CEA’s health and economic benefits included averted mortality and morbidity measured using QALYs and reduced third-party healthcare and productivity costs due to averted mortality and morbidity.**

**We have also updated the language elsewhere in the manuscript to be consistent with the terminology used in our methods.**

C. *Double counting in the welfarist case. One of my original comments was about the absence of third-party costs in the CBA calculation. In this revision, the authors have added healthcare costs and productivity losses to both the “extra-welfarist” and CBA approaches. But the VSL is already supposed to include costs borne by the individual, thus leading to double counting. What I call third-party costs are healthcare costs borne by the healthcare system/insurance provider and productivity losses borne by other people (e.g., those who are not sick but required to miss work because of economic lockdowns). In the sensitivity analysis, the authors show that this issue is quantitatively minimal (excluding the added costs has not substantial effect on the ROI, even though this is because they underestimate the productivity loss – see my comment below on GDP loss); however, it is conceptually an important issue and the choice made by the authors is not justified or discussed.*

**Response: Thank you for pointing this out. After the previous round of reviews, we included both productivity costs and healthcare costs in the CBA approach, in addition to VSL, to account for the costs borne by third-party individuals, such as employers and health systems, respectively. For productivity costs, the friction cost approach estimates the output losses borne by employers during the period when a sick worker cannot work due to illness or deceased worker is replaced. These are costs that presumably fall outside an individual’s marginal rate of substitution between wealth and mortality risk (since they do not alter the individual’s health or consumption) and are therefore not captured by VSL estimates. For healthcare costs, we assumed that these are borne by the health system or insurer and similarly do not overlap with VSL-based valuations. We acknowledge that individuals who were sick with COVID-19 likely incurred out-of-pocket costs, but for the purposes of this valuation, we assume that they are included in the VSL estimates. As you point out, our sensitivity analysis demonstrates that excluding these costs has negligible impact on the ROI estimates, so we believe that our conclusions are robust to this potential double-counting. These choices are discussed in here…**

**Page 10-11, lines 141-155: Economic benefits generated during the first year of the COVID-19 vaccination programme were assessed from a societal perspective in both the CBA and CEA, by estimating both averted productivity losses and healthcare costs. Productivity costs averted due to reduced COVID-19 related absenteeism and deaths were calculated using the friction cost (FC) approach. This approach was chosen over the human capital approach to be conservative in estimating vaccine benefits; the FC approach assumes that sick or deceased workers are replaceable and only considers productivity costs incurred during the period when the position is unfilled (friction period). These costs represent the output losses borne by employers during the friction period, presumably falling outside an individual’s marginal rate of substitution between wealth and mortality risk (since they do not alter the individual’s health or consumption) and are therefore not captured by VSL estimates. Healthcare costs averted were estimated by multiplying the number of hospitalizations averted by costs per hospitalization. These healthcare costs are assumed to be borne by health systems or insurers rather than incurred out-of-pocket by individuals and thus do not overlap with VSL-based valuations. We acknowledge that individuals sick with COVID-19 likely incurred some out-of-pocket costs, but for the purposes of this analysis we assume these are captured within VSL estimates.**

D. *Value of nonfatal outcomes in the welfarist case. In their response, the authors present the omission of morbidity from the welfarist approach as if it is simply a feature of the method rather than a limitation or a choice that could have been made differently. In the paper, there is no discussion of why non-fatal effects are excluded from the welfarist approach, and no acknowledgment that this understates welfarist benefits.*

**Response: Thank you for this comment. We acknowledge that we have been using welfarism as a shorthand for “CBA with all health benefits monetized using VSL”, which as you have pointed out, is conceptually lazy. We have changed our description of this approach to be the “CBA using VSL” approach, which we describe as a welfarist approach but no longer make sweeping statements about welfarism which only apply to our particular approach.**

E. *GDP loss from lockdowns/economic reopening benefits. The productivity costs still only capture absenteeism and death (lines 136–139). The massive economic benefit of vaccines enabling reopening is still missing from the analysis. I understand that there are methodological limitations here linked to the assumptions in the underlying transmission model. However, this issue should at least be acknowledged in the limitation section. The paper's framing — "global return-on-investment," whether the investment "paid off" — implies a comprehensive assessment of whether vaccine development was worth it. If what may be the single largest economic benefit of vaccination is excluded, the reader should understand that the ROI estimates are likely to be conservative for this reason. Neither the welfarist nor the extra-welfarist approach are incompatible with including the GDP loss, as the authors seem to imply in their response.*

**Response: Thank you for this comment. We completely agree that the economic benefits of reopening were huge, and that vaccination played a very large role in enabling this. Hence our inability to capture a scenario where vaccination allowed the relaxation of restrictions is a major limitation, and we have now acknowledged this explicitly in the Discussion.**

**Having said that, the actual benefit of vaccination in this respect is actually quite complex. This is because the economic benefits of reopening were partly offset by the additional transmission associated with increased social and economic interaction following the opening. Hence the difference in economic output before and after vaccine rollout cannot simply be added to the health benefits of vaccination – this would overestimate the benefit of vaccination. In other words, vaccination shifted outwards the health-production possibilities frontier (i.e. for a given level of economic output, vaccination reduced the associated health loss), rather than determining a single point on that frontier. By focusing only on the direct health benefits of vaccination (and reduction in costs directly associated with COVID-19 disease reduction), our analysis effectively evaluates one particular point on the new frontier. Since this point is unlikely the welfare-maximizing point, we have underestimated the benefits of vaccination as the reviewer suggested. However, we were compelled to focus on the direct benefits of vaccination because the true welfare-maximizing point is analytically challenging to find, highly dependent on normative assumptions about social preferences, and unlikely to be the trade-off that most countries chose historically. This does not of course contradict anything that the reviewer said about the limitations of our analysis; it is simply some added nuance about this dimension of benefits of vaccination.**

**To reflect this decision, we have added the following to our methods and limitations…**

**Methods (page 9, lines 107-111): Given that non-pharmaceutical interventions, behavioral changes, and variant emergence were assumed to remain as historically observed, the benefits of COVID-19 vaccination considered in this study are limited to those related to the direct reduction in COVID-19 morbidity and mortality, and do not capture costs and benefits arising from associated policy or behavioral changes enabled by vaccination (such as relaxation of lockdowns).**

**Limitations (page 24-25, lines 477-487): This study’s ROI estimates are likely conservative as health and economic benefits from the vaccines continued to accrue beyond the first year of this administration. We assumed that non-pharmaceutical interventions with adverse macroeconomic effects, such as lockdowns, remained unchanged after vaccination. In practice, however, most countries relaxed these restrictions as vaccination reduced infection risk. Such relaxations would have led to both benefits (reduced economic losses) and costs (increased infection risks). However, vaccination shifted the health-production possibilities frontier outward: for any given level of economic output, it reduced the associated health losses. By assuming that non-pharmaceutical interventions did not change, we effectively selected the point on this frontier that preserves the pre-vaccination level of economic output. This is unlikely to be optimal and therefore likely leads us to underestimate the benefits of vaccination. Despite these cautious assumptions, COVID-19 vaccines were and remain a sound global investment.**

F. *I don’t think the term “welfarist economics” exist*

**Response: We agree – we simply mean “welfarism” but in fact have changed the language of the text so as not to ascribe to welfarism all the assumptions involved in unweighted CBA.**

G. *I don’t see a definition of the acronyms BCAs and CBAs*

**Response: Thank you for this catching this error. We have defined the CBA acronym as cost-benefit analysis. BCAs was a spelling mistake, which we have now fixed.**

H. *The study’ perspective is defined as “global payer perspective”. Maybe societal perspective at the global level would be a better characterization. It’s just that when people see “payer perspective” they automatically think of a narrow conception of costs and benefits – which does not correspond to what the authors do here.*

**Response: Thank you for this suggestion to better characterize the study perspective. We implemented this comment. Our methods now read (page 9, line 118): “The study follows a societal perspective at a global level.”**

I. *Line 132 says that QALYs were “monetised by applying societal willingness-to-pay (WTP) thresholds”. I think calling these values WTP thresholds mischaracterizes what the extra-welfarist estimates actually represent (opportunity cost thresholds). What society is willing to pay for health does not necessarily coincide with the marginal productivity of health spending (what a health system gives up when it funds one intervention rather than another).*

**Response: Since our thresholds were obtained from Pichon-Riviere et al. (2023) based on arguments around health opportunity costs, we agree with your critique and have changed the language used from “societal WTP thresholds” to “opportunity cost thresholds”.**

J. *I don’t understand why the authors are introducing a new concept (VLY) as opposed to VSLY*

**Response: We introduced this comment to more clearly describe our methods. However, we have since changed it back to be consistent with Robinson et al. (2019) and typical terminology.**

**Page 13, line 208-213: Country-specific VSLY values were obtained by dividing the VSL by the undiscounted future life expectancy of each country and then multiplying this value by the number of undiscounted life years gained from COVID-19 vaccination. In addition to the country-specific VSLYs, we also estimated an ROI using a uniform global VSLY. This VSLY was derived from a population-weighted mean VSLY from the country-specific VSLYs included in the analysis.**

**Page 19, line 338-339: To account for differences in the marginal value of money across income groups, we also applied a uniform global VSLY.**

K. *I am afraid I still don’t understand Table 1 in the SI (is each row a different value for that category?)*

**Response: Yes, each row is a different value for that category, with the associated reference in the same row. Due to limited publicly available specifications on COVID-19 vaccine funding, individual values could not always be precisely characterized and have therefore been consolidated into broader categories. Source references are provided for each value to maximize transparency and traceability. We have now added the following sentence to the Supplemental Table 1s title: “Each row corresponds to one data point obtained; however, we had to group these data points into broader categories due to limited publicly available information on COVID-19 vaccine funding.”**
