# Supplementary material for: Global return-on-investment of COVID-19 vaccines in the first year of the vaccination programme: A model-based economic evaluation
Source: PLoS Med. 2026 Nov 5;23(11):e1004772. doi: 10.1371/journal.pmed.1004772 (PMC13739145; doi:10.1371/journal.pmed.1004772)
Supplement: Supplementary file 7 [file pmed.1004772.s009.docx]

***General Editorial Requests:***

***A:*** Title: Please add the study design ("A randomized controlled trial," "A retrospective study," "A modelling study," etc.) in the subtitle after a colon.

**We added the study design to our title. Our title now reads: “A global return-on-investment of COVID-19 vaccines in the first year of the vaccination programme: A model-based economic evaluation”**

***B.*** Abstract: Please revise to include only three sections: Background, Methods and Findings, and Conclusions. The last sentence of the Methods and Findings section should describe the main limitation(s) of the study's methodology.

**We updated our abstract to reflect these requirements. Please see below.**

**Background: COVID-19 vaccines played a critical role in reducing global health burden during the COVID-19 pandemic, but their rapid development required extraordinary effort. This study aims to evaluate their global return-on-investment (ROI) during the first year of the vaccination programme (8 December 2020 to 8 December 2021) to provide insights into the economic benefits relative to the costs of vaccine development, manufacturing, and delivery.**

**Methods and Findings: Public health outcomes of COVID-19 vaccination, including life years and quality-adjusted life years (QALYs) gained from averted deaths, infections, and hospitalizations, were estimated. Health benefits were valued using two approaches: i) cost-benefit analysis and ii) cost-effectiveness analysis. These benefits were compared to the costs of vaccine development, manufacturing, and delivery to estimate the ROI under both approaches. Probabilistic sensitivity analyses were conducted to assess the impact of parameter uncertainty on ROI estimates. Vaccine development and delivery cost $79.4 billion, but the health and economic benefits of vaccination were valued between $6.43 – $64.3 trillion. High-income countries saw the greatest health benefits per-person vaccinated, and low-income countries gained the greatest non-health benefits per-person vaccinated, when expressed as a proportion of gross domestic product per capita. This estimated ROIs of $59.6 – $809 per dollar invested in the base case analysis. ROIs ranged between $60.2 – $753 across probabilistic sensitivity analyses for both approaches. Limitations include reliance on previous analyses of COVID-19 vaccination impact and a time horizon limited to the first year of COVID-19 vaccination.**

**Conclusions: Rapid COVID-19 vaccine development and delivery were an excellent investment. Vaccine development and equitable vaccine distribution globally should be prioritized before and during global health crises.**

***C.*** Please confirm that your abstract complies with our requirements, including format (three sections: Background, Methods and Findings, and Conclusions) and providing all the information relevant to this study type <https://journals.plos.org/plosmedicine/s/submission-guidelines#loc-abstract>

**We updated our Author Summary to align with these instructions.**

**Why Was this Study Done?**

- **Extraordinary rapid and large investments were made globally into developing COVID-19 vaccines during the pandemic in 2020.**
- **COVID-19 vaccination significantly reduced morbidity and mortality worldwide in its first year (December 2020 – December 2021), but it remains unclear whether these benefits were worth the investments.**

**What Did the Researchers Do and Find?**

- **Using previously published data from an epidemiological transmission model, we quantified the global health, productivity, and healthcare benefits of COVID-19 vaccination in monetary terms.**
- **We compared these benefits with the global costs of vaccine research, development, manufacturing and delivery, and assessed differences across World Bank income groups.**
- **The benefits of global COVID-19 vaccination in its first year were at least 70 times greater than the costs, although substantial disparities existed across income groups.**

**What Do These Findings Mean?**

- **Our findings mean that the investments made into COVID-19 vaccines largely paid off, promoting the need for future investments into vaccines for both health and economic purposes.**
- **The disparities observed between income groups reflect the need for equitable vaccine distribution and the sharing of vaccines and vaccine-related information across borders.**
- **Despite uncertainty in the underlying data, the large net positive return suggests that investments in COVID-19 vaccines and vaccination programs were highly worthwhile.**

***D.*** Data Availability Statement: Please include a citable digital object identifier (DOI) or other persistent identifier of the code (such as through linkage with Zenodo).

**We have been in correspondence with Senior Editor, Dr. Andreia Cunha, and will provide the DOI when it is available. We are cleaning the code to enhance reproducibility and clarity.**

***E.*** Acknowledgments: Please consider including recognition of individuals who played a role in data collection.

**Thank you for this suggestion. No other individuals were involved in the study design, manuscript writing, or data collection.**
