# Supplementary material for: Global return-on-investment of COVID-19 vaccines in the first year of the vaccination programme: A model-based economic evaluation
Source: PLoS Med. 2026 Nov 5;23(11):e1004772. doi: 10.1371/journal.pmed.1004772 (PMC13739145; doi:10.1371/journal.pmed.1004772)
Supplement: Peer review history

# Editor report: Decision Letter 0

**DOI:** 10.1371/journal.pmed.1004772.r001

**Authors:** Andreia Cunha

17 Sep 2025

Dear Dr Benjamin,

Thank you for submitting your manuscript entitled "The global return-on-investment of COVID-19 vaccines in the first year of the vaccination programme" for consideration by PLOS Medicine.

Your manuscript has now been evaluated by the PLOS Medicine editorial staff as well as by an academic editor with relevant expertise and I am writing to let you know that we would like to send your submission out for external peer review.

However, before we can send your manuscript to reviewers, we need you to complete your submission by providing the metadata that is required for full assessment. To this end, please login to Editorial Manager where you will find the paper in the 'Submissions Needing Revisions' folder on your homepage. Please click 'Revise Submission' from the Action Links and complete all additional questions in the submission questionnaire.

For clinical studies, please upload a copy of your trial study protocol as a supporting information file. The study protocol should be the version submitted for approval to the institutional review board or ethics committee, should include any amendments to the study protocol, as well as the date of their approval by the institutional review or ethics committee. Please also detail any deviations from the study protocol in the Methods section of your manuscript. The editors will consider the protocol and study conduct prior to a final decision for external review.

Please re-submit your manuscript within two working days, i.e. by Sep 19 2025 11:59PM.

Login to Editorial Manager here: https://www.editorialmanager.com/pmedicine

Once your full submission is complete, your paper will undergo a series of checks in preparation for peer review. Once your manuscript has passed all checks it will be sent out for review.

Feel free to email us at plosmedicine@plos.org if you have any queries relating to your submission.

Kind regards,

Andreia Cunha, PhD

Senior Editor

PLOS Medicine

# Peer review: Decision Letter 1

**DOI:** 10.1371/journal.pmed.1004772.r002

**Authors:** Andreia Cunha

13 Nov 2025

Dear Dr Benjamin,

Sincere apologies for the delay in getting back to you with a decision and many thanks for submitting your manuscript "The global return-on-investment of COVID-19 vaccines in the first year of the vaccination programme" (PMEDICINE-D-25-03197R1) to PLOS Medicine. The paper has been reviewed by subject experts and a statistician; their comments are included below and can also be accessed here: [LINK]

As you will see, the reviewers find the work of great interest, but they raise very important points about the analyses. After discussing the paper with the editorial team and an academic editor with relevant expertise, I'm pleased to invite you to revise the paper in response to the reviewers' comments. We plan to send the revised paper to some or all of the original reviewers, and we cannot provide any guarantees at this stage regarding publication.

In addition to these revisions, you may need to complete some formatting changes, which you will receive in a follow up email. A member of our team will be in touch with a set of requests shortly. If you do not receive a separate email within a few days, please assume that checks have been completed, and no additional changes are required.

When you upload your revision, please include a point-by-point response that addresses all of the reviewer and editorial points, indicating the changes made in the manuscript and either an excerpt of the revised text or the location (eg: page and line number) where each change can be found. Please also be sure to check the general editorial comments at the end of this letter and include these in your point-by-point response. When you resubmit your paper, please include a clean version of the paper as the main article file and a version with changes tracked as a marked-up manuscript. It may also be helpful to check the guidelines for revised papers at http://journals.plos.org/plosmedicine/s/revising-your-manuscript for any that apply to your paper.

We ask that you submit your revision by Feb 04 2026 11:59PM. However, if this deadline is not feasible, please contact me by email, and we can discuss a suitable alternative.

Don't hesitate to contact me directly with any questions (acunha@plos.org).

Best regards,

Andreia

Andreia Cunha, PhD

Senior editor

PLOS Medicine

acunha@plos.org

-----------------------------------------------------------

Comments from the reviewers:

Reviewer #1: The paper "The global return-on-investment of COVID-19 vaccines in the first year of the vaccination programme" by Benjamin et al., addresses a key gap in the literature by assessing the economic value of COVID-19 vaccines in first year after their global roll out. It shows that vaccines played a major role in reducing the global health burden during the pandemic, and estimates that for every dollar spent on development and delivery in the first year, the return ranged from $59 to $475. These findings highlight the extraordinary value of investing in vaccines, both for public health and the global economy but also showcase how unequal benefits are distributed globally.

It is a necessary study that will be of interest to the readers of the journal. I did not completely follow a few decisions the authors made and appreciate that my points below might be addressed by improving the clarity and provided rationales. However, alternatively major revisions may be required especially re the mortality risk reduction valuation.

Background

The introduction provides a concise and engaging overview of the existing literature, the research gap, and the study's objectives. However, the language could be clearer in places. For instance, the authors refer to "development, manufacturing, and distribution costs," but it's not immediately clear what these encompass. In the methods section, "delivery costs" are mentioned, yet it remains ambiguous whether this includes costs beyond the point of vaccination—such as storage at administration sites or the costs of administering the vaccine. Clarifying this distinction would strengthen the transparency of the cost analysis and this needs to be taken care off across the whole work. Finally, the last sentence of the introduction could be simplified to more clearly convey its meaning and relevance to the results section.

Methods

The methods section is concise, maybe to conscise, but useful detail is provided in the appendix. However, the authors should more clearly explain how costs and benefits were aggregated and monetised. Referring to the *Reference Case Guidelines for Benefit-Cost Analysis in Global Health* (Robinson et al., 2019) could help clarify this, even if the approach does not explicitly follows this guidance. It should be explicitly stated whether immunisation-related costs—such as administration, side effects, and productivity losses from attending vaccination—are included or excluded and the implications of the decision could be discussed in the relevant section. A table or graphic summarising inputs, outcomes, and monetisation approaches would greatly improve clarity and transparency.

Moreover, the rationale for valuing mortality risk reduction using life years gained multiplied by VSLY, rather than number of deaths multiplied by VSL (or both), should be explained in the main text. Since VSLY introduces an implicit age adjustment of mortality risk reduction, recent guidance (e.g. Robinson et al., OECD 2023) recommends presenting this as a sensitivity analysis. This would also improve comparability with studies such as Owsawa et al., even if it results in higher ROI estimates. This choice is therefore unclear to me and there should be at least a strong rational for why VSL valuation was not performed.

It is also not clear to me why the welfarist approach would exclude all the value from non-fatal events? They might be small compared to mortality risk reduction, I agree, but the authors should lay out their rational why productivity costs averted for those who survive and HCRU is not inlcuded.

Results

The results are adequately described and clearly presented. However, the authors may wish to consider whether a graphical representation of parts of the results section would be worthwhile.

Discussion

The discussion is engaging and well-structured, but the authors should place greater emphasis on the implications of how mortality benefits are valued. Specifically, more attention should be given to what VSL and VSLY represent in terms of tangible and intangible benefits, and how these choices influence the overall ROI estimates. For example, in the extra-welfarist approach results section, the productivity gains alone appear to exceed the total development and delivery costs by a significant margin, which should be highlighted as it is of interest to decision makers sitting in the relevant departments for economic planning. Clarifying these valuation choices and their impact would strengthen the interpretation of the results and help readers better understand the drivers of the estimated returns.

Reviewer #2: Overall Summary

This manuscript offers a timely and rigorous health economic analysis of the global return on investment (ROI) from COVID-19 vaccination. The study's strengths are evident in its robust methodology, extensive sensitivity analyses, and clear policy relevance. The authors employ both extra-welfarist and welfarist perspectives to monetize health benefits, demonstrating strong technical rigor. The results - showing ROIs far exceeding 1 under all assumptions - convincingly underscore that COVID-19 vaccination is a highly cost-beneficial public health investment. The manuscript is well-written and the discussion appropriately acknowledges key drivers (e.g. differential benefits by income level) and limitations. In summary, this is an important and well-executed study that provides valuable evidence to policymakers about the enormous economic and health returns of vaccination. Only a few minor clarifications and additions are recommended below to further strengthen the context and clarity of the work.

Major Comments

*Contrast with Prior ROI Estimates: To bolster the discussion, the authors could consider explicitly contrasting their ROI findings with those of previous studies. For instance, various papers including (but not limited to!) Theakston et al., (2025) and Harrison et al., (2025) report an ROI of roughly 19:1 for adult immunization programs when broader societal benefits are monetized. Further, El Banhawi et al., (2025) emphasize that analyses centered on the endemic period also found that the benefit of COVID-19 vaccination is large including to the national economy by averting productivity losses (in addition to healthcare costs). The authors are encouraged to note that differences in ROI estimates across studies likely stem from the focus on short-term, peak pandemic effects versus longer-term endemic vaccination strategies. By situating their results in this context, the authors can clarify that their own ROI estimates, while high, are nevertheless relatively conservative (covering the first-year program with comprehensive but bounded assumptions) and complementary to prior published estimates. This addition will reinforce the message that, whether one examines the intense initial pandemic phase or more routine immunization periods, COVID-19 vaccination consistently delivers a high return on investment under various scenario. This would strengthen the paper's positioning in the literature and assure readers that the findings align with - and add to - the growing evidence base on vaccination's value.

Minor Comments / Suggestions for Improvement

*Terminology Clarification: For broader readership clarity, consider adding a concise explanation of the "welfarist" vs "extra-welfarist" approaches when first mentioned. Not all readers (e.g. policymakers or general public health audience) will be familiar with these health economics terms. A brief description of how the welfarist approach monetizes life-years (e.g. via value of statistical life or VSLY) versus the extra-welfarist approach (focused on direct costs, QALYs, and cost-of-illness savings) would help readers intuitively understand the two ROI figures presented for each scenario. This will ensure the impressive ROI results are interpretable to non-economist stakeholders.

*Contextualize ROI as Conservative: The discussion could more plainly state that the ROI estimated here is likely conservative (particularly under the extra-welfarist perspective) yet emphasize that despite this it is still extraordinarily high, reinforcing that even under cautious assumptions COVID-19 was and remains a sound investment. Another suggestion is to also more plainly state the the ROI using VSL estmates does not mean that every dollar invested will deliver the estimated return in cash. The authors are very prudent already by consistently using the term economic returns rather than financial but it may be good to leave no doubt as some readers may otherwise not fully appreciatethis important nuance.

*Minor Edits and Clarity: The manuscript is generally clear and well-structured. I encourage the authors to double-check that all acronyms (e.g., ROI, QALY, VSLY, LMIC, etc.) are defined at first use for clarity. In addition, ensure that figures and tables are referenced in order and that their captions are self-explanatory. For example, Table 4 presents ROI results; a brief mention in the text guiding the reader to "Table 4 shows that all ROI estimates exceed 1 (i.e., net positive returns)" would be helpful. Just some small editorial suggestions to enhance readability.

Overall, the authors are to be commended for a high-quality analysis. With the above minor additions, the paper will convincingly communicate that investment in COVID-19 vaccination yields substantial health and economic dividends, complementing earlier estimates and providing a strong evidence-based argument for continued support of vaccination programs globally. The revisions will further solidify the manuscript's contribution and clarity for both the journal's academic readership and decision-makers who may act on its findings. Congratulations on an excellent study.

________________________________________

Theakston C, Napier M, Brassel S, et al. A benefit-cost analysis quantifying the broader socioeconomic value of adult respiratory vaccination programs in Japan. Expert Rev Vaccines. 2025;24(1):633-643.

Harrison C, Brassel S, Chowdhury S, et al. The broader socio-economic value of adult respiratory disease vaccinations in the UK - results from a benefit cost analysis. Expert Rev Vaccines. 2025;24(1)

El Banhawi H., Hodgson S., Neri M., Steuten L. The broader economic benefits of COVID-19 vaccination (2025). Contract Research. Available from https://www.ohe.org/publications/the-broader-economic-benefits-of-covid-19-vaccination/.

Reviewer #3: Thank you to the authors for submitting this paper, it is clearly on an important topic.

This is clearly a well written paper, but to be honest I'm struggling to see why this needs to be published given the previous evidence base in this area. Some specific comments below.

Background

- I think the authors need to further justify the need for the paper. There have seemingly been a variety of economic evaluations at a country level. What is the specific need for a combined study also including the vaccine development?

Methods

- Need a lot more about the Watson et al model in the main text. This is a key part of the paper and there's one paragraph on it

- I would strongly suggest that the authors drop the undiscounted analysis, there is simply no need to present these results if the time horizon of the analyses is beyond one year.

- Need more information from Appendix B in the main text related to the benefits. Nobody really reads the appendices.

Results

- The numbers quoted in the results are so large and heterogenous that they don't really mean anything. I think more work needs to be done to put these into more context (if possible)

Discussion

- For me, the headline results (estimated returns of $42 - $775 per dollar) are so heterogenous that they're pretty meaningless. This is no surprise given the limitations regarding the data mentioned later in the discussion. Perhaps these need to be presented differently (i.e. under what conditions would the return be $42, under what conditions would the return be $775 etc)

- Alternatively, could the presentation of these results be reframed as upper/lower bounds of the ROI?

- I would personally emphasise the disparity amongst income groups more (as for me this is the most interesting part of the study)

Reviewer #5: Report on PMEDICINE-D-25-03197R1 "The global return-on-investment of COVID-19 vaccines in the first year for the vaccination programme."

This paper makes an important contribution to the literature. It calculates, in a careful way, the return on investment for the development and delivery of the COVID vaccine. The estimates are, not surprisingly, very large with ROIs between $60 and $475 dollars per dollar invested in vaccine development and delivery.

Below I have some comments that I believe can make the paper stronger.

In the third paragraph the authors motivate the paper by citing surveys on vaccine skepticism. I'm not convinced this is a good way to motivate the paper. Simply put, this paper is not going to convince any vaccine skeptics, and they are not really the audience of the paper. I very much prefer the motivation to shape future policy about pandemic preparation. It may also be helpful to point out that this paper informs the debate about the pricing of the COVID vaccines. During the pandemic, there was discontent that companies that took public money could sell back the vaccine to the public at high prices. This paper shows that the surplus is so enormous that even at high prices there is substantial benefit to the public.

On page 4 I think the paper needs a clearer discussion of the difference between the welfarist and extra-welfarist approach. Specifically, the paper says the extra-welfarist approach broadens the evaluation. The reader may then be surprised that the broader extra-welfarist approach produces smaller benefits than the welfarist approach. This is a common result because VSL measures value a QALY more highly than extra-welfarist approach. The paper should clarify this issue.

On pp. 6 I am confused by a few things in paragraph 4. For example, "it was estimated that COVID-19 vaccination generated and economic value of 27.2 trillion." I think this is a description of table 2. But table 2 lists the benefits as 35 trillion. I am having similar difficulty mapping the number in this paragraph for the extra-welfarist approach as well in table 3. Also, should the benefits across the four income groups add up to the total benefit? Finally, I don't understand how applying a discount rate increased the measured benefits from 27.2 trillion to 37.8 trillion (paragraph 4).

In paragraph 5 on pp. 6, it would be more accurate to say the value of QALYs gained decreased instead of just QALYs gained decreased since the unit of measurement is dollars here.

The last paragraph on page six states that the ROI is well in excess of 1. This is such an understatement that I would consider revising it.

In the last paragraph on page 7 it is important to note that the benefits are high for high income countries because their VSL estimates are higher as well.

In the discussion on page 8 of on equitable vaccine distribution. I would consider the Baker et al. (2021) point that investing in vaccine capacity to increase the supply of vaccines could help allowing for a more equitable distribution by limiting the incentive for vaccine nationalism.

In summary, this is a nice paper which could be improved with a minor revision. In my view, the most urgent issue is to clarify the discrepancy between the text and table 2 and 3. And I hope the other suggestions are useful as well.

References

Baker, Arthur, Esha Chaudhuri, and Michael Kremer. "Accelerating vaccinations." Finance Dev (2021): 12-16.

Any attachments provided with reviews can be seen via the following link: [LINK]

--------------------------------------------------------- ---

General editorial requests:

(Note: not all will apply to your paper, but please check each item carefully)

*We ask every co-author listed on the manuscript to fill in a contributing author statement, making sure to declare all competing interests. If any of the co-authors have not filled in the statement, we will remind them to do so when the paper is revised. If all statements are not completed in a timely fashion this could hold up the re-review process. If new competing interests are declared later in the revision process, this may also hold up the submission. Should there be a problem getting one of your co-authors to fill in a statement we will be in contact. Please do not add or remove authors without first discussing this with the handling editor. You can see our competing interests policy here: http://journals.plos.org/plosmedicine/s/competing-interests.

*Please upload any figures associated with your paper as individual TIF or EPS files with 300dpi resolution at resubmission; please read our figure guidelines for more information on our requirements: http://journals.plos.org/plosmedicine/s/figures. While revising your submission, we strongly recommend that you use PLOS's NAAS tool (https://ngplosjournals.pagemajik.ai/artanalysis) to test your figure files. NAAS can convert your figure files to the TIFF file type and meet basic requirements (such as print size, resolution), or provide you with a report on issues that do not meet our requirements and that NAAS cannot fix.

After uploading your figures to PLOS's NAAS tool - https://ngplosjournals.pagemajik.ai/artanalysis, NAAS will process the files provided and display the results in the "Uploaded Files" section of the page as the processing is complete.

If the uploaded figures meet our requirements (or NAAS is able to fix the files to meet our requirements), the figure will be marked as "fixed" above. If NAAS is unable to fix the files, a red "failed" label will appear above.

When NAAS has confirmed that the figure files meet our requirements, please download the file via the download option, and include these NAAS processed figure files when submitting your revised manuscript.

*Please ensure that the paper adheres to the PLOS Data Availability Policy (see http://journals.plos.org/plosmedicine/s/data-availability), which requires that all data underlying the study's findings be provided in a repository or as Supporting Information. For data residing with a third party, authors are required to provide instructions with contact information (web or email address) for obtaining the data. Please note that a study author cannot be the contact person for the data. PLOS journals do not allow statements supported by "data not shown" or "unpublished results." For such statements, authors must provide supporting data or cite public sources that include it.

*We expect all researchers with submissions to PLOS in which author-generated code underpins the findings in the manuscript to make all author-generated code available without restrictions upon publication of the work. In cases where code is central to the manuscript, we may require the code to be made available as a condition of publication. Authors are responsible for ensuring that the code is reusable and well documented. Please make any custom code available, either as part of your data deposition or as a supplementary file. Please add a sentence to your data availability statement regarding any code used in the study, e.g. "The code used in the analysis is available from Github [URL] and archived in Zenodo [DOI link]" Please review our guidelines at https://journals.plos.org/plosmedicine/s/materials-software-and-code-sharing and ensure that your code is shared in a way that follows best practice and facilitates reproducibility and reuse. Because Github depositions can be readily changed or deleted, we encourage you to make a permanent DOI'd copy (e.g. in Zenodo) and provide the URL.

FORMATTING - GENERAL

*Abstract: Please structure your abstract using the PLOS Medicine headings (Background, Methods and Findings, Conclusions). Please combine the Methods and Findings sections into one section.

*At this stage, we ask that you include a short, non-technical Author Summary of your research to make findings accessible to a wide audience that includes both scientists and non-scientists. The Author Summary should immediately follow the Abstract in your revised manuscript. This text is subject to editorial change and should be distinct from the scientific abstract. Ideally each sub-heading should contain 2-3 single sentence, concise bullet points containing the most salient points from your study. In the final bullet point of 'What Do These Findings Mean?', please include the main limitations of the study in non-technical language. Please see our author guidelines for more information: https://journals.plos.org/plosmedicine/s/revising-your-manuscript#loc-author-summary.

*Please express the main results with 95% CIs as well as p values. When reporting p values please report as p<0.001 and where higher as the exact p value p=0.002, for example. Throughout, suggest reporting statistical information as follows to improve clarity for the reader "22% (95% CI [13%,28%]; p</=)". Please be sure to define all numerical values at first use.

*Please include page numbers and line numbers in the manuscript file. Use continuous line numbers (do not restart the numbering on each page).

*Please cite the reference numbers in square brackets. Citations should precede punctuation.

FIGURES AND TABLES

*Please provide titles and legends for all figures and tables (including those in Supporting Information files).

*Please define all abbreviations used in each figure/table (including those in Supporting Information files).

*Please consider avoiding the use of red and green in order to make your figure more accessible to those with color blindness.

SUPPLEMENTARY MATERIAL

*Please note that supplementary material will be posted as supplied by the authors. Therefore, please amend it according to the relevant comments outlined here.

*Please cite your Supporting Information as outlined here: https://journals.plos.org/plosmedicine/s/supporting-information

REFERENCES

*PLOS uses the numbered citation (citation-sequence) method and first six authors, et al.

*Please ensure that journal name abbreviations match those found in the National Center for Biotechnology Information (NCBI) databases (http://www.ncbi.nlm.nih.gov/nlmcatalog/journals), and are appropriately formatted and capitalised.

*Where website addresses are cited, please include the complete URL and specify the date of access (e.g. [accessed: 12/06/2023]).

*Please also see https://journals.plos.org/plosmedicine/s/submission-guidelines#loc-references for further details on reference formatting.

MODELLING STUDIES

The following list is derived from Geoffrey P Garnett, Simon Cousens, Timothy B Hallett, Richard Steketee, Neff Walker. Mathematical models in the evaluation of health programmes. (2011) Lancet DOI:10.1016/S0140-6736(10)61505-X:

*If pertinent, please provide a diagram that shows the model structure, including how the natural history of the disease is represented, the process and determinants of disease acquisition, and how the putative intervention could affect the system.

*Please provide a complete list of model parameters, including clear and precise descriptions of the meaning of each parameter, together with the values or ranges for each, with justification or the primary source cited and important caveats about the use of these values noted.

*Please provide a clear statement about how the model was fitted to the data, including goodness-of-fit measure, the numerical algorithm used, which parameter varied, constraints imposed on parameter values, and starting conditions.

*For uncertainty analyses, please state the sources of uncertainties quantified and not quantified [can include parameter, data, and model structure].

*Please provide sensitivity analyses to identify which parameter values are most important in the model. Uncertainty estimates seek to derive a range of credible results on the basis of an exploration of the range of reasonable parameter values. The choice of method should be presented and justified.

*Please discuss the scientific rationale for the choice of model structure and identify points where this choice could influence conclusions drawn. Please also describe the strength of the scientific basis underlying the key model assumptions.

HEALTH ECONOMICS / COST-EFFECTIVENESS STUDIES

*Please ensure that the study is reported according to the CHEERS guideline (available from: https://www.equator-network.org/reporting-guidelines/cheers) and include the completed checklist as Supporting Information. Please add the following statement, or similar, to the Methods: "This study is reported as per the Strengthening the Consolidated Health Economic Evaluation Reporting Standards 2022 (CHEERS 2022) Statement (S1 Checklist)." When completing the checklist, please use section and paragraph numbers, rather than page numbers.

Attachment Submitted filename: Review PMEDICINE-D-25.docx

# Author comment: Author response to Decision Letter 2

**DOI:** 10.1371/journal.pmed.1004772.r003

6 Mar 2026

Attachment Submitted filename: FINAL_Response to Reviewers.docx

# Peer review: Decision Letter 2

**DOI:** 10.1371/journal.pmed.1004772.r004

**Authors:** Andreia Cunha

11 May 2026

Dear Dr Benjamin,

Sincere apologies for the delay in getting back to you with a decision, which was due to challenges in securing all the necessary advice. Many thanks for submitting your manuscript "The global return-on-investment of COVID-19 vaccines in the first year of the vaccination programme" (PMEDICINE-D-25-03197R2) to PLOS Medicine. The paper has been reviewed by four of the original reviewers and a statistician; their comments are included below and can also be accessed here: [LINK]

As you will see, most of the reviewers are satisfied with the revisions. However, Reviewer 1 has some suggestions for minor text revisions and Reviewer 4 still has some concerns we must ask you to address. After discussing the paper with an academic editor with relevant expertise, I'm pleased to invite you to revise the paper in response to the reviewers' comments. We plan to send the revised paper to some or all of the original reviewers, and we cannot provide any guarantees at this stage regarding publication.

In addition to these revisions, you may need to complete some formatting changes, which you will receive in a follow up email. A member of our team will be in touch with a set of requests shortly. If you do not receive a separate email within a few days, please assume that checks have been completed, and no additional changes are required.

When you upload your revision, please include a point-by-point response that addresses all of the reviewer and editorial points, indicating the changes made in the manuscript and either an excerpt of the revised text or the location (eg: page and line number) where each change can be found. Please also be sure to check the general editorial comments at the end of this letter and include these in your point-by-point response. When you resubmit your paper, please include a clean version of the paper as the main article file and a version with changes tracked as a marked-up manuscript. It may also be helpful to check the guidelines for revised papers at http://journals.plos.org/plosmedicine/s/revising-your-manuscript for any that apply to your paper.

We ask that you submit your revision by Jun 15 2026 11:59PM. However, if this deadline is not feasible, please contact me by email, and we can discuss a suitable alternative.

Don't hesitate to contact me directly with any questions (acunha@plos.org).

Best regards,

Andreia

Andreia Cunha, PhD

Senior editor

PLOS Medicine

acunha@plos.org

-----------------------------------------------------------

Comments from the reviewers:

Reviewer #1: I congratulate the author's to the great revision of their paper. This was an excellent rebuttal letter to read and I thank the authors for answering each reviewer in such detail.

I only have two minor comments and I believe, if fixed, this work will be of great interest to the readers of PLOS Medicine.

Minor comments.

L 73: "Furthermore, the welfarist approach does not admit broader societal values. " I do think that could be better phrased, because compared to extra-welfarism, welfarism is the broader framework as it takes individual preferences across all domains of life into account. I think it is more that the welfarist approach does not accommodate values that cannot be expressed through individual utility. Hence, I'd consider rephrasing the sentence.

I am also aware that reference 32 (Banhawi et. al 2025) was now published in two journals. The quantitative results the author's are referring two is published in:https://pubmed.ncbi.nlm.nih.gov/41410417/

and the targeted review was published in: https://pubmed.ncbi.nlm.nih.gov/39203978/.

Very best wishes.

Reviewer #3: The authors have comprehensively responded to my comments, I am happy to accept.

Any attachments provided with reviews can be seen via the following link: [LINK]

------------------------------------------------------------

General editorial requests:

*We ask every co-author listed on the manuscript to fill in a contributing author statement, making sure to declare all competing interests. If any of the co-authors have not filled in the statement, we will remind them to do so when the paper is revised. If all statements are not completed in a timely fashion this could hold up the re-review process. If new competing interests are declared later in the revision process, this may also hold up the submission. Should there be a problem getting one of your co-authors to fill in a statement we will be in contact. Please do not add or remove authors without first discussing this with the handling editor. You can see our competing interests policy here: http://journals.plos.org/plosmedicine/s/competing-interests.

*Please upload any figures associated with your paper as individual TIF or EPS files with 300dpi resolution at resubmission; please read our figure guidelines for more information on our requirements: http://journals.plos.org/plosmedicine/s/figures. While revising your submission, we strongly recommend that you use PLOS's NAAS tool (https://ngplosjournals.pagemajik.ai/artanalysis) to test your figure files. NAAS can convert your figure files to the TIFF file type and meet basic requirements (such as print size, resolution), or provide you with a report on issues that do not meet our requirements and that NAAS cannot fix.

After uploading your figures to PLOS's NAAS tool - https://ngplosjournals.pagemajik.ai/artanalysis, NAAS will process the files provided and display the results in the "Uploaded Files" section of the page as the processing is complete.

If the uploaded figures meet our requirements (or NAAS is able to fix the files to meet our requirements), the figure will be marked as "fixed" above. If NAAS is unable to fix the files, a red "failed" label will appear above.

When NAAS has confirmed that the figure files meet our requirements, please download the file via the download option, and include these NAAS processed figure files when submitting your revised manuscript.

*Please ensure that the paper adheres to the PLOS Data Availability Policy (see http://journals.plos.org/plosmedicine/s/data-availability), which requires that all data underlying the study's findings be provided in a repository or as Supporting Information. For data residing with a third party, authors are required to provide instructions with contact information (web or email address) for obtaining the data. Please note that a study author cannot be the contact person for the data. PLOS journals do not allow statements supported by "data not shown" or "unpublished results." For such statements, authors must provide supporting data or cite public sources that include it.

*We expect all researchers with submissions to PLOS in which author-generated code underpins the findings in the manuscript to make all author-generated code available without restrictions upon publication of the work. In cases where code is central to the manuscript, we require the code to be made available as a condition of publication. Authors are responsible for ensuring that the code is reusable and well documented. Please make any custom code available, either as part of your data deposition or as a supplementary file. Please add a sentence to your data availability statement regarding any code used in the study, e.g. "The code used in the analysis is available from Github [URL] and archived in Zenodo [DOI link]" Please review our guidelines at https://journals.plos.org/plosmedicine/s/materials-software-and-code-sharing and ensure that your code is shared in a way that follows best practice and facilitates reproducibility and reuse. Because Github depositions can be readily changed or deleted, we encourage you to make a permanent DOI'd copy (e.g. in Zenodo) and provide the URL.

*The funding statement should include: specific grant numbers, initials of authors who received each award, URLs to sponsors’ websites. Also, please state whether any sponsors or funders (other than the named authors) played any role in study design, data collection and analysis, the decision to publish, or preparation of the manuscript. If they had no role in the research, include this sentence: “The funders had no role in study design, data collection and analysis, decision to publish, or preparation of the manuscript.”

*All authors must declare their relevant competing interests per the PLOS policy, which can be seen here: https://journals.plos.org/plosmedicine/s/competing-interests For authors with ties to industry, please indicate whether any of the interests has a financial stake in the results of the current study.

*Please ensure that the study is reported according to the guidelines below and include the completed checklists as Supporting Information. When completing the checklist, please use section and paragraph numbers, rather than page numbers. Please add the following statement, or similar, to the Methods: "This study is reported as per ... guideline (S1 Checklist).

FORMATTING - GENERAL

*Abstract: Please structure your abstract using the PLOS Medicine headings (Background, Methods and Findings, Conclusions). Please combine the Methods and Findings sections into one section.

*At this stage, we ask that you include a short, non-technical Author Summary of your research to make findings accessible to a wide audience that includes both scientists and non-scientists. The Author Summary should immediately follow the Abstract in your revised manuscript. This text is subject to editorial change and should be distinct from the scientific abstract. Ideally each sub-heading should contain 2-3 single sentence, concise bullet points containing the most salient points from your study. In the final bullet point of 'What Do These Findings Mean?', please include the main limitations of the study in non-technical language. Please see our author guidelines for more information: https://journals.plos.org/plosmedicine/s/revising-your-manuscript#loc-author-summary.

*Please express the main results with 95% CIs as well as p values. When reporting p values please report as p<0.001 and where higher as the exact p value p=0.002, for example. Throughout, suggest reporting statistical information as follows to improve clarity for the reader "22% (95% CI [13%,28%]; p</=)". Please be sure to define all numerical values at first use.

*Please include page numbers and line numbers in the manuscript file. Use continuous line numbers (do not restart the numbering on each page).

*Please cite the reference numbers in square brackets. Citations should precede punctuation.

FIGURES AND TABLES

*Please provide titles and legends for all figures and tables (including those in Supporting Information files).

*Please define all abbreviations used in each figure/table (including those in Supporting Information files).

*Please consider avoiding the use of red and green in order to make your figure more accessible to those with color blindness.

SUPPLEMENTARY MATERIAL

*Please note that supplementary material will be posted as supplied by the authors. Therefore, please amend it according to the relevant comments outlined here.

*Please cite your Supporting Information as outlined here: https://journals.plos.org/plosmedicine/s/supporting-information

REFERENCES

*PLOS uses the numbered citation (citation-sequence) method and first six authors, et al.

*Please ensure that journal name abbreviations match those found in the National Center for Biotechnology Information (NCBI) databases (http://www.ncbi.nlm.nih.gov/nlmcatalog/journals), and are appropriately formatted and capitalised.

*Where website addresses are cited, please include the complete URL and specify the date of access (e.g. [accessed: 12/06/2023]).

*Please also see https://journals.plos.org/plosmedicine/s/submission-guidelines#loc-references for further details on reference formatting.

MODELLING STUDIES

The following list is derived from Geoffrey P Garnett, Simon Cousens, Timothy B Hallett, Richard Steketee, Neff Walker. Mathematical models in the evaluation of health programmes. (2011) Lancet DOI:10.1016/S0140-6736(10)61505-X:

*If pertinent, please provide a diagram that shows the model structure, including how the natural history of the disease is represented, the process and determinants of disease acquisition, and how the putative intervention could affect the system.

*Please provide a complete list of model parameters, including clear and precise descriptions of the meaning of each parameter, together with the values or ranges for each, with justification or the primary source cited and important caveats about the use of these values noted.

*Please provide a clear statement about how the model was fitted to the data, including goodness-of-fit measure, the numerical algorithm used, which parameter varied, constraints imposed on parameter values, and starting conditions.

*For uncertainty analyses, please state the sources of uncertainties quantified and not quantified [can include parameter, data, and model structure].

*Please provide sensitivity analyses to identify which parameter values are most important in the model. Uncertainty estimates seek to derive a range of credible results on the basis of an exploration of the range of reasonable parameter values. The choice of method should be presented and justified.

*Please discuss the scientific rationale for the choice of model structure and identify points where this choice could influence conclusions drawn. Please also describe the strength of the scientific basis underlying the key model assumptions.

*For studies that develop a prediction model or evaluate its performance, please ensure that the study is reported according to the TRIPOD statement (https://www.equator-network.org/reporting-guidelines/tripod-statement) and include the completed checklist as Supporting Information. Please add the following statement, or similar, to the Methods: "This study is reported as per the Transparent Reporting of a Multivariable Prediction Model for Individual Prognosis Or Diagnosis (TRIPOD) statement (S1 Checklist)." For studies using machine learning, please use the TRIPOD-AI checklist. When completing the checklist, please use section and paragraph numbers, rather than page numbers.

HEALTH ECONOMICS / COST-EFFECTIVENESS STUDIES

*Please ensure that the study is reported according to the CHEERS guideline (available from: https://www.equator-network.org/reporting-guidelines/cheers) and include the completed checklist as Supporting Information. Please add the following statement, or similar, to the Methods: "This study is reported as per the Strengthening the Consolidated Health Economic Evaluation Reporting Standards 2022 (CHEERS 2022) Statement (S1 Checklist)." When completing the checklist, please use section and paragraph numbers, rather than page numbers.

Attachment Submitted filename: Review PMEDICINE-D-25_R2.docx

# Author comment: Author response to Decision Letter 3

**DOI:** 10.1371/journal.pmed.1004772.r005

18 Jun 2026

Attachment Submitted filename: FINAL_Response to Reviewers2_June12.docx

# Peer review: Decision Letter 3

**DOI:** 10.1371/journal.pmed.1004772.r006

**Authors:** Andreia Cunha

11 Aug 2026

Dear Dr. Benjamin,

Thank you very much for re-submitting your manuscript "The global return-on-investment of COVID-19 vaccines in the first year of the vaccination programme" (PMEDICINE-D-25-03197R3) for review by PLOS Medicine and my sincere apologies for the delay in getting back to you with a decision.

I have discussed the paper with my colleagues, the academic editor, and it was also seen again by one of the original reviewers. I am pleased to say that provided the remaining editorial and production issues are dealt with we are planning to accept the paper for publication in the journal.

The remaining issues that need to be addressed are listed at the end of this email. Any accompanying reviewer attachments can be seen via the link below. Please take these into account before resubmitting your manuscript:

[LINK]

***Please note while forming your response, if your article is accepted, you may have the opportunity to make the peer review history publicly available. The record will include editor decision letters (with reviews) and your responses to reviewer comments. If eligible, we will contact you to opt in or out.***

In revising the manuscript for further consideration here, please ensure you address the specific points made by each reviewer and the editors. In your rebuttal letter you should indicate your response to the reviewers' and editors' comments and the changes you have made in the manuscript. Please submit a clean version of the paper as the main article file. A version with changes marked must also be uploaded as a marked up manuscript file.

Please also check the guidelines for revised papers at http://journals.plos.org/plosmedicine/s/revising-your-manuscript for any that apply to your paper. If you haven't already, we ask that you provide a short, non-technical Author Summary of your research to make findings accessible to a wide audience that includes both scientists and non-scientists. The Author Summary should immediately follow the Abstract in your revised manuscript. This text is subject to editorial change and should be distinct from the scientific abstract.

In addition to these revisions, you may need to complete some formatting changes, which you will receive in a follow up email. A member of our team will be in touch with a set of requests shortly. If you do not receive a separate email within a few days, please assume that checks have been completed, and no additional changes are required.

We expect to receive your revised manuscript within 1 week. Please email us (plosmedicine@plos.org) if you have any questions or concerns.

We ask every co-author listed on the manuscript to fill in a contributing author statement. If any of the co-authors have not filled in the statement, we will remind them to do so when the paper is revised. If all statements are not completed in a timely fashion this could hold up the re-review process. Should there be a problem getting one of your co-authors to fill in a statement we will be in contact. YOU MUST NOT ADD OR REMOVE AUTHORS UNLESS YOU HAVE ALERTED THE EDITOR HANDLING THE MANUSCRIPT TO THE CHANGE AND THEY SPECIFICALLY HAVE AGREED TO IT.

Please ensure that the paper adheres to the PLOS Data Availability Policy (see http://journals.plos.org/plosmedicine/s/data-availability), which requires that all data underlying the study's findings be provided in a repository or as Supporting Information. For data residing with a third party, authors are required to provide instructions with contact information for obtaining the data. PLOS journals do not allow statements supported by "data not shown" or "unpublished results." For such statements, authors must provide supporting data or cite public sources that include it.

To enhance the reproducibility of your results, we recommend that you deposit your laboratory protocols in protocols.io, where a protocol can be assigned its own identifier (DOI) such that it can be cited independently in the future. Additionally, PLOS ONE offers an option to publish peer-reviewed clinical study protocols. Read more information on sharing protocols at https://plos.org/protocols?utm_medium=editorial-email&utm_source=authorletters&utm_campaign=protocols

Please review your reference list to ensure that it is complete and correct. If you have cited papers that have been retracted, please include the rationale for doing so in the manuscript text, or remove these references and replace them with relevant current references. Any changes to the reference list should be mentioned in the rebuttal letter that accompanies your revised manuscript.

Please note, when your manuscript is accepted, an uncorrected proof of your manuscript will be published online ahead of the final version, unless you've already opted out via the online submission form. If, for any reason, you do not want an earlier version of your manuscript published online or are unsure if you have already indicated as such, please let the journal staff know immediately at plosmedicine@plos.org.

If you have any questions in the meantime, please contact me or the journal staff on plosmedicine@plos.org.

We look forward to receiving the revised manuscript by Aug 18 2026 11:59PM.

Sincerely,

Andreia Cunha, PhD

Senior Editor

PLOS Medicine

plosmedicine.org

------------------------------------------------------------

Requests from Editors:

GENERAL EDITORIAL REQUESTS

* At this stage, we ask that you include a short, non-technical Author Summary of your research to make findings accessible to a wide audience that includes both scientists and non-scientists. The Author Summary should immediately follow the Abstract in your revised manuscript. This text is subject to editorial change and should be distinct from the scientific abstract. Ideally each sub-heading should contain 2-3 single sentence, concise bullet points containing the most salient points from your study. In the final bullet point of ‘What Do These Findings Mean?’ Please include the main limitations of the study in non-technical language.

Please see our author guidelines for more information: https://journals.plos.org/plosmedicine/s/revising-your-manuscript#loc-author-summary.

* Please confirm that your title complies with PLOS Medicine's style. Your title must be nondeclarative and not a question. It should begin with main concept if possible. "Effect of" should be used only if causality can be inferred, i.e., for an RCT. Please place the study design ("A randomized controlled trial," "A retrospective study," "A modelling study," etc.) in the subtitle (ie, after a colon).

* Please confirm that your abstract complies with our requirements, including format (three sections: Background, Methods and Findings, and Conclusions) and providing all the information relevant to this study type https://journals.plos.org/plosmedicine/s/submission-guidelines#loc-abstract

* Please ensure that the Introduction ends with a clear description of the study question or hypothesis.

* Please ensure that all abbreviations are defined at first use throughout the text.

* Please confirm that all numbers presented in the abstract are present and identical to numbers presented in the main manuscript text.

GENERAL

* Please include a statement regarding use or non-use of AI tools and technologies at the end of the Methods. Please specify the name(s) and version(s) of any tool(s) used and a description of their use, including how the AI-assisted or generated content was evaluated by authors, and a clear statement of which aspects of the study, article contents, data, or supporting files were affected or generated by AI tool usage. Please also include a statement specifying who retains responsibility for the final manuscript. See our guidelines for more information: https://journals.plos.org/plosmedicine/s/ethical-publishing-practice#loc-artificial-intelligence-tools-and-technologies.

* Please review your text for claims of novelty or primacy (e.g. 'for the first time') and remove this language. In addition, please check that any use of statistical terms (such as trend or significant) are supported by the data, and if not please remove them.

* Please remove the 'conclusions' subheading from the discussion. Please also remove any other subheadings from the discussion.

* Statistical reporting: Please revise throughout the manuscript, including tables and figures.

- Please report statistical information as follows to improve clarity for the reader (95% CI [13,28]; p</=)

- Please separate upper and lower bounds with commas instead of hyphens as the latter can be confused with reporting of negative values.

- Please repeat statistical definitions (HR, CI etc.) for each set of parentheses.

* In the last sentence of the Abstract Methods and Findings section, please describe the main limitation(s) of the study's methodology.

* In the abstract, please include the important dependent variables that are adjusted for in the analyses.

FUNDING STATEMENT

* The funding statement should include: specific grant numbers, initials of authors who received each award, URLs to sponsors’ websites. Also, please state whether any sponsors or funders (other than the named authors) played any role in study design, data collection and analysis, the decision to publish, or preparation of the manuscript. If they had no role in the research, include this sentence: “The funders had no role in study design, data collection and analysis, decision to publish, or preparation of the manuscript.”

* It appears that one or more study authors is affiliated with one or more of the agencies that funded the study. Thus, the statement “The funders had no role in study design, data collection and analysis, decision to publish, or preparation of the manuscript” does not apply. Please revise the Financial Disclosure accordingly, as in "[Author name] is [author's role] at [funding agency]. The funders had no other role in study design…..”

COMPETING INTERESTS STATEMENT

* All authors must declare their relevant competing interests per the PLOS policy, which can be seen here: https://journals.plos.org/plosmedicine/s/competing-interests For authors with ties to industry, please indicate whether any of the interests has a financial stake in the results of the current study.

*Please amend your competing interests statement. Funding information should go to the relevant section and include URLs.

DATA AVAILABILITY

* PLOS defines the “minimal data set” to consist of the data set used to reach the conclusions drawn in the manuscript with related metadata and methods, and any additional data required to replicate the reported study findings in their entirety. Authors do not need to submit their entire data set, or the raw data collected during an investigation. Please submit the following data:

The values behind the means, standard deviations and other measures reported;

The values used to build graphs;

The points extracted from images for analysis.

* The Data Availability Statement (DAS) requires revision. For each data source used in your study:

a) If the data are freely or publicly available, note this and state the location of the data: within the paper, in Supporting Information files, or in a public repository (include the DOI or accession number).

b) If the data are owned by a third party but freely available upon request, please note this and state the institutional owner of the data set and contact information for data requests (institutional web or email address). Note that a study author cannot be the contact for the data.

c) If the data are not freely available, please describe briefly the ethical, legal, or contractual restriction that prevents you from sharing it. Please also include an appropriate institutional contact (institutional web or email address) for inquiries (again, this cannot be a study author).

* For studies in which a novel model is central to the manuscript's findings, as is the case here, authors are responsible for providing the source code needed to replicate the study's findings in a repository (such as GitHub, SourceForge or Bitbucket) or a cloud computing service (such as Code Ocean). Please include a citable digital object identifier (DOI) or other persistent identifier of the code (such as through linkage with Zenodo). Protection of authors’ intellectual property will not be cause for exception. Please explain in the manuscript’s Data Availability Statement how readers can access the shared code.

FIGURES

* Please provide titles and legends for all figures and tables (including those in Supporting Information files). Please define all acronyms used in each figure or table in its corresponding legend.

* Please confirm that the appropriate usage rights apply to the use of this map. Please see our guidelines for map images: https://journals.plos.org/plosmedicine/s/figures#loc-maps

* Please provide the unadjusted comparisons as well as the adjusted comparisons in all relevant Tables

* Please specify the variables controlled for in all relevant Tables

ACKNOWLEDGMENTS

* Please include an Acknowledgments section in your manuscript. The Acknowledgements should not include funder information.

ECONOMIC ANALYSES

* Please report your economic analysis according to the appropriate study design provided at http://www.equator-network.org/?post_type=eq_guidelines&eq_guidelines_study_design=economic-evaluations&eq_guidelines_clinical_specialty=0&eq_guidelines_report_section=0&s= and provide the relevant completed checklist. In the checklist please include sufficient text excerpted from the manuscript to explain how you accomplished all applicable items.

Comments from Reviewers:

Reviewer #4: The authors have thoroughly addressed all my concerns. I do not have further comments.

Any attachments provided with reviews can be seen via the following link:

[LINK]

# Author comment: Author response to Decision Letter 4

**DOI:** 10.1371/journal.pmed.1004772.r007

18 Sep 2026

Attachment Submitted filename: PLOS requests_Sept 8.docx

# Editor report: Decision Letter 4

**DOI:** 10.1371/journal.pmed.1004772.r008

**Authors:** Andreia Cunha

21 Sep 2026

Dear Dr Benjamin,

On behalf of my colleagues and the Academic Editor, Matthias Egger, I am pleased to inform you that we have agreed to publish your manuscript "A global return-on-investment of COVID-19 vaccines in the first year of the vaccination programme: A model-based economic evalulation" (PMEDICINE-D-25-03197R4) in PLOS Medicine.

Before your manuscript can be formally accepted you will need to complete some formatting changes, which you will receive in a follow up email. Please revise:

- the title to 'Global return-on-investment of COVID-19 vaccines in the first year of the vaccination programme: a model-based economic evalulation'

- the first sentence of the au summary to 'Extraordinarily rapid and large investments were made globally into developing COVID-19 vaccines during the pandemic in 2020.'

- to make the limitations of the study methodology more explicit in the last sentence of the author summary

- to include a citable digital object identifier (DOI) or other persistent identifier of the code (such as through linkage with Zenodo) in the data availability statement

- to make the competing interests statement consistent with the individual competing interest statements provided by each author

Please be aware that it may take several days for you to receive this email; during this time no action is required by you. Once you have received these formatting requests, please note that your manuscript will not be scheduled for publication until you have made the required changes.

In the meantime, please log into Editorial Manager at http://www.editorialmanager.com/pmedicine/, click the "Update My Information" link at the top of the page, and update your user information to ensure an efficient production process.

PRESS

We frequently collaborate with press offices. If your institution or institutions have a press office, please notify them about your upcoming paper at this point, to enable them to help maximise its impact. If the press office is planning to promote your findings, we would be grateful if they could coordinate with medicinepress@plos.org. If you have not yet opted out of the early version process, we ask that you notify us immediately of any press plans so that we may do so on your behalf.

We also ask that you take this opportunity to read our Embargo Policy regarding the discussion, promotion and media coverage of work that is yet to be published by PLOS. As your manuscript is not yet published, it is bound by the conditions of our Embargo Policy. Please be aware that this policy is in place both to ensure that any press coverage of your article is fully substantiated and to provide a direct link between such coverage and the published work. For full details of our Embargo Policy, please visit http://www.plos.org/about/media-inquiries/embargo-policy/.

To enhance the reproducibility of your results, we recommend that you deposit your laboratory protocols in protocols.io, where a protocol can be assigned its own identifier (DOI) such that it can be cited independently in the future. Additionally, PLOS ONE offers an option to publish peer-reviewed clinical study protocols. Read more information on sharing protocols at https://plos.org/protocols?utm_medium=editorial-email&utm_source=authorletters&utm_campaign=protocols

Thank you again for submitting to PLOS Medicine. We look forward to publishing your paper.

Sincerely,

Andreia Cunha, PhD

Senior Editor

PLOS Medicine
